# Supplementary material for: Experience of establishing and coordinating a nationwide network for bidirectional intussusception surveillance in India: lessons for multisite research studies
Source: BMJ Open. 2021 May 28;11(5):e046827. doi: 10.1136/bmjopen-2020-046827 (PMC8166592; doi:10.1136/bmjopen-2020-046827)
Supplement: Supplementary data [file bmjopen-2020-046827supp001.pdf]

Intussusception in Infants: Multisite Hospital Based Surveillance in India
Potential Study Site Institution Information

**Institution**	
**Place**	
**State**	

Dear Sir/ Madam,

We are undertaking a study for documentation of intussusception in children under-two years of age at multiple sites in India. This study aims collection of retrospective data over last five-years and prospective data over 18 months on intussusception in children.

As this study involves data retrieval for retrospective data, access to medical records is very critical. Additionally, the access to /availability of clinical case record and radiological images would be very essential for making confirmation of diagnosis.

We request you to provide the following information on facilities and infrastructure at your institute/hospital focusing on diagnosis and management of the intussusception in infants. We highly appreciate your collaboration in answering the following questions and sending them to manoj@inclentrust.org.

A. General information (Please fill the response)

(Please provide information about the concerned departments in the hospital)

Sl no	Item/ Question	Pediatrics	Pediatric surgery	Pediatric gastroenterology	Radiology
1	Number of beds				
2	Number of Specialists (Teaching faculty/ non-teaching specialists)				
3	Academic Residents (MD/DNB/MCH/Fellows)				
4	Non-academic Residents				
5	Number of children (< 5 years) with intussusception admitted in last 1 year				
6	Number of infants (< 1 year) with intussusception admitted in last 1 year				

Das MK. *BMJ Open* 2021; 11:e046827. doi: 10.1136/bmjopen-2020-046827

B. Clinical services

Sl no	Questions/ Items<br>*(Please mark √in appropriate box; mark as many apply)*	Response	
		Yes	No
**1**	**Availability of round the clock services**		
1.1	Is the pediatric service available round the clock in the hospital?		
1.2	Is the pediatric surgery service available round the clock in the hospital?		
1.3	Is the surgery service available round the clock in the hospital? (If Pediatric Surgery is not available)		
1.4	Is the Emergency Service available round the clock in the hospital?		
1.5	Is the Emergency Registration Service separate from the Central Admission Registration?		
**2**	**What is the system of Central Admission Registration?**		
2.1	Computerized/ electronic admission registration		
2.2	Manual admission registration		
2.3	Any other method<br>(Specify ……………………………………………………….)		
**3**	**What is the system of Emergency Registration System?**		
3.1	Computerized/ electronic admission registration		
3.2	Manual admission registration		
3.3	Any other method<br>(Specify ……………………………………………………….)		
**4**	**Does the institution have a Centralized Medical Record Index/ Master Patient Index/ Centralized Admission Register?**		
**5**	**If yes, how is it maintained?**		
5.1	Maintained department wise		
5.2	Maintained all departments combined		
5.3	Any other method		
**6**	**Does your hospital give Rotavirus vaccination to the infants?**		
**6.1**	If yes, is the Rotavirus vaccine available in the hospital?		
**6.2**	If yes, is the Rotavirus vaccine purchased by parents from outside?		

Das MK. *BMJ Open* 2021; 11:e046827. doi: 10.1136/bmjopen-2020-046827

C. Diagnostic services

Sl no	Questions/ Items	Response
**1**	**Availability of the services**	
1.1	How many ultrasound machines are available in the hospital?	
1.2	What is the timing of availability of the ultrasound services?	
2	**Is there a ultrasound facility/ service available in the emergency department/ casualty?**	
**3**	**Who does the ultrasound in emergency hours?** (outside the routine hours)	
3.1	Qualified Radiologist	
3.2	Resident Radiology	
3.3	Any other person (specify ……………………………………..)	
**4**	**How may of the ultrasound machines have facility for digital image storage/ archival?**	
4.1	If yes, for how long the digital images of the ultrasound tests are stored/ archived?	
4.2	Are these digital images retrievable?	
**5**	**What is the reporting system for ultrasound tests?**	
5.1	Only a written report is given	
5.2	Only the film is given	
5.3	Written report and film	
5.4	Images in form of CD/ any storage device	

D. Management of Intussusception cases

Sl no	Questions/ Items<br>*(Please mark √in appropriate box; mark as many apply)*	Response	
		**Yes**	**No**
**1**	**Surgery for intussusception**		
1.1	Does the pediatric surgery/ surgery department conduct surgery for intussusception in children?		
1.2	Does the pediatric surgery/ surgery department conduct surgery for intussusception in infants (aged < 1 years)?		
1.3	If No, where are the children/infants are usually referred? (Name of the hospital and place)		
2	**Conservative management of intussusception**		
2.1	Do the children with intussusception are managed by barium enema/ air contrast enema at this hospital?		
2.2	If yes, who does this?		
2.2.1	Radiologist		
2.2.2	Pediatric surgeon		
2.2.3	Pediatrician/ pediatric gastroenterologist		
2.2.4	Any other (specify………………………………………………..)		

E. Medical Record Archival System

The project requires screening/review of the hospitalized infants in last five years. Thus listing and retrieval of medical records are very essential. The following questions/items focus on the admission, medical record archival and retrieval process. This will allow planning the case record tracking and retrieval at the institution.

Sl no	Questions/ Items (Please mark √in appropriate box; mark as many apply)	Response		
		Yes	No	
**1**	**Is the admission registration for hospital is centralized?**			***If NO, skip to Q 3***
**2**	**If yes, what is the system of admission registration?**			
2.1	Computerized/ electronic admission registration			
2.2	Manual admission registration			
2.3	Any other method (Specify …………………………………...)			
**3**	**Does the institution have a centralized medical record index/ Master Patient Index/ Centralized Admission Register?**			***If NO, skip to Q 5***
**4**	**If yes, what all information can be retrieved from the centralized medical record index/ Master Patient Index/ centralized admission register?**			
4.1	Admission number			
4.2	Date of admission			
4.3	Department of admission			
4.4	Name			
4.5	Age			
4.6	Sex			
4.6	Diagnosis at admission			
4.7	Final diagnosis			
4.8	Outcome (discharge/ death/LAMA)			
4.9	Date of outcome			
**5**	**How the case-sheets (hard copies) are stored/ archived in your hospital/institution for in-patients cases?**			
5.1	Admission number wise			
5.2	Department wise			
5.3	Date of admission wise			
5.4	Date of discharge wise			
5.5	Any other method (specify……………………………………)			
**6**	**How are the case-sheets (hard copies) of in-patient cases indexed/ listed?**			
6.1	Computerized/ electronic listing			
6.2	Manual listing			
6.3	Any other method (specify……………..……………………..)			
6.4	Not listed			

**7**	**If computerized/ electronic indexing/ listing is done, since when (year) it is being done?**			Year
**8**	**Are the death cases archived separately?**			
**8**	**Does the institute summarize the medical records of hospitalized patients periodically according to any classification system** (ICD 10 or any other)?			
**9**	**Does the institute/hospital have case-records of hospitalized children for last five years readily accessible?**			
**10**	**Please indicate about the listing/recording the information about hospitalized children under Pediatrics Department?**			
10.1	Availability of list/ index of hospitalized children (Like in the list of admitted children maintained by Nurses/ward in-charge)			
11.2	Discharge sheets/ summary of children (copy of discharge sheet maintained by Nurses/ Residents/ ward in-charge)			
**11**	**Please indicate about the listing/recording of the information about children hospitalized under Pediatrics Surgery Department?**			
11.1	Availability of list/index of hospitalized children			
11.2	Operation Theatre Register			
11.3	Discharge sheets/ summary of children			
**12**	**Does the radiology department maintain a register/ listing of patients undergoing investigation?**			
**13**	**What all information can be retrieved from the list/recording?**			
13.1	Admission number			
13.2	Date of investigation			
13.3	Name			
13.4	Age			
13.5	Sex			
13.6	Finding/ Diagnosis			

F. Institute authority approvals

The project requires access to the case-sheets of children with intussusception admitted to your hospital and collection of clinical details about them. Please inform about the approvals from the institute authority(ies) to be obtained for the same.

**Sl no**	**Questions/ Items**	
**1**	**What all permissions from institute/department authorities are needed and the time needed for the same?**	
	Authority	Time needed
1.1		
1.2		
1.3		
1.4		

**2**	**How frequently (in months) the institute ethics committee meets?**	Monthly
**3**	**What all challenges do you anticipate in this process?**	
**4**	**How all these challenges can be addressed?**	
**5**	**Any other specific comments/ suggestions?**	

G. Investigating Team Members

Please inform about the Investigating team members from the institute along with their affiliations and contact information.

	Name	Department	Designation	Address details (including email and phone)
1				
2				
3				
4				

We thank you for your support and time taken to complete the required information.

Signature Name

Date: ……/……../…………………..

For any further clarification or information, you may contact Dr Manoj Das at manoj@inclentrust.org or (M) 9810203768; (O) 011-47730000
