# Supplementary material for: Experience of establishing and coordinating a nationwide network for bidirectional intussusception surveillance in India: lessons for multisite research studies
Source: BMJ Open. 2021 May 28;11(5):e046827. doi: 10.1136/bmjopen-2020-046827 (PMC8166592; doi:10.1136/bmjopen-2020-046827)
Supplement: Supplementary data [file bmjopen-2020-046827supp002.pdf]

Intussusception in Infants: Multisite Hospital Based Surveillance in India
Potential Study Site Institution Assessment Report (TAG Site Visit)

**Institution**	
**Place**	
**State**	
**Date of visit**	

Suggestion for the Assessor
Please meet the potential investigators from Pediatrics, Pediatric Surgery, Radiology and In-charge of Medical Record Section to obtain the desired information to be captured in this tool. This is critical to select the suitable study sites to enable quality data on intussusception cases in infants. We highly appreciate your collaboration in providing your responses to the following questions and sending them to manoj@inclentrust.org.

A. General information (Please fill the response)
(Please document the information about the hospital and concerned departments)

Sl no	Item/ Question	Response
1	Total number of beds	
2	Is the Emergency Service available round the clock? (Yes/No)	
3	Is the pediatric service available round the clock? (Yes/No)	
4	Is the pediatric surgery service available round the clock? (Yes/No)	
5	Is the Radiology service available round the clock? (Yes/No)	

B. Pediatrics Department
(Please document the information about the Pediatrics Department)

Sl no	Item/ Question	Response
1	Total number of units in the Department	
2	Total number of in-patient beds	
3	Total number of ICU/PICU beds	
4	Number of faculty/ specialists	
5	Number of senior residents	
6	Number of academic junior residents/ DNB students	
7	Number of non-academic residents	

8	Is there a pediatric gastroenterology unit/department in the hospital? (Yes/No)	
9	Does any (or >1) pediatrician specializes or focuses on pediatric gastroenterology in the hospital? (Yes/No)	
10	Is there a pediatric gastroenterology OPD/ clinic in the hospital? (Yes/No)	
11	Is there a diarrhea ward/ unit in the hospital for children? (Yes/No)	
12	How are the children with following conditions managed?	Write
12.1	Diarrhoea	
12.2	Blood in stool	
12.3	Infants/young children with pain abdomen or excessive crying	
12.4	Infants/young children with vomiting	
12.5	Infants/young children with surgical abdomen	
12.6	Infants/young children with diagnosis of intussusception	
13	What is usually done, if an infant/ child comes with an ultrasound suggesting intussusception?	Write
13.1	Ultrasound repeated	
13.2	Any additional tests done	
13.3	Admission and evaluation<br>a. Admitted to pediatrics and then transferred to pediatric Surgery/ Surgery department OR<br>b. Directly referred to pediatric Surgery/ Surgery department)	
14	How are the children with intussusception managed? (ask for barium/ air contrast enema)	
15	How are the infants with intussusception managed? (ask for barium/ air contrast enema)	

16	How many patients aged < 5 years with of intussusception were seen in last one year in this department? (approximate number)	
17	How many infants (< 1 years) with of intussusception were seen in last one year in this department? (approximate number)	
18	How many were managed by this department? (approximate number)	
19	Was any of these patients with intussusception were referred to any other hospital? (approximate number)	
20	If yes, what were the most likely reasons?	
21	For the patients getting discharged, is there a copy of the discharge sheet kept in the ward/ in the department? (Yes/No)	
22	If Yes, where is the file/ archival is made?	
23	Please review some case records of admitted infants to document the following.	
23.1	Does the hospital use any structured case record/ follow specific format for clinical history recording? (Yes/No)	
23.2	Is the immunization history recorded in the clinical history? (Yes/No)	
23.3	Is the immunization history recorded with age at vaccination? (Yes/No)	
24	Is the Rotavirus vaccine given at the hospital?	
25	Any other specific observations/ comments	

Das MK. *BMJ Open* 2021; 11:e046827. doi: 10.1136/bmjopen-2020-046827

C. Pediatric Surgery/ Surgery Department

(Please document the information about the Pediatric Surgery Department.)
(Please ask for the Surgery Department, if there is no Pediatric Surgery Department.)

Sl no	Item/ Question	Response
1	Is there a Pediatric Surgery Department in the hospital? (Yes/ No)	
2	Total number of units in the Department	
3	Total number of in-patient beds	
4	Total number of ICU/PS-ICU beds	
5	Number of faculty/ specialists	
6	Number of senior residents	
7	Number of academic junior residents/ DNB students	
8	Number of non-academic residents	
9	If there is no Pediatric Surgeon, how many Surgeons are conducting surgery in children?	
10	If there is no Pediatric Surgeon, how many Surgeons are conducting surgery in infants?	
11	What is usually done, if an infant/ child comes with an ultrasound suggesting intussusception?	Write
11.1	Ultrasound repeated	
11.2	Any additional tests done	
11.3	Admission and evaluation<br>a. Admitted to pediatrics and then transferred to pediatric Surgery/ Surgery department OR<br>b. Directly referred to pediatric Surgery/ Surgery department)	
12	How are the children with intussusception managed? (ask for barium/ air contrast enema)	
13	How are the infants with intussusception managed? (ask for barium/ air contrast enema)	
14	How many patients aged < 5 years with of intussusception were seen in last one year in this department? (approximate number)	
15	How many infants (< 1 years) with of intussusception were seen in last one year in this department? (approximate number)	

16	How many were managed by this department? (approximate number)	
17	Was any of these patients with intussusception were referred to any other hospital? (approximate number)	
18	If yes, what were the most likely reasons?	
19	Is there a register for listing operated cases? (Yes, No)	
20	If Yes, please check mark the components mentioned in the register.<br>Name: Yes No Sex: Yes No<br>Patient ID: Yes No Age: Yes No<br>Diagnosis: Yes No Date of surgery: Yes No<br>Time taken: Yes No Procedure done: Yes No<br>Outcome: Yes No Surgeon: Yes No<br>Any other:	
21	Is there a register for listing children undergone non-operative intervention (like air-contrast/barium enema) (Yes, No)	
22	If Yes, please check mark the components mentioned in the register.<br>Name: Yes No Sex: Yes No<br>Patient ID: Yes No Age: Yes No<br>Diagnosis: Yes No Date of surgery: Yes No<br>Time taken: Yes No Procedure done: Yes No<br>Outcome: Yes No Surgeon: Yes No<br>Any other:	
23	For the patients getting discharged, is there a copy of the discharge sheet kept in the ward/ in the department? (Yes, No)	
24	If Yes, where is the file/ archival is made?	
25	Please review some case records of admitted infants to document the following.	
25.1	Does the hospital use any structured case record/ follow specific format for clinical history recording? (Yes/No)	
25.2	Is the immunization history recorded in the clinical history? (Yes/No)	
25.3	Is the immunization history recorded with age at vaccination? (Yes/No)	
26	Any other specific observations/ comments	

D. Anesthesia Department and Operation Theatre

(Please document the information about the Anesthesia Department.)

Sl no	Item/ Question	Response
1	Does the anesthetist(s) in the hospital give anesthesia to children and infants? (Yes/ No)	
2	Is the OT well equipped to handle the surgery and anesthesia of infants? (Yes/ No)	
3	Is there a register for listing operated cases? (Yes, No)	
4	If Yes, please check mark the components mentioned in the register.<br>Name: Yes No Sex: Yes No<br>Patient ID: Yes No Age: Yes No<br>Diagnosis: Yes No Date of surgery: Yes No<br>Time taken: Yes No Procedure done: Yes No<br>Outcome: Yes No Surgeon: Yes No<br>Any other:	
5	Any other specific observations/ comments	

E. Radiology Department

(Please document the information about the Radiology Department.)

Slno	Item/ Question	Response
1	Total number of Ultrasound machines	
1.1	How may Ultrasound machines have option for digital image saving?	
1.2	Is the digital image saving a routine practice? (Yes/ No)	
1.3	If Yes, for how many days usually the digital images are saved?	
2	Total number of X-ray machines (for barium enema/air contrast enema)	
3	Number of fluoroscopy machine (for barium enema/air contrast enema)	
4	Number of faculty/ specialists	
5	Number of senior residents	
6	Number of academic junior residents/ DNB students	
7	What is the usual timing for ultrasound in the hospital?	From<br>To

Das MK. *BMJ Open* 2021; 11:e046827. doi: 10.1136/bmjopen-2020-046827

8	What is the arrangement for doing ultrasound beyond routine hours/ nights?	
9	Does the machine(s) used for ultrasound in emergency have facility for digital image saving?	
10	How the reports of ultrasound are given to patients?	
10.1	Only paper report (no film/ image)	
10.2	Paper report with printed paper image (no films)	
10.3	Paper report with films	
10.4	Paper report with CD with digital images	
10.5	Paper report and others available on request/ payment	
10.6	How the reports of barium enema are given to patients?	
10.7	Only paper report (no film/ image)	
10.8	Paper report with films	
10.9	Paper report with CD with digital images	
10.10	Paper report and others (film/CD) on request/ payment	
11	How many patients aged < 5 years with of intussusception were seen in last one year in this department? (approximate number)	
12	How many infants (< 1 years) with of intussusception were seen in last one year in this department? (approximate number)	
13	Do you manage the infants/ children with intussusception by barium/ air contrast enema? (Yes/ No)	
14	If Yes, when was the last such case managed?	
15	Does the department keep a copy of the ultrasound report for record? (Yes, No)	
16	If Yes, where is the file/ archival is made?	
17	Does the radiology department maintain a register/ listing of patients undergoing investigation (ultrasound/ barium)?	
18	What all information can be retrieved from the list/recording?	
18.1	Admission number	
18.2	Date of investigation	
18.3	Name	
18.4	Age	
18.5	Sex	
18.6	Findings/ Diagnosis	
19	Any other specific observation/ comment	

F. Case Registration System

(Please document about the case registration system of the hospital for in-patient cases)

Sl no	Questions/ Items	Response (Yes/No)
1	What is the system of Central In-patient Admission Registration?	
1.1	Computerized/ electronic admission registration	
1.2	Manual admission registration	
1.3	Any other method<br>(Specify …………………………………………………….)	
2	If the Registration system is manual, how is the Registration Index maintained?	
2.1	Maintained department wise	
2.2	Maintained all departments combined	
2.3	Any other method	
3	Is the Central In-patient Admission Registration system available round the clock?	
4	Is the Emergency Case Registration System separate from the Central in-patient registration?	
5	If Yes, What is the system of Emergency Registration System?	
5.1	Computerized/ electronic admission registration	
5.2	Manual admission registration	
5.3	Any other method<br>(Specify …………………………………………………….)	
6	What all information can be retrieved from the Central In-patient Admission Registration Information? (Yes/No)	
6.1	Admission number	
6.2	Date of admission	
6.3	Department of admission	
6.4	Name	
6.5	Age	
6.6	Sex	
6.7	Diagnosis at admission	
6.8	Final diagnosis	
6.9	Outcome (discharge/ death/LAMA)	
6.10	Date of outcome	
7	Any specific observation/ comments	

Das MK. *BMJ Open* 2021; 11:e046827. doi: 10.1136/bmjopen-2020-046827

G. Medical Record Archival System

The project requires screening/review of the hospitalized infants in last five years. Thus listing and retrieval of medical records are very essential. The following questions/items focus on the admission, medical record archival and retrieval process. This will allow planning the case record tracking and retrieval at the institution.

Sl no	Questions/ Items	Response (Yes/No)
1	Does your hospital have a Medical records Department/ Division?	
2	How frequently the in-patient case-sheets (hard copies) are sent to Medical Records Department/ Division from the departments?	
3	How the in-patient case-sheets (hard copies) are stored/ archived in your hospital/institution?	
3.1	Admission number wise	
3.2	Department wise	
3.3	Date of admission wise	
3.4	Date of discharge wise	
3.5	Any other method of organization (specify………………………………………………)	
4	How are the in-patient case-sheets (hard copies) indexed/ listed?	
4.1	Computerized/ electronic listing	
4.2	Manual listing	
4.3	Any other method (specify……………………………………………………..)	
4.4	Not listed	
5	If computerized/ electronic indexing/ listing is done, since when (year) it is being done?	Year
6	Does the institute summarize the medical records of hospitalized patients periodically according to any classification system (ICD 10 or any other)?	
7	If yes, who does the classification labeling of the cases?	
8	How frequently the classification labeling of the cases is done?	
9	Is there any policy by the hospital regarding duration of archival/ storage of the in-patient case-sheets (hard copies)?	
10	If Yes, for how many years the hard copies are stored?	

11	Is there any effort for digital storage of the in-patient case-sheets?	
12	If yes, since when? (year)	
13	What is the process of retrieval of the case records from the Medical Records Department? (please describe focusing on the following)<br>a. The process of application:<br>b. Time needed for approval<br>c. Time needed for retrieval and receipt of the case records<br>d. Any limitation on the number of case records issued in one go	
14	How can we retrieve the list of children admitted with diarrhea to the hospital in month of June 2010? (please ask the concerned official for demonstrating the same)	
15	If retrieved, please document the number of children admitted with diarrhea in the month of June 2010.	
16	Any other challenge in retrieval of the in-patient case-sheets (hard copies) for last five years for the pediatrics and pediatric surgery departments?	
17	Any specific observation/ comments	

H. Institute authority and regulatory approvals

The project requires retrieval of the case-sheets of children with intussusception admitted to your hospital and collection of clinical details about them. Please inform about the approvals from the institute authority(ies) to be obtained for the same.

Sl no	Questions/ Items	
**1**	**What all permissions from institute/department authorities are needed and the time needed for the same?**	
	Authority	Time needed
1.1		
1.2		
1.3		
1.4		
1.5		
**2**	**How frequently (in months) the institute ethics committee meets?**	
**3**	**Meeting with Department Head and Faculty Colleagues**<br>**What is your assessment about the reputation of the potential investigator and support anticipated from the department?**	
**4**	**Meeting with Institute Head or Research Head**<br>**What is your assessment about the research environment in the institute and facilitations anticipated from the department?**	
**5**	**What all challenges do you anticipate in this process?**	

**6**	**How all these challenges can be addressed/mitigated?**
**7**	**Any other specific comments/ suggestions?**

I. Investigating Team Members

Please inform about the Investigating team members from the institute along with their affiliations and contact information.

	Department	Name	Designation	Address details (including email and phone)
1	Pediatrics			
2	Pediatric Surgery			
3	Radiology			
4	Medical Records			
5	Any other			

We thank you for your support and time taken to complete the required information.

Any other observations/ comments

Signature Name

Date: ……/……../…………………..

Das MK. *BMJ Open* 2021; 11:e046827. doi: 10.1136/bmjopen-2020-046827
