# Supplementary material for: Experience of establishing and coordinating a nationwide network for bidirectional intussusception surveillance in India: lessons for multisite research studies
Source: BMJ Open. 2021 May 28;11(5):e046827. doi: 10.1136/bmjopen-2020-046827 (PMC8166592; doi:10.1136/bmjopen-2020-046827)
Supplement: Supplementary data [file bmjopen-2020-046827supp003.pdf]

Supplementary document 3:

The International Classification of Diseases (ICD) codes for retrieval of the cases from the medical records section

Clinical conditions considered as suspected cases	Codes	
	ICD 10	ICD 9
Intussusception	K56.1	560.0
Volvulus	K56.2	560.2
Gallstone ileus	K56.3	560.31
Other impaction of intestine	K56.4	560.30
Intestinal adhesions with obstruction	K56.5	560.81
Other and unspecified intestinal obstruction	K56.6	560.9
Ileus, unspecified	K56.7	560.1
Paralytic ileus	K56.0	

ICD: International Classification of Diseases
