# Supplementary material for: Experience of establishing and coordinating a nationwide network for bidirectional intussusception surveillance in India: lessons for multisite research studies
Source: BMJ Open. 2021 May 28;11(5):e046827. doi: 10.1136/bmjopen-2020-046827 (PMC8166592; doi:10.1136/bmjopen-2020-046827)
Supplement: Author's manuscript [file bmjopen-2020-046827.draft_revisions.pdf]

BMJ Open is committed to open peer review. As part of this commitment we make the peer review history of every article we publish publicly available.

When an article is published we post the peer reviewers' comments and the authors' responses online. We also post the versions of the paper that were used during peer review. These are the versions that the peer review comments apply to.

The versions of the paper that follow are the versions that were submitted during the peer review process. They are not the versions of record or the final published versions. They should not be cited or distributed as the published version of this manuscript.

BMJ Open is an open access journal and the full, final, typeset and author-corrected version of record of the manuscript is available on our site with no access controls, subscription charges or pay-per-view fees (http://bmjopen.bmj.com).

If you have any questions on BMJ Open's open peer review process please email info.bmjopen@bmj.com

BMJ Open

Experience of establishing and coordinating intussusception surveillance network in India: Lessons for multisite research studies

Journal:	*BMJ Open*
Manuscript ID	bmjopen-2020-046827
Article Type:	Original research
Date Submitted by the Author:	11-Nov-2020
Complete List of Authors:	Study Group, The INCLEN Intussusception Surveillance; The INCLEN Trust International<br>Das, Manoja; The INCLEN Trust International, The INCLEN Trust International
Keywords:	PUBLIC HEALTH, Community child health < PAEDIATRICS, EPIDEMIOLOGY

SCHOLARONE™
Manuscripts

I, the Submitting Author has the right to grant and does grant on behalf of all authors of the Work (as defined in the below author licence), an exclusive licence and/or a non-exclusive licence for contributions from authors who are: i) UK Crown employees; ii) where BMJ has agreed a CC-BY licence shall apply, and/or iii) in accordance with the terms applicable for US Federal Government officers or employees acting as part of their official duties; on a worldwide, perpetual, irrevocable, royalty-free basis to BMJ Publishing Group Ltd ("BMJ") its licensees and where the relevant Journal is co-owned by BMJ to the co-owners of the Journal, to publish the Work in this journal and any other BMJ products and to exploit all rights, as set out in our licence.

The Submitting Author accepts and understands that any supply made under these terms is made by BMJ to the Submitting Author unless you are acting as an employee on behalf of your employer or a postgraduate student of an affiliated institution which is paying any applicable article publishing charge ("APC") for Open Access articles. Where the Submitting Author wishes to make the Work available on an Open Access basis (and intends to pay the relevant APC), the terms of reuse of such Open Access shall be governed by a Creative Commons licence – details of these licences and which Creative Commons licence will apply to this Work are set out in our licence referred to above.

Other than as permitted in any relevant BMJ Author's Self Archiving Policies, I confirm this Work has not been accepted for publication elsewhere, is not being considered for publication elsewhere and does not duplicate material already published. I confirm all authors consent to publication of this Work and authorise the granting of this licence.

For peer review only

Original article

Title: Experience of establishing and coordinating intussusception surveillance network in India: Lessons for multisite research studies

Short title: Experience from multisite intussusception surveillance in India

Author Information

The INCLEN Intussusception Surveillance Network Study Group

The INCLEN Trust International, Okhla Industrial Area, Phase 1, New Delhi -110020, India

E-mail: drmanojkdas@gmail.com

Corresponding Author

Manoja Kumar Das

Director Projects, The INCLEN Trust International, F1/5, Okhla Industrial Area, Phase 1, New Delhi -110020, INDIA

Phone: Off: +91-11-47730000; Mob: +91-9810203768; Fax: +91-11-47730001

E-mail: manoj@inclentrust.org

Word count: Abstract- 271 words; Main document- 2995 words

For peer review only

Author information

'The INCLEN Intussusception Surveillance Network Study Group' list of authors

1. Manoja Kumar Das

   Director Projects, The INCLEN Trust International, New Delhi, India

   Email: manoj@inclentrust.org

2. Narendra Kumar Arora

   Executive Director, The INCLEN Trust International, New Delhi, India

   Email: nkarora@inclentrust.org

3. Arindam Ray

   Senior Program Officer, Bill and Melinda Gates Foundation, India Country Office, New Delhi, India

   Email: arindam.ray@gatesfoundation.org

4. Ashish Wakhlu

   Professor, Department of Paediatric Surgery, King George's Medical University, Lucknow, Uttar Pradesh, India

   Email: ashish_wakhlu@hotmail.com

5. Bhadresh R Vyas

   Professor, Department of Paediatrics, MP Shah Government Medical College, Jamnagar, Gujarat, India

   Email: bhadreshrvyas@yahoo.co.uk

6. Javeed Iqbal Bhat

   Assistant Professor, Department of Paediatrics, Sher-I-Kashmir Institute of Medical Sciences, Srinagar, Jammu & Kashmir, India

   Email: drjaveediqbal@gmail.com

7. Jayanta K. Goswami

For peer review only - http://bmjopen.bmj.com/site/about/guidelines.xhtml

Professor, Department of Paediatric Surgery, Gauhati Medical College, Guwahati, Assam, India

Email: jayantagoswami@hotmail.com

8. John Mathai

Professor, Department of Paediatrics, PSG Institute of Medical Sciences, Coimbatore, Tamil Nadu, India

Email: psg_peds@yahoo.com

9. Kameswari K.

Professor, Department of Paediatric Surgery, Andhra Medical College, Vishakhapatnam, Andhra Pradesh, India

Email: kameswari1956@gmail.com

10. Lalit Bharadia

Consultant Paediatric Gastroenterologist, Fortis Escorts Hospital, Jaipur, Rajasthan, India

Email: lalitbharadia@gmail.com

11. Lalit Sankhe

Assistant Professor, Department of Community Medicine, Grant Medical College & JJ Hospital, Mumbai, Maharashtra, India

Email: sankhelalit@yahoo.com

12. Ajaya Kumar M.K.

Professor, Department of Paediatric Surgery, Government Medical College & SAT Hospital, Thiruvananthapuram, Kerala, India

Email: drajaykumarmk@gmail.com

13. Neelam Mohan

Consultant Paediatrics Gastroenterology, Medanta—The Medicity, Gurgaon, Haryana, India

For peer review only

Email: drneelam@yahoo.com

14. Pradeep K. Jena

Professor, Department of Paediatric Surgery, SCB Medical College, Cuttack, Odisha, India

Email: drpkjena@gmail.com

15. Rachita Sarangi

Professor, Department of Paediatrics, IMS & SUM Medical College & Hospital, Bhubaneswar, Odisha, India

Email: rachitapaedia@gmail.com

16. Rashmi Shad

Consultant Paediatrics, Choithram Hospital and Research Centre, Indore, Madhya Pradesh, India

Email: drrashmishad@yahoo.com

17. Sanjib K. Debbarma

Associate Professor, Department of Paediatrics, Agartala Government Medical College, Agartala, Tripura, India

Email: dr_sanjibdb@rediffmail.com

18. Shyamala J.

Consultant Paediatrics, Apollo Hospitals, Chennai, Tamil Nadu

Email: jaymoorthy@hotmail.com

19. Simmi K. Ratan

Professor, Department of Paediatric Surgery, Maulana Azad Medical College, Delhi, India

Email: drjohnsimmi@yahoo.com

20. Suman Sarkar

Assistant Professor, Department of Paediatrics, Institute of Post Graduate Medical Education and Research, Kolkata, West Bengal, India

For peer review only - http://bmjopen.bmj.com/site/about/guidelines.xhtml

Email: dr.sumansarkar@gmail.com

21. Vijayendra Kumar

Professor, Department of Paediatric Surgery, Indira Gandhi Institute of Medical Sciences, Patna, Bihar, India

Email: drvijayendrakr@rediffmail.com

22. Anand P. Dubey

Professor, Department of Paediatrics, Maulana Azad Medical College, Delhi, India

Email: Anand_dubey52@hotmail.com

23. Atul Gupta

Consultant Paediatric Surgery, Fortis Escorts Hospital, Jaipur, Rajasthan, India

Email: atulgupta26878@gmail.com

24. Bikasha Bihary Tripathy

Associate Professor, Department of Paediatric Surgery, IMS & SUM Medical College & Hospital, Bhubaneswar, Odisha, India

Email: bbtripathy.dr@gmail.com

25. Cenita J. Sam

Professor, Department of Paediatric Surgery, PSG Institute of Medical Sciences, Coimbatore, Tamil Nadu, India

Email: cenitasivamani@yahoo.com

26. Gowhar Nazir Mufti

Assistant Professor, Department of Paediatric Surgery, Sher-I-Kashmir Institute of Medical Sciences, Srinagar, Jammu & Kashmir, India

Email: gowharmufti@yahoo.co.in

27. Harsh Trivedi

For peer review only

Professor, Department of Paediatric Surgery, MP Shah Government Medical College, Jamnagar, Gujarat, India

Email: trivediharsh@indiatimes.com

28. Jimmy Shad

Consultant Paediatric Surgery, Apollo Hospitals, Chennai, Tamil Nadu, India

Email: shadjimmy@yahoo.co.in

29. Kaushik Lahiri

Consultant, Department of Paediatric Surgery, Gauhati Medical College, Guwahati, Assam, India

Email: Kaushik_1671979@rediffmail.com

30. Meera Luthra

Consultant Paediatric Surgery, Medanta- The Medicity, Gurgaon, Haryana, India

Email: meera.luthra@medanta.org

31. Padmalatha P

Professor, Department of Paediatrics, Andhra Medical College, Vishakhapatnam, Andhra Pradesh, India

Email: padmap_2000@yahoo.com

32. Rakesh Kumar

Associate Professor, Department of Paediatrics, Indira Gandhi Institute of Medical Sciences, Patna, Bihar, India

Email: drjaiswalrakesh@gmail.com

33. Ruchirendu Sarkar

Professor, Department of Paediatric Surgery, Institute of Post Graduate Medical Education and Research, Kolkata, West Bengal, India

Email: ruchirendu@gmail.com

For peer review only

34. Santosh Kumar A.

Professor, Department of Paediatrics, Government Medical College & SAT Hospital, Thiruvananthapuram, Kerala, India

Email: hod.pediatrics.sath@gmail.com

35. Subrat Kumar Sahoo

Associate Professor, Department of Paediatric Surgery, IMS & SUM Medical College & Hospital, Bhubaneswar, Odisha, India

Email: dr_subratsahoo@yahoo.com

36. Sunil K. Ghosh

Associate Professor, Department of Pediatric Surgery, Agartala Government Medical College, Agartala, Tripura, India

Email: sunilkrghosh752@gmail.com

37. Sushant Mane

Assistant Professor, Department of Paediatrics, Grant Medical College & JJ Hospital, Mumbai, Maharashtra, India

Email: drsush2006@gmail.com

38. Bashir Ahmad Charoo

Professor, Department of Paediatrics, Sher-I-Kashmir Institute of Medical Sciences, Srinagar, Jammu & Kashmir, India

Email: charoobash@gmail.com

39. Rajendra Prasad G.

Professor, Department of Paediatric Surgery, Andhra Medical College, Vishakhapatnam, Andhra Pradesh, India

Email: dr.grajendraprasad@gmail.com

40. Harish Kumar S

For peer review only - http://bmjopen.bmj.com/site/about/guidelines.xhtml

Paediatrics Radiologist, Apollo Hospitals, Chennai, Tamil Nadu, India

Email: drharishkumar.s@gmail.com

41. Jothilakshmi K.

Professor, Department of Paediatrics, PSG Institute of Medical Sciences, Coimbatore, Tamil Nadu, India

Email: drjothi.cbe@gmail.com

42. Nihar Ranjan Sarkar

Associate Professor, Department of Radiology, Institute of Post Graduate Medical Education and Research, Kolkata, West Bengal, India

Email: dr.niharsarkar@gmail.com

43. Pavai Arunachalam

Professor, Department of Paediatric Surgery, PSG Institute of Medical Sciences, Coimbatore, Tamil Nadu, India

Email: pavai4321@yahoo.com

44. Satya S.G. Mohapatra

Professor, Department of Radiology, IMS & SUM Medical College & Hospital, Bhubaneswar, Odisha, India

Email: drartifact@gmail.com

45. Saurabh Garge

Consultant Paediatric Surgery, Choithram Hospital and Research Centre, Indore, Madhya Pradesh, India

Email: saurabhgarge8@gmail.com

For peer review only

Abstract

Introduction: Multisite studies experience several challenges involving patient recruitment, protocol adherence, data quality and coordination related challenges. Several of these challenges are results of poor site selection and quality assurance measures. There is limited documentation of these processes and guidelines for conduct of multisite studies in developing countries like India.

Material and methods: The study sites were selected through a systematic and objective four-step selection process. The multilayer quality assurance measures were adopted to document protocol adherence and data collection quality.

Results: The site selection process involved four steps including shortlisting of potential institutions, information seeking and telephonic interaction, site visits and site selection using objective criteria. Out of over 400 hospitals screened across India, 40 potential institutions were shortlisted. Information was sought from these institutes by questionnaire and interaction with investigators done. Following visits to 25 institutes, 19 sites were selected to participate in the study. The multi-step selection process allowed filtering and identification of sites with adequate capacity and motivated investigators. The multilayer quality assurance measures monitored and ensured protocol adherence, complete record retrieval and data completeness. The key challenges experienced included time taken for obtaining regulatory and ethical approvals, which delayed completion of the study. Ten sites continued with another multisite vaccine safety surveillance study.

Conclusion: The experience and results of this systematic and objective site selection method in India are promising. The systematic multi-step site selection and data quality assurance methods presented here are feasible and practical, which can assist in conducting multisite studies in India and developing countries. The planning of such multisite studies need appropriate consideration of the time needed for regulatory and administrative approvals.

Keywords: Surveillance, Multisite study, Site selection, Quality assurance, Coordination, Challenges, Intussusception, India

For peer review only

Article summary

Strengths and limitations of this study

- Provides a systematic study site selection process using objective methodology
- Documents the experience with prospective and retrospective surveillance data collection with multi-layered data quality assurance process
- The study site selection and quality assurance measures may serve as reference for suitable adaptation in different research topics and contexts
- The lessons are applicable only to the multisite studies.
- The documentation of contractual and financial management challenges may be limited.

For peer review only

For peer review only - http://bmjopen.bmj.com/site/about/guidelines.xhtml

Introduction

Intussusception is an acute emergency in children and most commonly occurs in infants aged 4-10 months (1)(2). Although some are transient and resolve spontaneously, if not intervened timely, it may lead to bowel ischemia and perforation and may even be fatal (1). Intussusception has been reported as an adverse reaction with rotavirus vaccines (RVV) with variable risks ranging from no increased risk to low risk (1-2 additional cases per 100,000 vaccinated infants) across different countries with different RVVs (3)(4)(5)(6)(7)(8)(9)(10)(11)(12). The intussusception background rate varies widely across different countries, 9 (Bangladesh) to 328 (South Korea) per 100,000 infants (13). Limited information from India reported its incidence between 17.7 to 254 cases per 100,000 child-years (14)(15). Several other studies were single or few centre studies and varied in case definitions, age groups, reference periods, and methodology (12)(13)(14). In view of the vaccine safety concern and limited information on intussusception in India, the National Technical Advisory Group on Immunization recommended vaccine safety surveillance along with RVV introduction (16). This surveillance network aimed to generate information on intussusception among children in India to inform the policy and program related to RVV introduction and future surveillance. The objectives were to: (1) establish a surveillance network of public and private hospitals ensuring regional representation and data capturing system; (2) undertake retrospective surveillance to document the intussusception epidemiology over past five years; (3) undertake prospective surveillance to document the intussusception epidemiology over 18 months and potential linkage with RVVs; and (4) build capacity of the investigators and institutions.

Multicentre studies face several challenges related to design, site selection, regulatory approvals, study conduct, coordination, data management and dissemination (17)(18)(19)(20). The lessons from multisite studies in India are limited. The intussusception surveillance study

For peer review only - http://bmjopen.bmj.com/site/about/guidelines.xhtml

has been successfully completed and the network has evolved over time to undertake more vaccine safety studies. The purpose of this paper is to share the process of network establishment, coordination, data management and lessons learned from such nationwide network research.

Methods

Study design: This study combined both retrospective and prospective surveillance.

Study governance: The Central Coordinating Unit (CCU) constituted investigators, research staffs and administrative and finance team. For technical integrity and implementation monitoring, a Technical Advisory Group (TAG) was constituted with 17 Indian and international experts in vaccine safety, surveillance, immunization program, public health, child health, paediatric surgery, radiology and medical record system, representation from Ministry of Health and Indian Council of Medical Research.

Site selection: We followed a four-step systematic study site selection process (Figure 1). *Step 1 (Shortlisting):* We categorised the states into four regions (north, south, east and west) and screened the tertiary care hospitals (medical colleges and private hospitals) in these regions from the websites. *Step 2 (Screening):* We solicited information about the case load, clinical and diagnostic capacities (paediatrics, paediatric surgery, and radiology), medical record system, ethical and administrative approvals from the shortlisted institutions using questionnaire and telephonic interactions (Supplementary document 1). *Step 3 (Evaluation):* A TAG member visited the institutions to assess the institution capacity (clinical facility, case documentation, research support system, medical record-keeping system) and interacted with the potential investigator(s), department and institution leaderships to assess the commitment, institution support and research environment (Supplementary document 2). *Step 4 (Site*

For peer review only - http://bmjopen.bmj.com/site/about/guidelines.xhtml

finalisation): Based on the questionnaire, interaction with investigators and TAG member feedback, the study sites were finalised.

Protocol finalisation and study tool development: In multisite research projects, consensus building among the investigators on the study protocol, case record forms (CRFs), data management and monitoring are critical. The study protocol and CRFs were shared and piloted at the study sites. The protocol, study tools, implementation, data management and monitoring were discussed in detail during protocol finalisation workshop involving lead investigators from all sites and TAG members.

Regulatory procedures: As this study involved international funding, the approval from Health Ministry Screening Committee (HMSC) was obtained. The study protocol documents were submitted to the participating institutes for ethical approvals. Following the ethical approval, the study was implemented at the sites.

Study implementation process: Before implementation of the study at the sites, the following four items had to be completed: (1) institute administrative approval; (2) ethical approval; (3) agreement executed with the institution; and (4) research staff selected and trained.

Data collection, management and monitoring: Separate CRFs and log sheets were prepared for the retrospective and prospective data collection. The retrospective data collection involved multiple sources; case records, registers from different departments and operation theatres and medical records section. For case record retrieval, the sites following International Classification of Diseases (ICD, ICD-9 or ICD-10) system, the ICD codes for intussusception or acute abdomen conditions were used (published in methodology paper) (21). At the other sites, cases were identified as per the diagnoses. For the prospective data collection, all admitted children were screened to identify the suspected cases, who were followed till final diagnosis. The children with intussusception were recruited and data collected. A weekly reporting from the sites was solicited for tracking progress. Initially a weekly call with the

For peer review only - http://bmjopen.bmj.com/site/about/guidelines.xhtml

For peer review only

study teams and later fortnightly to monthly calls were done to monitor the progress and address challenges. Based on the data collection progress reports, a monthly bulletin was prepared indicating the progresses and data quality. The completed study tools were sent periodically to CCU. The data received at CCU was reviewed for completeness, correctness and queries were resolved with reference to the source documents. Double data entry was done using customised data entry platform with inbuilt data matching program. The data was archived in the server with authorised access and regular backup. Data analysis was done by under guidance of TAG. The data collection, flow and management followed for prospective and retrospective surveillance are shown in figure 2 and figure 3.

Quality assurance measures: Multiple quality assurance measures focused on protocol adherence and data quality. After initiation of data collection, TAG members visited each study site during the retrospective surveillance to verify: (a) protocol adherence; (b) identification of suitable cases; and (c) data abstraction. The corrections/clarifications and repeat training was imparted as per TAG observations. After six months, during the prospective surveillance, TAG member again visited to review the: (a) patient screening; (b) tracking and eligible patients identification; (c) obtaining consent; and (d) data abstraction.

Patient and Public Involvement: No patients or the public were involved in the design, conduct, reporting, and dissemination of research.

Results

Study timelines: The study was sanctioned in November 2014. The HMSC application was submitted in December 2014 and approved in May 2015. The formal study site selection initiated after the HMSC approval and was completed by August 2015. The protocol finalisation workshop was held in October 2015. Following the ethical approvals, the retrospective surveillance was initiated at majority of the sites in February 2016. The

For peer review only - http://bmjopen.bmj.com/site/about/guidelines.xhtml

prospective surveillance was initiated in April 2016 and continued through September 2017. The data entry, cleaning and analysis and report drafting were completed by June 2018.

Study site selection: Out of over 400 hospitals screened across regions, 40 potential hospitals/institutions (10 per region) were shortlisted. While preparing the database, many institutions didn't have adequate information on their websites about the facilities and faculty members or specialists. The potential investigators from these 40 shortlisted institutions were invited to submit desired information using a questionnaire via email and all responded. Telephonic discussions was held with the potential investigator(s) from these institutions (45-60 minutes) for additional information or clarifications. Based on the criteria and consultation with TAG, 25 institutes were visited. Based on the questionnaire, interactions and TAG member assessment, 19 institutions (north region- 5 sites, 3 public and 2 private; south region- 5 sites, 2 public and 3 private; east region- 6 sites, 5 public and 1 private; west region- 3 sites, 2 public and 1 private) were selected (figure 4). The study originally planned for 17 sites. Two more sites from the states where RVV was scheduled for introduction were added later. Several potential investigators requested the CCU team to discuss with their institute leadership for permission and allow him/her to lead the project. The CCU team succeeded in all expect two institutions, which couldn't be included. The site selection process was delayed by seven months due to the delay in HMSC approval.

Regulatory approvals: The ethics approvals from the sites needed average 4 months (range: 1 to 8 months). No protocol amendment was needed.

Data collection: During July 2010 and March 2016 (retrospective surveillance period), out of 42,866 admitted under-two years children, 2092 suspected cases were identified and 1588 confirmed intussusception cases were recruited (22). The cases recruited at study sites ranged from 14 to 652. While five sites documented <20 cases each, two sites contributed >100 cases each. During April 2016 and September 2017 (prospective surveillance period), out of 6300

For peer review only - http://bmjopen.bmj.com/site/about/guidelines.xhtml

hospitalised under-two years children, 1203 suspected cases were identified and 621 confirmed intussusception cases were recruited (23). The cases recruited at the study sites ranged from 5 to 191 cases. While seven sites recruited <10 cases each, three sites contributed >50 cases each. At one study site, very few cases were retrieved from the retrospective and prospective surveillance, contrary to the anticipation.

Medical record retrieval: Twelve (seven private and five public) institutions had electronic medical record (EMR) system. The medical records were accessible at all study sites except one site, where the research staff faced challenge in timely and adequate access to the medical records. The medical records required organisation at this site and thus the retrospective data collection took longer. The record retrieval was quicker at institutions with EMR system and retrospective data collection was completed in 2-4 months. At two sites the retrospective data collection took longer 6 and 10 months, due to higher case load and challenge in record retrieval, respectively.

Radiology documentation: The ultrasound digital images and report were available for all prospectively recruited cases. There were variations in the ultrasound finding documentations and details in the reports, which required clarification from the radiologist for data capturing. Ultrasound reports were available at all but one hospital, where the findings were recorded in the case sheet. Digital records of ultrasounds were stored for minimum three months to five years. These ultrasound reports, digital films and clinical records were reviewed by independent TAG members for confirmation and classification according to Brighton Collaboration Criteria (1).

Vaccination information: Majority of the parents were not carrying the vaccination card at the time of hospitalisation and about half of them came from outside the city. The research staffs pursued to obtain the vaccination card during hospitalisation and after discharge. The vaccination card after discharge was obtained through email, mobile message and also self-

For peer review only - http://bmjopen.bmj.com/site/about/guidelines.xhtml

addressed and stamped envelopes handed over to the parents. The vaccination information was available for 78.4% of the prospective cases.

Intra-and inter-department coordination: At all institutions the nurses, residents and faculty members supported the surveillance. The nurse and residents at most places informed the research staff about any confirmed case, even on holidays. If the patient was discharged on any holiday, the research staffs attended and collected the data. Intra- and inter-departmental coordination for data collection were smooth at all sites except two sites, where the participation from paediatric department was limited. As most of the intussusception cases were directly admitted to or transferred to paediatric surgery department on diagnosis, it didn't affect the case screening and recruitment.

Clinical case management: At five sites only surgery was done. At two sites reduction was available during daytime only and surgery during other period and at twelve sites both methods were available always.

Data quality assurance: TAG members made two quality assurance visits, first during the retrospective surveillance period, second during the prospective surveillance period and provided written feedback. The retrospective surveillance was at risk of missing cases. As it was difficult to compare the absolute case numbers across the centres and periods, we derived intussusception case rate per 1000 paediatric surgery admissions. After completion of one year of prospective surveillance, third quality assurance visit was made by the data management team to 11 sites with >30% differences in the case rates between retrospective and prospective periods. The team targeted verifying the confirmed cases, suspected cases and total admissions during 2015, 2016, and 2017 (until visit). The team identified three missed cases (one each at three sites).

Investigators commitment and transition: Despite the systematic selection process, the involvement of the investigators in the day-to-day operations and data verification was lesser

For peer review only - http://bmjopen.bmj.com/site/about/guidelines.xhtml

than expected at three sites. At three sites the lead investigators transitioned without any impact on the study activities.

Research staff issues: Although one research staff per site was planned, at seven sites two research staffs were engaged considering the efforts needed for data retrieval and case load. These research staffs were trained through regional training workshops followed by hands-on training. At eight sites the research staffs changed during the study period, once at six sites and twice at two sites. All the new staffs were trained by the CCU team member through site visit followed by virtual support. The investigators swiftly ensured replacements and no loss of cases. At CCU, there were three rounds of research staff transition.

Financial management: In view of the foreign funding source and Foreign Contribution Regulation Act (FCRA) regulation, the funds couldn't be transferred to most institutes without FCRA approval. Thus, the fund was managed by the CCU for most of the institutes. Despite no fund transferred to the institutes, agreements were executed between the institutes with the investigators as the witnesses. We experienced challenges with some institutes in explaining the FCRA obligations prior to agreement execution.

Study tenure: Although planned for 30 months, the study was completed in 43 months. The delays in HMSC and ethics approval at sites were the key reasons for the delay.

Dissemination: Four peer-reviewed manuscript have been published and eight manuscripts are under review or in preparation. The manuscripts have all the site investigators as authors, either individually or as group. The findings have been shared with the Ministry of Health and Family Welfare, World Health Organisation and other key partners. Over 22 oral presentations had been made at national, regional and international meetings.

Capacity building and transition: After this study, several sites were included in other studies coordinated by the CCU, ten sites in one study and four sites in another study. Four site

For peer review only - http://bmjopen.bmj.com/site/about/guidelines.xhtml

For peer review only

investigators collaborated in other research projects. Several of the research staffs at these sites continued with the new studies.

Discussion

This multicentre sentinel surveillance successfully generated clinical and sociodemographic epidemiology information on intussusception in children including the regional the variations. The network adopted a systematic site selection process. A common understanding among the investigators was ensured and transmitted to the research staffs for appropriate implementation. The effective coordination, data management and quality assurance measures ensured high quality data. Despite the efforts there were challenges related to the regulatory and administrative challenges, which affected the implementation timelines. The continuation of several sites and investigators in other studied demonstrated the strong collaboration and confidence coupled with capacity building.

Poor site selection can lead to delay in completion, rise in cost, protocol amendment, implementation variations or study failure (24). Studies have used different site selection processes: active hunting, peer referrals, inviting expression of interests, engaging research organisations, etc. (25)(26)(27). Our organisation has been conducting multisite network studies (ranging from 5 to 84 partner institutions) over last 15 years. We have adopted variable combination of processes for the study site and investigators selection, which have evolved over time. For this study, we adopted a systematic site selection process, which appeared lengthy and required several rounds of interactions. The four-step process assisted in identifying the highly motivated investigators and study sites with desired capacity. The potential investigators who failed to return the questionnaire with desired information or participate in the interaction are unlikely to devote time for study conduct and supervision. The systematic selection process allowed reducing the potential sites to a manageable number of

For peer review only - http://bmjopen.bmj.com/site/about/guidelines.xhtml

suitable sites that were further assessed through visit. While the process was lengthy, it allowed also to build common understanding and commitment for the study. These experiences were similar to some multicounty studies (25)(28)(29).

The procedural efforts and time taken for ethical approval from the site institutes are well known. A review observed that the study site ethics approval took from 5 to 798 days and the review process and contents varied widely (30). The ethics review process consumed sizable staff hours and budget, forcing timeline extension and budget shortfall (30)(31)(32). In our study also the documentation and formats used for submission varied widely, apart from the study protocol, CRFs and consent forms. Some of the committees asked the investigators to present their protocol, while others didn't.

Although the investigators were responsible for the conduct of the study, the agreements with institutions assisted in implementation of the study. The involvement of external experts TAG as in study site selection and monitoring facilitated standardisation and quality assurance. The additional data retrieval and verification effort at the sites documented the protocol adherence and robustness of data collection.

This is the first documentation of systematic site selection process for a multisite network in India. The positive experience from this study encouraged adoption of similar systematic site selection and data quality assurance mechanism for subsequent two vaccine network studies in India.

Conclusions

In conclusion, our experience with this systematic study site selection process was positive and satisfying. It used a four-step selection process using questionnaire, objective criteria for assessment, and interactions with the investigators and institution stakeholders with site visits to identify suitable sites with adequate capacity. The participation of motivated instigators,

For peer review only - http://bmjopen.bmj.com/site/about/guidelines.xhtml

agreement with the institutions and contributory protocol and tool development facilitated successful completion. Difficulties and delays in site initiation were primarily due to the regulatory and administrative approvals. The multistep site selection adopted is feasible and practical even in Indian context. The quality assurance processes also assisted in high quality data collection. We hope that the site selection and quality assurance processes would be informative for multisite studies in India and developing countries.

For peer review only

For peer review only - http://bmjopen.bmj.com/site/about/guidelines.xhtml

Abbreviations

BCDC Brighton collaboration diagnostic criteria

CCU Central coordinating unit

CRF Case record form

EMR Electronic medical record

FCRA Foreign Contribution Regulation Act

HMSC Health Ministry Screening Committee

ICD International Classification of Diseases

RVV Rotavirus vaccines

TAG Technical Advisory Group

Usg Ultrasound

For peer review only

Acknowledgments

We acknowledge the support from Ministry of Health and Family Welfare, Government of India for undertaking the study. We are thankful to the hospital administrations and the clinicians at the study site institutes, who supported and facilitated undertaking of the study.

We highly value the technical guidance and inputs provided by the members of Technical Advisory Group: Satinder Aneja, Anju Seth and Archana Puri, Lady Hardinge Medical College, New Delhi; Ashok Patwari, Hamdard Institute of Medical Sciences & Research, New Delhi; Yogesh Kumar Sarin, Maulana Azad Medical College, New Delhi; Rakesh Aggarwal, Anshu Srivastava and Ujjal Poddar, Sanjay Gandhi Postgraduate Institute of Medical Sciences, Lucknow; Malathi Satyasekharan, Kanchi Kamakoti Chailds Trust Hospital, Chennai; Raju Sharma and Nirupam Madan, All India Institute of Medical Sciences, New Delhi; Jyoti Joshi and Deepak Polpakara, Immunization Technical Support Unit; Ministry of Health & Family Welfare, New Delhi; Umesh D. Parashar; Centers of Disease Control and Prevention, Atlanta, USA; Naveen Thacker, Child Health Foundation, Gandhigram; and Rashmi Arora, Indian Council of Medical Research, Ansari Nagar, New Delhi.

We acknowledge the contribution of the research staffs at The INCLEN Trust International: Harshpreet Kaur, Janvi Chaubey, Mrimmaya Das, Shweta Sharma and Vaibhav Jain.

We highly appreciate the efforts made by the research staffs at the study sites: Aarezo Bashir and Rafia; Sher-e-Kashmir Institute of Medical Sciences, Srinagar, Jammu & Kashmir; Prabha Shankar, Medanta-The Medicity Hospital, Gurgaon, Haryana; Anju Sharma; Maulana Azad Medical College, New Delhi; Anita Singh and Shubhranshu Srivastava, King George Medical University, Lucknow, Uttar Pradesh; Hemant Meena, Choithram Hospital, Indore, Madhya Pradesh; Pankaj Kumar and Shashi Kant; Indira Gandhi Institute of Medical Sciences, Patna, Bihar; Goutam Benia, IMS & SUM Medical College & Hospital, Bhubaneshwar, Odisha; Prasntajyoti Mohanty, SVP Post Graduate Institute of Paediatrics, Cuttack, Odisha;

For peer review only

Angshuman Chatterjee, Institute of Postgraduate Medical Education and Research & SSKM Hospital, Kolkata, West Bengal; S. Yamuna, Andhra Medical College, Vishakhapatnam, Andhra Pradesh; Srinidhi Sudan, Apollo Hospitals, Hyderabad, Telengana; Rajesh Francis, Apollo Hospitals, Chennai, Tamil Nadu; T. Easter Chandru, PSG Institute of Medical Sciences, Coimbatore, Tamil Nadu; Deepthy R, Julie and Anju Shivkumar, Government Medical College & SAT Hospital, Thiruvananthapuram, Kerala; Archit Vaidya, Grant Medical College & JJ Hospital, Mumbai, Maharashtra; Nimesh Chouksey, MP Shah Government Medical College, Jamnagar, Gujarat; Nidhi Singh, Fortis Escorts Hospital, Jaipur, Rajasthan; Mrinmoy Gohain, Gauhati Medical College, Guwahati, Assam; Arpita Bhattachrjee, Saugat Ghosh and Tanusmita Debnath, Agartala Government Medical College, Agartala, Tripura.

For peer review only

For peer review only - http://bmjopen.bmj.com/site/about/guidelines.xhtml

Declarations

Ethics approval and consent to participate: The study protocol was reviewed and approved by all the participating institutes. The list of ethics committees of the participating institutes include: The INCLEN Independent Ethics Committee, The INCLEN Trust International, New Delhi, India (Ref No: IIEC 23; Dated June 30, 2015); Institutional Ethics Committee, King George's Medical University, Lucknow, Uttar Pradesh, India (Ref no: 7951/Ethics/R.Cell-15; Dated December 4, 2015); Institutional Ethics Committee, MP Shah Government Medical College, Jamnagar, Gujarat, India (ref no: 01/46/2016; Dated January 6, 2016); Institutional Ethics Committee, Sher-I-Kashmir Institute of Medical Sciences, Srinagar, Jammu & Kashmir, India (Ref no: 42/2015; Dated October 29, 2015); Institutional Ethics Committee, Gauhati Medical College, Guwahati, Assam, India (Ref no: MC/02/2015/274; Dated May 30, 2016); Institutional Human Ethics Committee, PSG Institute of Medical Sciences, Coimbatore, Tamil Nadu, India (Ref no 15/294; Dated November 16, 2015); Institutional Ethics Committee, King George Hospital, Andhra Medical College, Vishakhapatnam, Andhra Pradesh, India (Dated October 26, 2015); Institutional Ethics Committee, Fortis Escorts Hospital, Jaipur, Rajasthan, India (Ref no: FEHJ/IEC/15/0023; Dated September 14, 2015); Institutional Ethics Committee, Grant Medical College & JJ Hospital, Mumbai, Maharashtra, India (Ref no: IEC/Pharm/288/15; Dated November 19, 2015); Institutional Ethics Committee, Government Medical College & SAT Hospital, Thiruvananthapuram, Kerala, India (Ref no: 06/05/2015/MCT; Dated December 9, 2015); Medanta Institutional Ethics Committee, Medanta-The Medicity, Gurgaon, Haryana, India (MICR 559/2015; Dated January 21, 2016); Institutional Ethics Committee, SCB Medical College, Cuttack, Odisha, India; Institutional Ethics Committee, IMS & SUM Medical College & Hospital, Bhubaneswar, Odisha, India (Ref no 210/5/10/2015; Dated October 14, 2015); Institutional Ethics Committee; Choithram Hospital and Research Centre, Indore, Madhya Pradesh, India (Ref no: EC/Oct/15/20; Dated

For peer review only

October 27, 2015); Institutional Ethics Committee, Agartala Government Medical College, Agartala, Tripura, India; Institutional Ethics Committee- Clinical Studies, Apollo Hospitals, Chennai, Tamil Nadu (Dated October 14, 2015); Institutional Ethics Committee, Maulana Azad Medical College, Delhi, India (Ref no: F.1/IEC/MAMC/50/4/2015/308; Dated November 20, 2015); Institutional Ethics Committee, Institute of Post Graduate Medical Education and Research, Kolkata, West Bengal, India (Ref no: Inst/IEC/2016/197; Dated March 1, 2016); Institutional Ethics Committee, Indira Gandhi Institute of Medical Sciences, Patna, Bihar, India (Ref no: 1256/Acad; Dated November 11, 2016); and Ethics Committee, Apollo Hospital, Hyderabad, Telengana, India ((Dated October 13, 2015). The interviews with stakeholders were done after obtaining written informed consent.

Consent for publication: Not applicable

Availability of data and materials: This manuscript reports the processes and experience from the multisite network research. All relevant data has been reported. All data is available with the investigators and can be provided by the corresponding author upon reasonable request.

Competing interests: The authors declare that there is no competing interests and conflict of interest.

Disclosure statement: None. There is no financial interest or benefit for the authors arisen from this project or its direct application.

Funding: This project was supported by the Bill and Melinda Gates Foundation, USA to The INCLEN Trust International (grant number OPP1116433). The funder or its representative had no role in the design of the study and collection, analysis, and interpretation of data and writing the manuscript.

Author contributions:

For peer review only - http://bmjopen.bmj.com/site/about/guidelines.xhtml

Study conceptualisation, study design, protocol development, training, data analysis, interpretation, coordination, monitoring and quality assurance: MKD and NKA.

Participant recruitment and data collection: AW, BRV, JIB, JKG, JM, KK, LB, LS, AMK, NM, PKJ, RS-1, RS-2, SKD, SJ, SKR, SS, VK, APD, AG, BBT, CJS, GRP, GNM, HT, JS, KL, ML, PP, RK, RS-3, SKA, SKS, SKG, SM, BAC, RPG, HKS, JK, NRS, PA, SSGM and SG.

Manuscript preparation: MKD, NKA and AR

All authors reviewed, provided critical input and approved the final version. The content represents the views of the authors alone and do not necessarily represent the official positions of their organizations.

For peer review only - http://bmjopen.bmj.com/site/about/guidelines.xhtml

References

1. Bines JE, Kohl KS, Forster J, Zanardi LR, Davis RL, Hansen J, et al. Acute intussusception in infants and children as an adverse event following immunization: case definition and guidelines of data collection, analysis, and presentation. Vaccine. 2004 Jan 26;22(5–6):569–74.
2. World Health Organisation. Post-marketing surveillance of rotavirus vaccine safety [Internet]. 2017 [cited 2017 Sep 18]. Available from: http://apps.who.int/iris/bitstream/10665/70017/1/WHO_IVB_09.01_eng.pdf
3. Tate JE, Mwenda JM, Armah G, Jani B, Omore R, Ademe A, et al. Evaluation of Intussusception after Monovalent Rotavirus Vaccination in Africa. N Engl J Med. 2018 Apr 19;378(16):1521–8.
4. Groome MJ, Tate JE, Arnold M, Chitnis M, Cox S, de Vos C, et al. Evaluation of Intussusception After Oral Monovalent Rotavirus Vaccination in South Africa. Clin Infect Dis Off Publ Infect Dis Soc Am. 2020 Apr 10;70(8):1606–12.
5. Patel MM, López-Collada VR, Bulhões MM, De Oliveira LH, Márquez AB, Flannery B, et al. Intussusception Risk and Health Benefits of Rotavirus Vaccination in Mexico and Brazil. N Engl J Med. 2011 Jun 16;364(24):2283–92.
6. Velázquez FR, Colindres RE, Grajales C, Hernández MT, Mercadillo MG, Torres FJ, et al. Postmarketing surveillance of intussusception following mass introduction of the attenuated human rotavirus vaccine in Mexico. Pediatr Infect Dis J. 2012 Jul;31(7):736–44.
7. Carlin JB, Macartney KK, Lee KJ, Quinn HE, Buttery J, Lopert R, et al. Intussusception risk and disease prevention associated with rotavirus vaccines in Australia's National Immunization Program. Clin Infect Dis Off Publ Infect Dis Soc Am. 2013 Nov;57(10):1427–34.

For peer review only - http://bmjopen.bmj.com/site/about/guidelines.xhtml

8. Yih WK, Lieu TA, Kulldorff M, Martin D, McMahill-Walraven CN, Platt R, et al. Intussusception risk after rotavirus vaccination in U.S. infants. N Engl J Med. 2014 Feb 6;370(6):503–12.
9. Weintraub ES, Baggs J, Duffy J, Vellozzi C, Belongia EA, Irving S, et al. Risk of intussusception after monovalent rotavirus vaccination. N Engl J Med. 2014 Feb 6;370(6):513–9.
10. Stowe J, Andrews N, Ladhani S, Miller E. The risk of intussusception following monovalent rotavirus vaccination in England: A self-controlled case-series evaluation. Vaccine. 2016 12;34(32):3684–9.
11. Pérez-Vilar S, Díez-Domingo J, Puig-Barberà J, Gil-Prieto R, Romio S. Intussusception following rotavirus vaccination in the Valencia Region, Spain. Hum Vaccines Immunother. 2015;11(7):1848–52.
12. Koch J, Harder T, von Kries R, Wichmann O. Risk of Intussusception After Rotavirus Vaccination. Dtsch Arzteblatt Int. 2017 Apr 14;114(15):255–62.
13. Jiang J, Jiang B, Parashar U, Nguyen T, Bines J, Patel MM. Childhood intussusception: a literature review. PloS One. 2013;8(7):e68482.
14. Bahl R, Saxena M, Bhandari N, Taneja S, Mathur M, Parashar UD, et al. Population-Based Incidence of Intussusception and a Case-Control Study to Examine the Association of Intussusception with Natural Rotavirus Infection among Indian Children. J Infect Dis. 2009 Nov;200(s1):S277–81.
15. Jehangir S, John J, Rajkumar S, Mani B, Srinivasan R, Kang G. Intussusception in southern India: Comparison of retrospective analysis and active surveillance. Vaccine. 2014 Aug;32:A99–103.
16. Ministry of Health and Family Welfare, Government of India. Meeting Minutes: Meeting of the National Technical Advisory Group on Immunization (India) [Internet]. Ministry of

For peer review only

For peer review only - http://bmjopen.bmj.com/site/about/guidelines.xhtml

Health & Family Welfare Government of India; 2014 [cited 2020 Nov 3]. Available from: https://www.nitag-resource.org/media-center/document/2220-minutes-of-the-meeting-of-ntagi-held-in-2014

17. Coates EC, Mann-Salinas EA, Caldwell NW, Chung KK. Challenges Associated with Managing a Multicenter Clinical Trial in Severe Burns. J Burn Care Res. 2020 May 2;41(3):681–9.
18. Hagen NA, Stiles CR, Biondo PD, Cummings GG, Fainsinger RL, Moulin DE, et al. Establishing a multicentre clinical research network: lessons learned. Curr Oncol Tor Ont. 2011 Oct;18(5):e243-249.
19. Dewa CS, Durbin J, Wasylenki D, Ochocka J, Eastabrook S, Boydell KM, et al. Considering a multisite study? How to take the leap and have a soft landing. J Community Psychol. 2002 Mar;30(2):173–87.
20. Forjuoh SN. Challenges Associated with Multi-institutional Multi-site Clinical Trial Collaborations: Lessons from a Diabetes Self-Management Interventions Study in Primary Care. J Clin Trials [Internet]. 2015 [cited 2020 Nov 4];05(03). Available from: http://www.omicsgroup.org/journals/challenges-associated-with-multiinstitutional-multisite-clinical-trial-collaborations-lessons-from-a-diabetes-selfmanagement-interventions-study-in-primary-care-2167-0870-1000219.php?aid=55032
21. Das M, Arora N, Bonhoeffer J, Zuber P, Maure C. Intussusception in Young Children: Protocol for Multisite Hospital Sentinel Surveillance in India. Methods Protoc. 2018 Mar 22;1(2):11.
22. Das MK, Arora NK, Gupta B, Sharan A, Kameswari K, Padmalatha P, et al. Intussusception in children aged under two years in India: Retrospective surveillance at nineteen tertiary care hospitals. Vaccine. 2020 Oct;38(43):6849–57.

For peer review only - http://bmjopen.bmj.com/site/about/guidelines.xhtml

23. The INCLEN Intussusception Surveillance Network Study Group. Prospective surveillance for intussusception in Indian children aged under two years at nineteen tertiary care hospitals. BMC Pediatr. 2020 Dec;20(1):413.

24. Fogel DB. Factors associated with clinical trials that fail and opportunities for improving the likelihood of success: A review. Contemp Clin Trials Commun. 2018 Sep;11:156–64.

25. Hurtado-Chong A, Joeris A, Hess D, Blauth M. Improving site selection in clinical studies: a standardised, objective, multistep method and first experience results. BMJ Open. 2017 Jul;7(7):e014796.

26. Gheorghiade M, Vaduganathan M, Greene SJ, Mentz RJ, Adams KF, Anker SD, et al. Site selection in global clinical trials in patients hospitalized for heart failure: perceived problems and potential solutions. Heart Fail Rev. 2014 Mar;19(2):135–52.

27. Potter JS, Donovan DM, Weiss RD, Gardin J, Lindblad R, Wakim P, et al. Site selection in community-based clinical trials for substance use disorders: strategies for effective site selection. Am J Drug Alcohol Abuse. 2011 Sep;37(5):400–7.

28. Potter JS, Donovan DM, Weiss RD, Gardin J, Lindblad R, Wakim P, et al. Site Selection in Community-Based Clinical Trials for Substance Use Disorders: Strategies for Effective Site Selection. Am J Drug Alcohol Abuse. 2011 Sep 1;37(5):400–7.

29. Farquharson K, Murphy KA. Ten Steps to Conducting a Large, Multi-Site, Longitudinal Investigation of Language and Reading in Young Children. Front Psychol [Internet]. 2016 Mar 30 [cited 2020 Nov 4];7. Available from: http://journal.frontiersin.org/Article/10.3389/fpsyg.2016.00419/abstract

30. Greene SM, Geiger AM. A review finds that multicenter studies face substantial challenges but strategies exist to achieve Institutional Review Board approval. J Clin Epidemiol. 2006 Aug;59(8):784–90.

For peer review only

31. Green LA, Lowery JC, Kowalski CP, Wyszewianski L. Impact of institutional review board practice variation on observational health services research. Health Serv Res. 2006 Feb;41(1):214–30.

32. Maskell NA, Jones EL, Davies RJO, BTS/MRC MIST steering committee. Variations in experience in obtaining local ethical approval for participation in a multi-centre study. QJM Mon J Assoc Physicians. 2003 Apr;96(4):305–7.

For peer review only

For peer review only - http://bmjopen.bmj.com/site/about/guidelines.xhtml

Figures

Figure 1: The steps of the site selection process

Note: TAG: Technical Advisory Group

Figure 2: The data collection and management flow for prospective surveillance

Abbreviations: BCDC: Brighton collaboration diagnostic criteria; CCU: Central coordinating unit; CRF: case record form; TAG: Technical Advisory Group; Usg: Ultrasound

Figure 3: The data collection and management flow for retrospective surveillance

Abbreviations: BCDC: Brighton collaboration diagnostic criteria; CCU: Central coordinating unit; CRF: case record form; ICD: International classification of diseases; TAG: Technical Advisory Group; Usg: Ultrasound

Figure 4: The geographic distribution of the network study sites

Note: The image was created for the research project by the investigators.

For peer review only

Supplementary Documents

Supplementary document 1: Potential Study Site Institution Information

Supplementary document 2: Potential Study Site Institution Assessment Report (Site Visit)

For peer review only

For peer review only - http://bmjopen.bmj.com/site/about/guidelines.xhtml

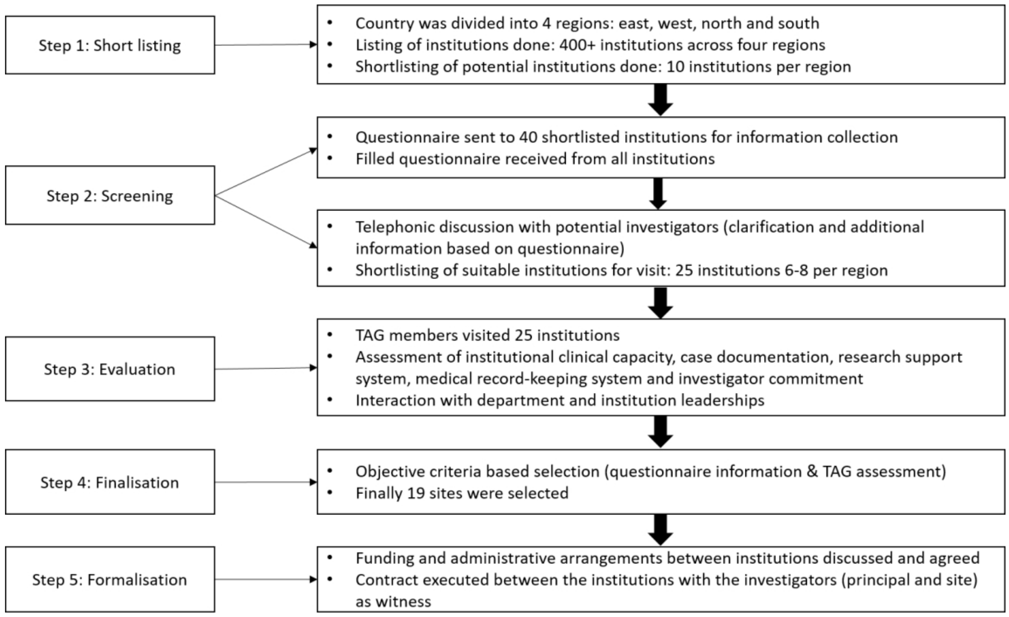

Figure 1: The steps of the site selection process

427x264mm (96 x 96 DPI)

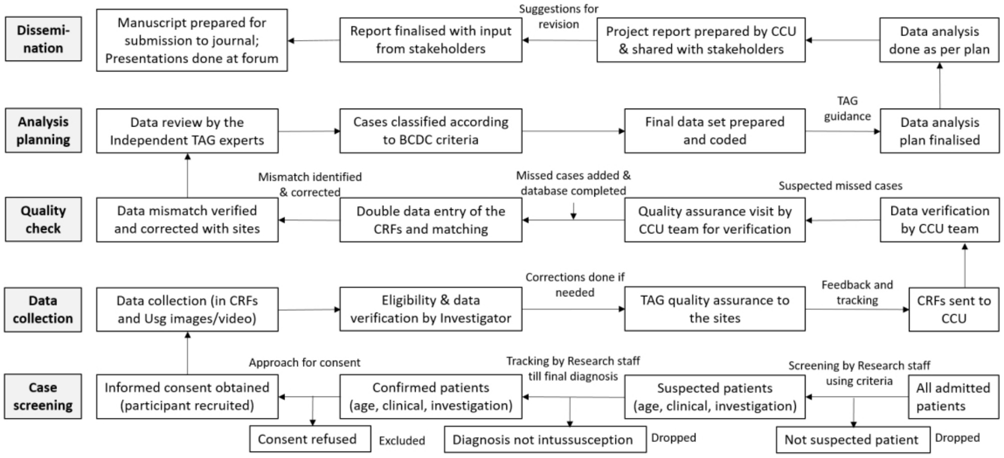

Figure 2: The data collection and management flow for prospective surveillance

516x238mm (96 x 96 DPI)

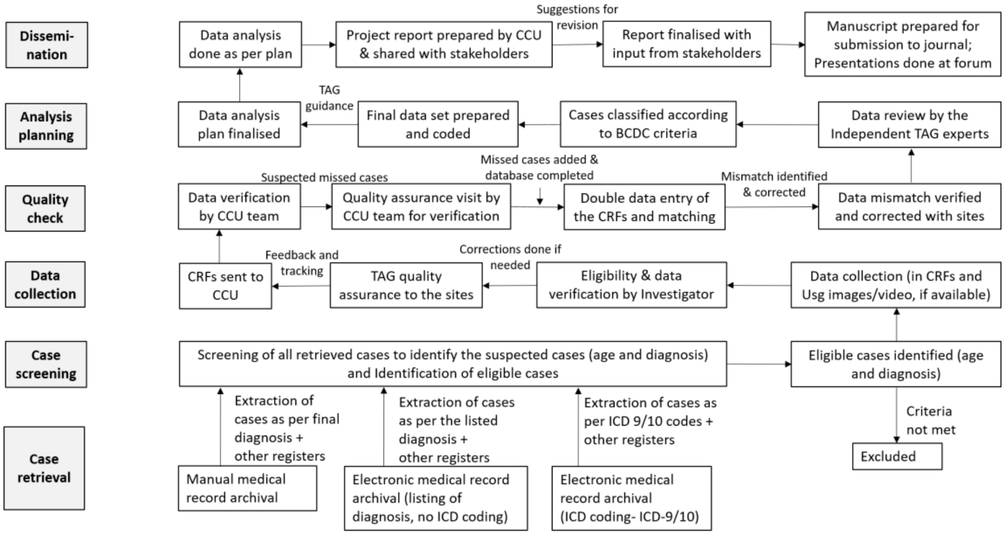

Figure 3: The data collection and management flow for retrospective surveillance

502x270mm (96 x 96 DPI)

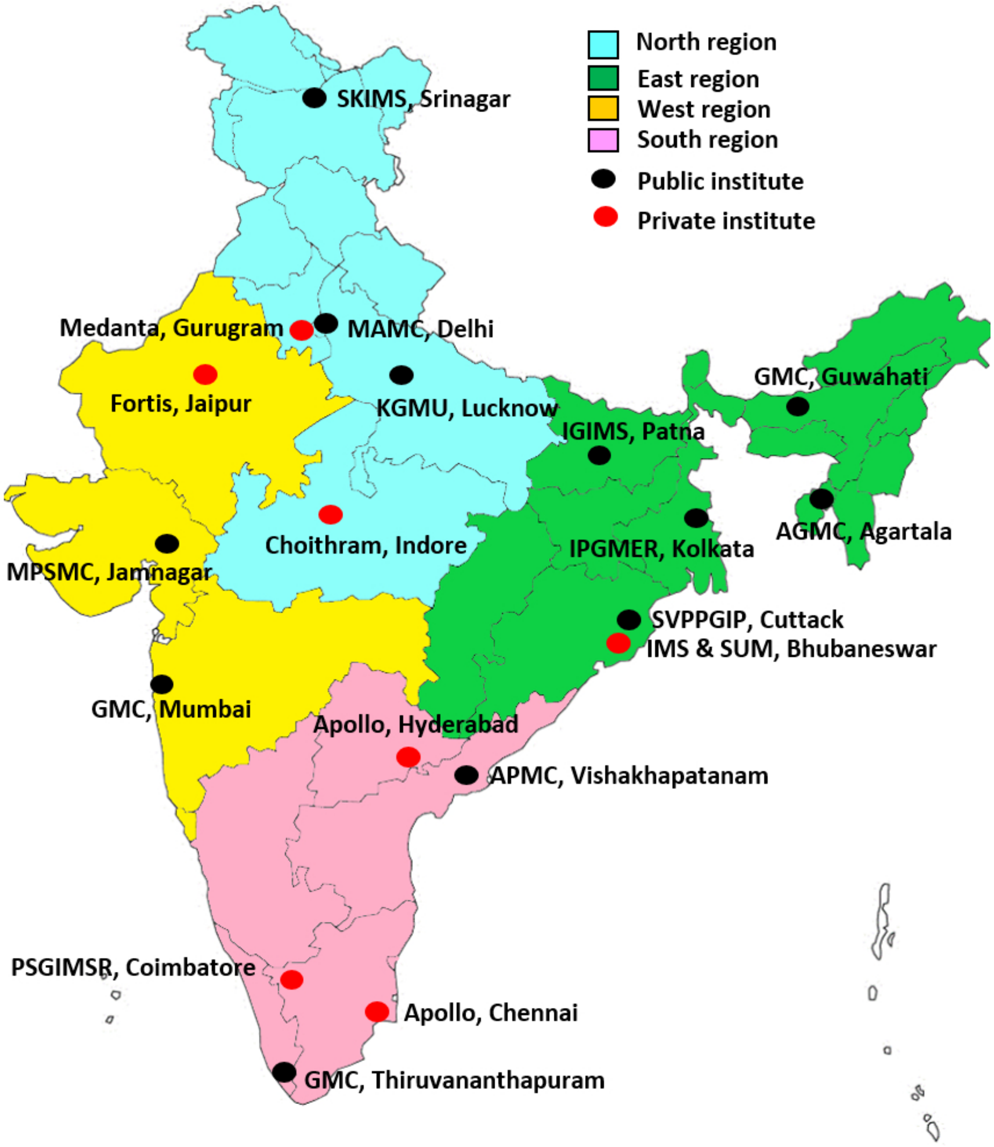

Figure 4: The geographic distribution of the network study sites

228x263mm (96 x 96 DPI)

Intussusception in Infants: Multisite Hospital Based Surveillance in India
Potential Study Site Institution Information

**Institution**	
**Place**	
**State**	

Dear Sir/ Madam,

We are undertaking a study for documentation of intussusception in children under-two years of age at multiple sites in India. This study aims collection of retrospective data over last five-years and prospective data over 18 months on intussusception in children.

As this study involves data retrieval for retrospective data, access to medical records is very critical. Additionally, the access to /availability of clinical case record and radiological images would be very essential for making confirmation of diagnosis.

We request you to provide the following information on facilities and infrastructure at your institute/hospital focusing on diagnosis and management of the intussusception in infants. We highly appreciate your collaboration in answering the following questions and sending them to manoj@inclentrust.org.

A. General information (Please fill the response)

(Please provide information about the concerned departments in the hospital)

Sl no	Item/ Question	Pediatrics	Pediatric surgery	Pediatric gastroenterology	Radiology
1	Number of beds				
2	Number of Specialists (Teaching faculty/ non-teaching specialists)				
3	Academic Residents (MD/DNB/MCH/Fellows)				
4	Non-academic Residents				
5	Number of children (< 5 years) with intussusception admitted in last 1 year				
6	Number of infants (< 1 year) with intussusception admitted in last 1 year				

For peer review only - http://bmjopen.bmj.com/site/about/guidelines.xhtml

B. Clinical services

Sl no	Questions/ Items<br>*(Please mark √in appropriate box; mark as many apply)*	Response	
		Yes	No
**1**	**Availability of round the clock services**		
1.1	Is the pediatric service available round the clock in the hospital?		
1.2	Is the pediatric surgery service available round the clock in the hospital?		
1.3	Is the surgery service available round the clock in the hospital? (If Pediatric Surgery is not available)		
1.4	Is the Emergency Service available round the clock in the hospital?		
1.5	Is the Emergency Registration Service separate from the Central Admission Registration?		
**2**	**What is the system of Central Admission Registration?**		
2.1	Computerized/ electronic admission registration		
2.2	Manual admission registration		
2.3	Any other method<br>(Specify ……………………………………………………….)		
**3**	**What is the system of Emergency Registration System?**		
3.1	Computerized/ electronic admission registration		
3.2	Manual admission registration		
3.3	Any other method<br>(Specify ……………………………………………………….)		
**4**	**Does the institution have a Centralized Medical Record Index/ Master Patient Index/ Centralized Admission Register?**		
**5**	**If yes, how is it maintained?**		
5.1	Maintained department wise		
5.2	Maintained all departments combined		
5.3	Any other method		
**6**	**Does your hospital give Rotavirus vaccination to the infants?**		
**6.1**	If yes, is the Rotavirus vaccine available in the hospital?		
**6.2**	If yes, is the Rotavirus vaccine purchased by parents from outside?		

For peer review only

C. Diagnostic services

Sl no	Questions/ Items	Response
**1**	**Availability of the services**	
1.1	How many ultrasound machines are available in the hospital?	
1.2	What is the timing of availability of the ultrasound services?	
2	**Is there a ultrasound facility/ service available in the emergency department/ casualty?**	
**3**	**Who does the ultrasound in emergency hours?** (outside the routine hours)	
3.1	Qualified Radiologist	
3.2	Resident Radiology	
3.3	Any other person (specify ………………………………………..)	
**4**	**How may of the ultrasound machines have facility for digital image storage/ archival?**	
4.1	If yes, for how long the digital images of the ultrasound tests are stored/ archived?	
4.2	Are these digital images retrievable?	
**5**	**What is the reporting system for ultrasound tests?**	
5.1	Only a written report is given	
5.2	Only the film is given	
5.3	Written report and film	
5.4	Images in form of CD/ any storage device	

D. Management of Intussusception cases

Sl no	Questions/ Items<br>*(Please mark √in appropriate box; mark as many apply)*	Response	
		**Yes**	**No**
**1**	**Surgery for intussusception**		
1.1	Does the pediatric surgery/ surgery department conduct surgery for intussusception in children?		
1.2	Does the pediatric surgery/ surgery department conduct surgery for intussusception in infants (aged < 1 years)?		
1.3	If No, where are the children/infants are usually referred? (Name of the hospital and place)		
2	**Conservative management of intussusception**		
2.1	Do the children with intussusception are managed by barium enema/ air contrast enema at this hospital?		
2.2	If yes, who does this?		
2.2.1	Radiologist		
2.2.2	Pediatric surgeon		
2.2.3	Pediatrician/ pediatric gastroenterologist		
2.2.4	Any other (specify………………………………………………..)		

For peer review only - http://bmjopen.bmj.com/site/about/guidelines.xhtml

E. Medical Record Archival System

The project requires screening/review of the hospitalized infants in last five years. Thus listing and retrieval of medical records are very essential. The following questions/items focus on the admission, medical record archival and retrieval process. This will allow planning the case record tracking and retrieval at the institution.

Sl no	Questions/ Items *(Please mark √in appropriate box; mark as many apply)*	Response		
		Yes	No	
**1**	**Is the admission registration for hospital is centralized?**			***If NO, skip to Q 3***
**2**	**If yes, what is the system of admission registration?**			
2.1	Computerized/ electronic admission registration			
2.2	Manual admission registration			
2.3	Any other method (Specify …………………………………...)			
**3**	**Does the institution have a centralized medical record index/ Master Patient Index/ Centralized Admission Register?**			***If NO, skip to Q 5***
**4**	**If yes, what all information can be retrieved from the centralized medical record index/ Master Patient Index/ centralized admission register?**			
4.1	Admission number			
4.2	Date of admission			
4.3	Department of admission			
4.4	Name			
4.5	Age			
4.6	Sex			
4.6	Diagnosis at admission			
4.7	Final diagnosis			
4.8	Outcome (discharge/ death/LAMA)			
4.9	Date of outcome			
**5**	**How the case-sheets (hard copies) are stored/ archived in your hospital/institution for in-patients cases?**			
5.1	Admission number wise			
5.2	Department wise			
5.3	Date of admission wise			
5.4	Date of discharge wise			
5.5	Any other method (specify……………………………………)			
**6**	**How are the case-sheets (hard copies) of in-patient cases indexed/ listed?**			
6.1	Computerized/ electronic listing			
6.2	Manual listing			
6.3	Any other method (specify……………..……………………..)			
6.4	Not listed			

For peer review only - http://bmjopen.bmj.com/site/about/guidelines.xhtml

**7**	**If computerized/ electronic indexing/ listing is done, since when (year) it is being done?**			Year
**8**	**Are the death cases archived separately?**			
**8**	**Does the institute summarize the medical records of hospitalized patients periodically according to any classification system** (ICD 10 or any other)?			
**9**	**Does the institute/hospital have case-records of hospitalized children for last five years readily accessible?**			
**10**	**Please indicate about the listing/recording the information about hospitalized children under Pediatrics Department?**			
10.1	Availability of list/ index of hospitalized children (Like in the list of admitted children maintained by Nurses/ward in-charge)			
11.2	Discharge sheets/ summary of children (copy of discharge sheet maintained by Nurses/ Residents/ ward in-charge)			
**11**	**Please indicate about the listing/recording of the information about children hospitalized under Pediatrics Surgery Department?**			
11.1	Availability of list/index of hospitalized children			
11.2	Operation Theatre Register			
11.3	Discharge sheets/ summary of children			
**12**	**Does the radiology department maintain a register/ listing of patients undergoing investigation?**			
**13**	**What all information can be retrieved from the list/recording?**			
13.1	Admission number			
13.2	Date of investigation			
13.3	Name			
13.4	Age			
13.5	Sex			
13.6	Finding/ Diagnosis			

F. Institute authority approvals

The project requires access to the case-sheets of children with intussusception admitted to your hospital and collection of clinical details about them. Please inform about the approvals from the institute authority(ies) to be obtained for the same.

**Sl no**	**Questions/ Items**	
**1**	**What all permissions from institute/department authorities are needed and the time needed for the same?**	
	Authority	Time needed
1.1		
1.2		
1.3		
1.4		

For peer review only - http://bmjopen.bmj.com/site/about/guidelines.xhtml

**2**	**How frequently (in months) the institute ethics committee meets?**	Monthly
**3**	**What all challenges do you anticipate in this process?**	
**4**	**How all these challenges can be addressed?**	
**5**	**Any other specific comments/ suggestions?**	

For peer review only

G. Investigating Team Members

Please inform about the Investigating team members from the institute along with their affiliations and contact information.

	Name	Department	Designation	Address details (including email and phone)
1				
2				
3				
4				

We thank you for your support and time taken to complete the required information.

Signature Name

Date: ……/……../…………………..

For any further clarification or information, you may contact Dr Manoj Das at manoj@inclentrust.org or (M) 9810203768; (O) 011-47730000

Intussusception in Infants: Multisite Hospital Based Surveillance in India
Potential Study Site Institution Assessment Report (TAG Site Visit)

**Institution**	
**Place**	
**State**	
**Date of visit**	

Suggestion for the Assessor

Please meet the potential investigators from Pediatrics, Pediatric Surgery, Radiology and In-charge of Medical Record Section to obtain the desired information to be captured in this tool. This is critical to select the suitable study sites to enable quality data on intussusception cases in infants. We highly appreciate your collaboration in providing your responses to the following questions and sending them to manoj@inclentrust.org.

A. General information (Please fill the response)

(Please document the information about the hospital and concerned departments)

Sl no	Item/ Question	Response
1	Total number of beds	
2	Is the Emergency Service available round the clock? (Yes/No)	
3	Is the pediatric service available round the clock? (Yes/No)	
4	Is the pediatric surgery service available round the clock? (Yes/No)	
5	Is the Radiology service available round the clock? (Yes/No)	

B. Pediatrics Department

(Please document the information about the Pediatrics Department)

Sl no	Item/ Question	Response
1	Total number of units in the Department	
2	Total number of in-patient beds	
3	Total number of ICU/PICU beds	
4	Number of faculty/ specialists	
5	Number of senior residents	
6	Number of academic junior residents/ DNB students	
7	Number of non-academic residents	

For peer review only - http://bmjopen.bmj.com/site/about/guidelines.xhtml

8	Is there a pediatric gastroenterology unit/department in the hospital? (Yes/No)	
9	Does any (or >1) pediatrician specializes or focuses on pediatric gastroenterology in the hospital? (Yes/No)	
10	Is there a pediatric gastroenterology OPD/ clinic in the hospital? (Yes/No)	
11	Is there a diarrhea ward/ unit in the hospital for children? (Yes/No)	
12	How are the children with following conditions managed?	Write
12.1	Diarrhoea	
12.2	Blood in stool	
12.3	Infants/young children with pain abdomen or excessive crying	
12.4	Infants/young children with vomiting	
12.5	Infants/young children with surgical abdomen	
12.6	Infants/young children with diagnosis of intussusception	
13	What is usually done, if an infant/ child comes with an ultrasound suggesting intussusception?	Write
13.1	Ultrasound repeated	
13.2	Any additional tests done	
13.3	Admission and evaluation<br>a. Admitted to pediatrics and then transferred to pediatric Surgery/ Surgery department OR<br>b. Directly referred to pediatric Surgery/ Surgery department)	
14	How are the children with intussusception managed? (ask for barium/ air contrast enema)	
15	How are the infants with intussusception managed? (ask for barium/ air contrast enema)	

For peer review only - http://bmjopen.bmj.com/site/about/guidelines.xhtml

16	How many patients aged < 5 years with of intussusception were seen in last one year in this department? (approximate number)	
17	How many infants (< 1 years) with of intussusception were seen in last one year in this department? (approximate number)	
18	How many were managed by this department? (approximate number)	
19	Was any of these patients with intussusception were referred to any other hospital? (approximate number)	
20	If yes, what were the most likely reasons?	
21	For the patients getting discharged, is there a copy of the discharge sheet kept in the ward/ in the department? (Yes/No)	
22	If Yes, where is the file/ archival is made?	
23	Please review some case records of admitted infants to document the following.	
23.1	Does the hospital use any structured case record/ follow specific format for clinical history recording? (Yes/No)	
23.2	Is the immunization history recorded in the clinical history? (Yes/No)	
23.3	Is the immunization history recorded with age at vaccination? (Yes/No)	
24	Is the Rotavirus vaccine given at the hospital?	
25	Any other specific observations/ comments	

For peer review only

C. Pediatric Surgery/ Surgery Department

(Please document the information about the Pediatric Surgery Department.)
(Please ask for the Surgery Department, if there is no Pediatric Surgery Department.)

Sl no	Item/ Question	Response
1	Is there a Pediatric Surgery Department in the hospital? (Yes/ No)	
2	Total number of units in the Department	
3	Total number of in-patient beds	
4	Total number of ICU/PS-ICU beds	
5	Number of faculty/ specialists	
6	Number of senior residents	
7	Number of academic junior residents/ DNB students	
8	Number of non-academic residents	
9	If there is no Pediatric Surgeon, how many Surgeons are conducting surgery in children?	
10	If there is no Pediatric Surgeon, how many Surgeons are conducting surgery in infants?	
11	What is usually done, if an infant/ child comes with an ultrasound suggesting intussusception?	Write
11.1	Ultrasound repeated	
11.2	Any additional tests done	
11.3	Admission and evaluation<br>a. Admitted to pediatrics and then transferred to pediatric Surgery/ Surgery department OR<br>b. Directly referred to pediatric Surgery/ Surgery department)	
12	How are the children with intussusception managed? (ask for barium/ air contrast enema)	
13	How are the infants with intussusception managed? (ask for barium/ air contrast enema)	
14	How many patients aged < 5 years with of intussusception were seen in last one year in this department? (approximate number)	
15	How many infants (< 1 years) with of intussusception were seen in last one year in this department? (approximate number)	

For peer review only - http://bmjopen.bmj.com/site/about/guidelines.xhtml

16	How many were managed by this department? (approximate number)	
17	Was any of these patients with intussusception were referred to any other hospital? (approximate number)	
18	If yes, what were the most likely reasons?	
19	Is there a register for listing operated cases? (Yes, No)	
20	If Yes, please check mark the components mentioned in the register.<br>Name: Yes No Sex: Yes No<br>Patient ID: Yes No Age: Yes No<br>Diagnosis: Yes No Date of surgery: Yes No<br>Time taken: Yes No Procedure done: Yes No<br>Outcome: Yes No Surgeon: Yes No<br>Any other:	
21	Is there a register for listing children undergone non-operative intervention (like air-contrast/barium enema) (Yes, No)	
22	If Yes, please check mark the components mentioned in the register.<br>Name: Yes No Sex: Yes No<br>Patient ID: Yes No Age: Yes No<br>Diagnosis: Yes No Date of surgery: Yes No<br>Time taken: Yes No Procedure done: Yes No<br>Outcome: Yes No Surgeon: Yes No<br>Any other:	
23	For the patients getting discharged, is there a copy of the discharge sheet kept in the ward/ in the department? (Yes, No)	
24	If Yes, where is the file/ archival is made?	
25	Please review some case records of admitted infants to document the following.	
25.1	Does the hospital use any structured case record/ follow specific format for clinical history recording? (Yes/No)	
25.2	Is the immunization history recorded in the clinical history? (Yes/No)	
25.3	Is the immunization history recorded with age at vaccination? (Yes/No)	
26	Any other specific observations/ comments	

For peer review only - http://bmjopen.bmj.com/site/about/guidelines.xhtml

D. Anesthesia Department and Operation Theatre

(Please document the information about the Anesthesia Department.)

Sl no	Item/ Question	Response
1	Does the anesthetist(s) in the hospital give anesthesia to children and infants? (Yes/ No)	
2	Is the OT well equipped to handle the surgery and anesthesia of infants? (Yes/ No)	
3	Is there a register for listing operated cases? (Yes, No)	
4	If Yes, please check mark the components mentioned in the register.<br>Name: Yes No Sex: Yes No<br>Patient ID: Yes No Age: Yes No<br>Diagnosis: Yes No Date of surgery: Yes No<br>Time taken: Yes No Procedure done: Yes No<br>Outcome: Yes No Surgeon: Yes No<br>Any other:	
5	Any other specific observations/ comments	

E. Radiology Department

(Please document the information about the Radiology Department.)

Slno	Item/ Question	Response
1	Total number of Ultrasound machines	
1.1	How may Ultrasound machines have option for digital image saving?	
1.2	Is the digital image saving a routine practice? (Yes/ No)	
1.3	If Yes, for how many days usually the digital images are saved?	
2	Total number of X-ray machines (for barium enema/air contrast enema)	
3	Number of fluoroscopy machine (for barium enema/air contrast enema)	
4	Number of faculty/ specialists	
5	Number of senior residents	
6	Number of academic junior residents/ DNB students	
7	What is the usual timing for ultrasound in the hospital?	From<br>To

For peer review only

8	What is the arrangement for doing ultrasound beyond routine hours/ nights?	
9	Does the machine(s) used for ultrasound in emergency have facility for digital image saving?	
10	How the reports of ultrasound are given to patients?	
10.1	Only paper report (no film/ image)	
10.2	Paper report with printed paper image (no films)	
10.3	Paper report with films	
10.4	Paper report with CD with digital images	
10.5	Paper report and others available on request/ payment	
10.6	How the reports of barium enema are given to patients?	
10.7	Only paper report (no film/ image)	
10.8	Paper report with films	
10.9	Paper report with CD with digital images	
10.10	Paper report and others (film/CD) on request/ payment	
11	How many patients aged < 5 years with of intussusception were seen in last one year in this department? (approximate number)	
12	How many infants (< 1 years) with of intussusception were seen in last one year in this department? (approximate number)	
13	Do you manage the infants/ children with intussusception by barium/ air contrast enema? (Yes/ No)	
14	If Yes, when was the last such case managed?	
15	Does the department keep a copy of the ultrasound report for record? (Yes, No)	
16	If Yes, where is the file/ archival is made?	
17	Does the radiology department maintain a register/ listing of patients undergoing investigation (ultrasound/ barium)?	
18	What all information can be retrieved from the list/recording?	
18.1	Admission number	
18.2	Date of investigation	
18.3	Name	
18.4	Age	
18.5	Sex	
18.6	Findings/ Diagnosis	
19	Any other specific observation/ comment	

For peer review only - http://bmjopen.bmj.com/site/about/guidelines.xhtml

F. Case Registration System

(Please document about the case registration system of the hospital for in-patient cases)

Sl no	Questions/ Items	Response (Yes/No)
1	What is the system of Central In-patient Admission Registration?	
1.1	Computerized/ electronic admission registration	
1.2	Manual admission registration	
1.3	Any other method (Specify …………………………………………………….)	
2	If the Registration system is manual, how is the Registration Index maintained?	
2.1	Maintained department wise	
2.2	Maintained all departments combined	
2.3	Any other method	
3	Is the Central In-patient Admission Registration system available round the clock?	
4	Is the Emergency Case Registration System separate from the Central in-patient registration?	
5	If Yes, What is the system of Emergency Registration System?	
5.1	Computerized/ electronic admission registration	
5.2	Manual admission registration	
5.3	Any other method (Specify …………………………………………………….)	
6	What all information can be retrieved from the Central In-patient Admission Registration Information? (Yes/No)	
6.1	Admission number	
6.2	Date of admission	
6.3	Department of admission	
6.4	Name	
6.5	Age	
6.6	Sex	
6.7	Diagnosis at admission	
6.8	Final diagnosis	
6.9	Outcome (discharge/ death/LAMA)	
6.10	Date of outcome	
7	Any specific observation/ comments	

For peer review only - http://bmjopen.bmj.com/site/about/guidelines.xhtml

G. Medical Record Archival System

The project requires screening/review of the hospitalized infants in last five years. Thus listing and retrieval of medical records are very essential. The following questions/items focus on the admission, medical record archival and retrieval process. This will allow planning the case record tracking and retrieval at the institution.

Sl no	Questions/ Items	Response (Yes/No)
1	Does your hospital have a Medical records Department/ Division?	
2	How frequently the in-patient case-sheets (hard copies) are sent to Medical Records Department/ Division from the departments?	
3	How the in-patient case-sheets (hard copies) are stored/ archived in your hospital/institution?	
3.1	Admission number wise	
3.2	Department wise	
3.3	Date of admission wise	
3.4	Date of discharge wise	
3.5	Any other method of organization (specify…………………………………………………)	
4	How are the in-patient case-sheets (hard copies) indexed/ listed?	
4.1	Computerized/ electronic listing	
4.2	Manual listing	
4.3	Any other method (specify………………………………………………………..)	
4.4	Not listed	
5	If computerized/ electronic indexing/ listing is done, since when (year) it is being done?	Year
6	Does the institute summarize the medical records of hospitalized patients periodically according to any classification system (ICD 10 or any other)?	
7	If yes, who does the classification labeling of the cases?	
8	How frequently the classification labeling of the cases is done?	
9	Is there any policy by the hospital regarding duration of archival/ storage of the in-patient case-sheets (hard copies)?	
10	If Yes, for how many years the hard copies are stored?	

For peer review only

11	Is there any effort for digital storage of the in-patient case-sheets?	
12	If yes, since when? (year)	
13	What is the process of retrieval of the case records from the Medical Records Department? (please describe focusing on the following)<br>a. The process of application:<br>b. Time needed for approval<br>c. Time needed for retrieval and receipt of the case records<br>d. Any limitation on the number of case records issued in one go	
14	How can we retrieve the list of children admitted with diarrhea to the hospital in month of June 2010? (please ask the concerned official for demonstrating the same)	
15	If retrieved, please document the number of children admitted with diarrhea in the month of June 2010.	
16	Any other challenge in retrieval of the in-patient case-sheets (hard copies) for last five years for the pediatrics and pediatric surgery departments?	
17	Any specific observation/ comments	

For peer review only

H. Institute authority and regulatory approvals

The project requires retrieval of the case-sheets of children with intussusception admitted to your hospital and collection of clinical details about them. Please inform about the approvals from the institute authority(ies) to be obtained for the same.

Sl no	Questions/ Items	
**1**	**What all permissions from institute/department authorities are needed and the time needed for the same?**	
	Authority	Time needed
1.1		
1.2		
1.3		
1.4		
1.5		
**2**	**How frequently (in months) the institute ethics committee meets?**	
**3**	**Meeting with Department Head and Faculty Colleagues**<br>**What is your assessment about the reputation of the potential investigator and support anticipated from the department?**	
**4**	**Meeting with Institute Head or Research Head**<br>**What is your assessment about the research environment in the institute and facilitations anticipated from the department?**	
**5**	**What all challenges do you anticipate in this process?**	

For peer review only

**6**	**How all these challenges can be addressed/mitigated?**
**7**	**Any other specific comments/ suggestions?**

I. Investigating Team Members

Please inform about the Investigating team members from the institute along with their affiliations and contact information.

	Department	Name	Designation	Address details (including email and phone)
1	Pediatrics			
2	Pediatric Surgery			
3	Radiology			
4	Medical Records			
5	Any other			

We thank you for your support and time taken to complete the required information.

Any other observations/ comments

Signature Name

Date: ……/……../…………………..

For peer review only - http://bmjopen.bmj.com/site/about/guidelines.xhtml

For peer review only

BMJ Open

Experience of establishing and coordinating a nationwide network for bi-directional intussusception surveillance in India: Lessons for multisite research studies

Journal:	*BMJ Open*
Manuscript ID	bmjopen-2020-046827.R1
Article Type:	Original research
Date Submitted by the Author:	01-Apr-2021
Complete List of Authors:	Study Group, The INCLEN Intussusception Surveillance; The INCLEN Trust International<br>Das, Manoja; The INCLEN Trust International
**Primary Subject Heading**:	Public health
Secondary Subject Heading:	Global health, Paediatrics
Keywords:	PUBLIC HEALTH, Community child health < PAEDIATRICS, EPIDEMIOLOGY, Paediatric gastroenterology < PAEDIATRICS

SCHOLARONE™
Manuscripts

I, the Submitting Author has the right to grant and does grant on behalf of all authors of the Work (as defined in the below author licence), an exclusive licence and/or a non-exclusive licence for contributions from authors who are: i) UK Crown employees; ii) where BMJ has agreed a CC-BY licence shall apply, and/or iii) in accordance with the terms applicable for US Federal Government officers or employees acting as part of their official duties; on a worldwide, perpetual, irrevocable, royalty-free basis to BMJ Publishing Group Ltd ("BMJ") its licensees and where the relevant Journal is co-owned by BMJ to the co-owners of the Journal, to publish the Work in this journal and any other BMJ products and to exploit all rights, as set out in our licence.

The Submitting Author accepts and understands that any supply made under these terms is made by BMJ to the Submitting Author unless you are acting as an employee on behalf of your employer or a postgraduate student of an affiliated institution which is paying any applicable article publishing charge ("APC") for Open Access articles. Where the Submitting Author wishes to make the Work available on an Open Access basis (and intends to pay the relevant APC), the terms of reuse of such Open Access shall be governed by a Creative Commons licence – details of these licences and which Creative Commons licence will apply to this Work are set out in our licence referred to above.

Other than as permitted in any relevant BMJ Author's Self Archiving Policies, I confirm this Work has not been accepted for publication elsewhere, is not being considered for publication elsewhere and does not duplicate material already published. I confirm all authors consent to publication of this Work and authorise the granting of this licence.

For peer review only

Original article

Title: Experience of establishing and coordinating a nationwide network for bi-directional intussusception surveillance in India: Lessons for multisite research studies

Short title: Experience from multisite intussusception surveillance network in India

Author Information

The INCLEN Intussusception Surveillance Network Study Group

The INCLEN Trust International, Okhla Industrial Area, Phase 1, New Delhi -110020, India

E-mail: drmanojkdas@gmail.com

Corresponding Author

Manoja Kumar Das

Director Projects, The INCLEN Trust International, F1/5, Okhla Industrial Area, Phase 1, New Delhi -110020, INDIA

Phone: Off: +91-11-47730000; Mob: +91-9810203768; Fax: +91-11-47730001

E-mail: manoj@inclentrust.org

Word count: Abstract- 292 words; Main document- 3143 words

For peer review only

For peer review only - http://bmjopen.bmj.com/site/about/guidelines.xhtml

Author information

'The INCLEN Intussusception Surveillance Network Study Group' list of authors

1. Manoja Kumar Das

   Director Projects, The INCLEN Trust International, New Delhi, India

   Email: manoj@inclentrust.org

2. Narendra Kumar Arora

   Executive Director, The INCLEN Trust International, New Delhi, India

   Email: nkarora@inclentrust.org

3. Arindam Ray

   Senior Program Officer, Bill and Melinda Gates Foundation, India Country Office, New Delhi, India

   Email: arindam.ray@gatesfoundation.org

4. Ashish Wakhlu

   Professor, Department of Paediatric Surgery, King George's Medical University, Lucknow, Uttar Pradesh, India

   Email: ashish_wakhlu@hotmail.com

5. Bhadresh R Vyas

   Professor, Department of Paediatrics, MP Shah Government Medical College, Jamnagar, Gujarat, India

   Email: bhadreshrvyas@yahoo.co.uk

6. Javeed Iqbal Bhat

   Assistant Professor, Department of Paediatrics, Sher-I-Kashmir Institute of Medical Sciences, Srinagar, Jammu & Kashmir, India

   Email: drjaveediqbal@gmail.com

7. Jayanta K. Goswami

For peer review only

For peer review only - http://bmjopen.bmj.com/site/about/guidelines.xhtml

Professor, Department of Paediatric Surgery, Gauhati Medical College, Guwahati, Assam, India

Email: jayantagoswami@hotmail.com

8. John Mathai

Professor, Department of Paediatrics, PSG Institute of Medical Sciences, Coimbatore, Tamil Nadu, India

Email: psg_peds@yahoo.com

9. Kameswari K.

Professor, Department of Paediatric Surgery, Andhra Medical College, Vishakhapatnam, Andhra Pradesh, India

Email: kameswari1956@gmail.com

10. Lalit Bharadia

Consultant Paediatric Gastroenterologist, Fortis Escorts Hospital, Jaipur, Rajasthan, India

Email: lalitbharadia@gmail.com

11. Lalit Sankhe

Assistant Professor, Department of Community Medicine, Grant Medical College & JJ Hospital, Mumbai, Maharashtra, India

Email: sankhelalit@yahoo.com

12. Ajaya Kumar M.K.

Professor, Department of Paediatric Surgery, Government Medical College & SAT Hospital, Thiruvananthapuram, Kerala, India

Email: drajaykumarmk@gmail.com

13. Neelam Mohan

Consultant Paediatrics Gastroenterology, Medanta—The Medicity, Gurgaon, Haryana, India

For peer review only

Email: drneelam@yahoo.com

14. Pradeep K. Jena

Professor, Department of Paediatric Surgery, SCB Medical College, Cuttack, Odisha, India

Email: drpkjena@gmail.com

15. Rachita Sarangi

Professor, Department of Paediatrics, IMS & SUM Medical College & Hospital, Bhubaneswar, Odisha, India

Email: rachitapaedia@gmail.com

16. Rashmi Shad

Consultant Paediatrics, Choithram Hospital and Research Centre, Indore, Madhya Pradesh, India

Email: drrashmishad@yahoo.com

17. Sanjib K. Debbarma

Associate Professor, Department of Paediatrics, Agartala Government Medical College, Agartala, Tripura, India

Email: dr_sanjibdb@rediffmail.com

18. Shyamala J.

Consultant Paediatrics, Apollo Hospitals, Chennai, Tamil Nadu

Email: jaymoorthy@hotmail.com

19. Simmi K. Ratan

Professor, Department of Paediatric Surgery, Maulana Azad Medical College, Delhi, India

Email: drjohnsimmi@yahoo.com

20. Suman Sarkar

Assistant Professor, Department of Paediatrics, Institute of Post Graduate Medical Education and Research, Kolkata, West Bengal, India

For peer review only

For peer review only - http://bmjopen.bmj.com/site/about/guidelines.xhtml

Email: dr.sumansarkar@gmail.com

21. Vijayendra Kumar

Professor, Department of Paediatric Surgery, Indira Gandhi Institute of Medical Sciences, Patna, Bihar, India

Email: drvijayendrakr@rediffmail.com

22. Anand P. Dubey

Professor, Department of Paediatrics, Maulana Azad Medical College, Delhi, India

Email: Anand_dubey52@hotmail.com

23. Atul Gupta

Consultant Paediatric Surgery, Fortis Escorts Hospital, Jaipur, Rajasthan, India

Email: atulgupta26878@gmail.com

24. Bikasha Bihary Tripathy

Associate Professor, Department of Paediatric Surgery, IMS & SUM Medical College & Hospital, Bhubaneswar, Odisha, India

Email: bbtripathy.dr@gmail.com

25. Cenita J. Sam

Professor, Department of Paediatric Surgery, PSG Institute of Medical Sciences, Coimbatore, Tamil Nadu, India

Email: cenitasivamani@yahoo.com

26. Gowhar Nazir Mufti

Assistant Professor, Department of Paediatric Surgery, Sher-I-Kashmir Institute of Medical Sciences, Srinagar, Jammu & Kashmir, India

Email: gowharmufti@yahoo.co.in

27. Harsh Trivedi

For peer review only

Professor, Department of Paediatric Surgery, MP Shah Government Medical College, Jamnagar, Gujarat, India

Email: trivediharsh@indiatimes.com

28. Jimmy Shad

Consultant Paediatric Surgery, Apollo Hospitals, Chennai, Tamil Nadu, India

Email: shadjimmy@yahoo.co.in

29. Kaushik Lahiri

Consultant, Department of Paediatric Surgery, Gauhati Medical College, Guwahati, Assam, India

Email: Kaushik_1671979@rediffmail.com

30. Meera Luthra

Consultant Paediatric Surgery, Medanta- The Medicity, Gurgaon, Haryana, India

Email: meera.luthra@medanta.org

31. Padmalatha P

Professor, Department of Paediatrics, Andhra Medical College, Vishakhapatnam, Andhra Pradesh, India

Email: padmap_2000@yahoo.com

32. Rakesh Kumar

Associate Professor, Department of Paediatrics, Indira Gandhi Institute of Medical Sciences, Patna, Bihar, India

Email: drjaiswalrakesh@gmail.com

33. Ruchirendu Sarkar

Professor, Department of Paediatric Surgery, Institute of Post Graduate Medical Education and Research, Kolkata, West Bengal, India

Email: ruchirendu@gmail.com

For peer review only

For peer review only - http://bmjopen.bmj.com/site/about/guidelines.xhtml

34. Santosh Kumar A.

Professor, Department of Paediatrics, Government Medical College & SAT Hospital, Thiruvananthapuram, Kerala, India

Email: hod.pediatrics.sath@gmail.com

35. Subrat Kumar Sahoo

Associate Professor, Department of Paediatric Surgery, IMS & SUM Medical College & Hospital, Bhubaneswar, Odisha, India

Email: dr_subratsahoo@yahoo.com

36. Sunil K. Ghosh

Associate Professor, Department of Pediatric Surgery, Agartala Government Medical College, Agartala, Tripura, India

Email: sunilkrghosh752@gmail.com

37. Sushant Mane

Assistant Professor, Department of Paediatrics, Grant Medical College & JJ Hospital, Mumbai, Maharashtra, India

Email: drsush2006@gmail.com

38. Bashir Ahmad Charoo

Professor, Department of Paediatrics, Sher-I-Kashmir Institute of Medical Sciences, Srinagar, Jammu & Kashmir, India

Email: charoobash@gmail.com

39. Rajendra Prasad G.

Professor, Department of Paediatric Surgery, Andhra Medical College, Vishakhapatnam, Andhra Pradesh, India

Email: dr.grajendraprasad@gmail.com

40. Harish Kumar S

For peer review only

Paediatrics Radiologist, Apollo Hospitals, Chennai, Tamil Nadu, India

Email: drharishkumar.s@gmail.com

41. Jothilakshmi K.

Professor, Department of Paediatrics, PSG Institute of Medical Sciences, Coimbatore, Tamil Nadu, India

Email: drjothi.cbe@gmail.com

42. Nihar Ranjan Sarkar

Associate Professor, Department of Radiology, Institute of Post Graduate Medical Education and Research, Kolkata, West Bengal, India

Email: dr.niharsarkar@gmail.com

43. Pavai Arunachalam

Professor, Department of Paediatric Surgery, PSG Institute of Medical Sciences, Coimbatore, Tamil Nadu, India

Email: pavai4321@yahoo.com

44. Satya S.G. Mohapatra

Professor, Department of Radiology, IMS & SUM Medical College & Hospital, Bhubaneswar, Odisha, India

Email: drartifact@gmail.com

45. Saurabh Garge

Consultant Paediatric Surgery, Choithram Hospital and Research Centre, Indore, Madhya Pradesh, India

Email: saurabhgarge8@gmail.com

For peer review only

Abstract

Introduction: Multisite studies experience several challenges involving patient recruitment, protocol adherence, data quality and coordination related challenges. Several of these challenges are results of poor site selection and quality assurance measures. There is limited documentation of these processes and guidelines for conduct of multisite studies in developing countries like India.

Material and methods: Linked to the rotavirus vaccine introduction in India, a multisite surveillance network was established to document the intussusception epidemiology in children. The study sites were selected through a systematic and objective four-step selection process. The multilayer quality assurance measures were adopted to document protocol adherence and data collection quality.

Results: The site selection process involved four steps including shortlisting of potential institutions, information seeking and telephonic interaction, site visits and site selection using objective criteria. Out of over 400 hospitals screened across India, 40 potential institutions were shortlisted. Information was sought from these institutes by questionnaire and interaction with investigators done. Following visits to 25 institutes, 19 sites were selected to participate in the study. The multi-step selection process allowed filtering and identification of sites with adequate capacity and motivated investigators. The multilayer quality assurance measures monitored and ensured protocol adherence, complete record retrieval and data completeness. The key challenges experienced included time taken for obtaining regulatory and ethical approvals, which delayed completion of the study. Ten sites continued with another multisite vaccine safety surveillance study.

Conclusion: The experience and results of this systematic and objective site selection method in India are promising. The systematic multi-step site selection and data quality assurance methods presented here are feasible and practical. The lessons from the establishment and

coordination of this surveillance network can be useful in planning, selecting the sites and conducting multisite and surveillance studies in India and developing countries.

Keywords: Surveillance, Multisite study, Site selection, Quality assurance, Coordination, Challenges, Intussusception, India

For peer review only

For peer review only - http://bmjopen.bmj.com/site/about/guidelines.xhtml

Article summary

Strengths and limitations of this study

- Provides a systematic study site selection process using objective methodology
- Documents the experience with prospective and retrospective surveillance data collection with multi-layered data quality assurance process
- The study site selection and quality assurance measures may serve as reference for suitable adaptation in different research topics and contexts
- The lessons are applicable only to the multisite studies.
- The documentation of contractual and financial management challenges may be limited.

For peer review only

Introduction

232 Introduction
233 Intussusception is an acute emergency in children and most commonly occurs in infants aged 4-10 months (1)(2). Although some are transient and resolve spontaneously, if not intervened timely, it may lead to bowel ischemia and perforation and may even be fatal (1). Intussusception has been reported as an adverse reaction with rotavirus vaccines (RVV) with variable risks ranging from no increased risk to low risk (1-2 additional cases per 100,000 vaccinated infants) across different countries with different RVVs (3)(4)(5)(6)(7)(8)(9)(10)(11)(12). The intussusception background rate varies widely across different countries, 9 (Bangladesh) to 328 (South Korea) per 100,000 infants (13). Limited information from India reported its incidence between 17.7 to 254 cases per 100,000 child-years (14)(15). Several other studies were single or few centre studies and varied in case definitions, age groups, reference periods, and methodology (12)(13)(14).

In view of the vaccine safety concern and limited information on intussusception in India, the National Technical Advisory Group on Immunization recommended vaccine safety surveillance along with RVV introduction (16). This surveillance network aimed to generate background information on intussusception epidemiology Indian children to inform the policy and program related to RVV introduction and serve as baseline for future surveillance to identify any change after vaccine introduction and address the vaccine safety concerns. The objectives were to: (1) establish a surveillance network of public and private hospitals ensuring regional representation and data capturing system; (2) undertake retrospective surveillance to document the intussusception epidemiology over past five years; (3) undertake prospective surveillance to document the intussusception epidemiology over 18 months and potential linkage with RVVs; and (4) build capacity of the investigators and institutions.

Multicentre studies face several challenges related to design, site selection, regulatory approvals, study conduct, coordination, data management and dissemination (17)(18)(19)(20).

For peer review only - http://bmjopen.bmj.com/site/about/guidelines.xhtml

The lessons from multisite studies in India are limited. The intussusception surveillance study has been successfully completed and the network has evolved over time to undertake more vaccine safety studies. The purpose of this paper is to share the process of network establishment, coordination, data management and lessons learned from such nationwide network research.

Methods

Study design: This study combined both retrospective and prospective surveillance. The experiences and lessons presented here are based on the concurrent documentation of processes and retrospective review of the study documents.

Study governance: The Central Coordinating Unit (CCU) constituted investigators, research staffs and administrative and finance team. For technical integrity and implementation monitoring, a Technical Advisory Group (TAG) was constituted with 17 Indian and international experts in vaccine safety, surveillance, immunization program, public health, child health, paediatric surgery, radiology and medical record system, representation from Ministry of Health and Indian Council of Medical Research.

Site selection: We followed a four-step systematic study site selection process (Figure 1). *Step 1 (Shortlisting):* We categorised the states into four regions (north, south, east and west) and screened the tertiary care hospitals (medical colleges and private hospitals) in these regions from the websites. *Step 2 (Screening):* We solicited information about the case load, clinical and diagnostic capacities (paediatrics, paediatric surgery, and radiology), medical record system, ethical and administrative approvals from the shortlisted institutions using questionnaire and telephonic interactions (Supplementary document 1). *Step 3 (Evaluation):* A TAG member visited the institutions to assess the institution capacity (clinical facility, case documentation, research support system, medical record-keeping system) and interacted with

For peer review only - http://bmjopen.bmj.com/site/about/guidelines.xhtml

the potential investigator(s), department and institution leaderships to assess the commitment, institution support and research environment (Supplementary document 2). *Step 4 (Site finalisation):* Based on the questionnaire, interaction with investigators and TAG member feedback, the study sites were finalised.

Protocol finalisation and study tool development: In multisite research projects, consensus building among the investigators on the study protocol, case record forms (CRFs), data management and monitoring are critical. The study protocol and CRFs were shared and piloted at the study sites. The protocol, study tools, implementation, data management and monitoring were discussed in detail during protocol finalisation workshop involving lead investigators from all sites and TAG members.

Regulatory procedures: As this study involved international funding, the approval from Health Ministry Screening Committee (HMSC) was obtained. The study protocol documents were submitted to the participating institutes for ethical approvals. Following the ethical approval, the study was implemented at the sites.

Study implementation process: Before implementation of the study at the sites, the following four items had to be completed: (1) institute administrative approval; (2) ethical approval; (3) agreement executed with the institution; and (4) research staff selected and trained.

Data collection, management and monitoring: Separate CRFs and log sheets were prepared for the retrospective and prospective data collection. The retrospective data collection involved multiple sources; case records, registers from different departments and operation theatres and medical records section. For case record retrieval, the sites following International Classification of Diseases (ICD, ICD-9 or ICD-10) system, the ICD codes for intussusception or acute abdomen conditions were used (Supplementary document 3) (published in methodology paper) (21). At the other sites, cases were identified as per the diagnoses. For the prospective data collection, all admitted children were screened to identify the suspected cases,

For peer review only - http://bmjopen.bmj.com/site/about/guidelines.xhtml

who were followed till final diagnosis. The children with intussusception were recruited and data collected. A weekly reporting from the sites was solicited for tracking progress. Initially a weekly call with the study teams and later fortnightly to monthly calls were done to monitor the progress and address challenges. Based on the data collection progress reports, a monthly bulletin was prepared indicating the progresses and data quality. The completed study tools were sent periodically to CCU. The data received at CCU was reviewed for completeness, correctness and queries were resolved with reference to the source documents. Double data entry was done using customised data entry platform with inbuilt data matching program. The data was archived in the server with authorised access and regular backup. Data analysis was done by under guidance of TAG. The data collection, flow and management followed for prospective and retrospective surveillance are shown in figure 2 and figure 3.

Quality assurance measures: Multiple quality assurance measures focused on protocol adherence and data quality. After initiation of data collection, TAG members visited each study site during the retrospective surveillance to verify: (a) protocol adherence; (b) identification of suitable cases; and (c) data abstraction. The corrections/clarifications and repeat training was imparted as per TAG observations. After six months, during the prospective surveillance, TAG member again visited to review the: (a) patient screening; (b) tracking and eligible patients identification; (c) obtaining consent; and (d) data abstraction.

Patient and Public Involvement: No patients or the public were involved in the design, conduct, reporting, and dissemination of research.

For peer review only

Results

Study timelines: The study was sanctioned in November 2014. The HMSC application was submitted in December 2014 and approved in May 2015. The formal study site selection initiated after the HMSC approval and was completed by August 2015. The protocol finalisation workshop was held in October 2015. Following the ethical approvals, the retrospective surveillance was initiated at majority of the sites in February 2016. The prospective surveillance was initiated in April 2016 and continued through September 2017. The data entry, cleaning and analysis and report drafting were completed by June 2018.

Study site selection: Out of over 400 hospitals screened across regions, 40 potential hospitals/institutions (10 per region) were shortlisted. While preparing the database, many institutions didn't have adequate information on their websites about the facilities and faculty members or specialists. The potential investigators from these 40 shortlisted institutions were invited to submit desired information using a questionnaire via email and all responded. Telephonic discussions was held with the potential investigator(s) from these institutions (45-60 minutes) for additional information or clarifications. Based on the criteria and consultation with TAG, 25 institutes were visited. Based on the questionnaire, interactions and TAG member assessment, 19 institutions (north region- 5 sites, 3 public and 2 private; south region-5 sites, 2 public and 3 private; east region- 6 sites, 5 public and 1 private; west region- 3 sites, 2 public and 1 private) were selected (figure 4). The study originally planned for 17 sites. Two more sites from the states where RVV was scheduled for introduction were added later. Several potential investigators requested the CCU team to discuss with their institute leadership for permission and allow him/her to lead the project. The CCU team succeeded in all expect two institutions, which couldn't be included. The site selection process was delayed by seven months due to the delay in HMSC approval.

For peer review only - http://bmjopen.bmj.com/site/about/guidelines.xhtml

Regulatory approvals: The ethics approvals from the sites needed average 4 months (range: 1 to 8 months). No protocol amendment was needed.

Data collection: During July 2010 and March 2016 (retrospective surveillance period), out of 42,866 admitted under-two years children, 2092 suspected cases were identified and 1588 confirmed intussusception cases were recruited (22). The cases recruited at study sites ranged from 14 to 652. While five sites documented <20 cases each, two sites contributed >100 cases each. During April 2016 and September 2017 (prospective surveillance period), out of 6300 hospitalised under-two years children, 1203 suspected cases were identified and 621 confirmed intussusception cases were recruited (23). The cases recruited at the study sites ranged from 5 to 191 cases. While seven sites recruited <10 cases each, three sites contributed >50 cases each. At one study site, very few cases were retrieved from the retrospective and prospective surveillance, contrary to the anticipation. The descriptive data analysis was done at pooled and regional levels to document the epidemiology of intussusception. The variables and data parameters between the regions were compared to identify the similarities and differences.

Medical record retrieval: Twelve (seven private and five public) institutions had electronic medical record (EMR) system. The ICD system was used at all sites except the two private hospitals, where the diagnosis was used for listing and archival. The medical records were accessible at all study sites except one site, where the research staff faced challenge in timely and adequate access to the medical records. The medical records required organisation at this site and thus the retrospective data collection took longer. The record retrieval was quicker at institutions with EMR system and retrospective data collection was completed in 2-4 months. At two sites the retrospective data collection took longer 6 and 10 months, due to higher case load and challenge in record retrieval, respectively.

Radiology documentation: The ultrasound digital images and report were available for all prospectively recruited cases. There were variations in the ultrasound finding documentations

For peer review only - http://bmjopen.bmj.com/site/about/guidelines.xhtml

and details in the reports, which required clarification from the radiologist for data capturing. Ultrasound reports were available at all but one hospital, where the findings were recorded in the case sheet. Digital records of ultrasounds were stored for minimum three months to five years. These ultrasound reports, digital films and clinical records were reviewed by independent TAG members for confirmation and classification according to Brighton Collaboration Criteria (1).

Vaccination information: Majority of the parents were not carrying the vaccination card at the time of hospitalisation and about half of them came from outside the city. The research staffs pursued to obtain the vaccination card during hospitalisation and after discharge. The vaccination card after discharge was obtained through email, mobile message and also self-addressed and stamped envelopes handed over to the parents. The vaccination information was available for 78.4% of the prospective cases.

Clinical case management: At five sites only surgery was done. At two sites reduction was available during daytime only and surgery during other period and at twelve sites both methods were available always.

Data quality assurance: TAG members made two quality assurance visits, first during the retrospective surveillance period, second during the prospective surveillance period and provided written feedback. The retrospective surveillance was at risk of missing cases. As it was difficult to compare the absolute case numbers across the centres and periods, we derived intussusception case rate per 1000 paediatric surgery admissions. After completion of one year of prospective surveillance, third quality assurance visit was made by the data management team to 11 sites with >30% differences in the case rates between retrospective and prospective periods. The team targeted verifying the confirmed cases, suspected cases and total admissions during 2015, 2016, and 2017 (until visit). The team identified three missed cases (one each at three sites).

For peer review only - http://bmjopen.bmj.com/site/about/guidelines.xhtml

Investigators commitment and transition: Despite the systematic selection process, the involvement of the investigators in the day-to-day operations and data verification was lesser than expected at three sites. At three sites the lead investigators transitioned without any impact on the study activities.

Research staff issues: Although one research staff per site was planned, at seven sites two research staffs were engaged considering the efforts needed for data retrieval and case load. These research staffs were trained through regional training workshops followed by hands-on training. At eight sites the research staffs changed during the study period, once at six sites and twice at two sites. All the new staffs were trained by the CCU team member through site visit followed by virtual support. The investigators swiftly ensured replacements and no loss of cases. At CCU, there were three rounds of research staff transition.

Intra-and inter-department coordination: At all institutions the nurses, residents and faculty members supported the surveillance. The nurse and residents at most places informed the research staff about any confirmed case, even on holidays. If the patient was discharged on any holiday, the research staffs attended and collected the data. Intra- and inter-departmental coordination for data collection were smooth at all sites except two sites, where the participation from paediatric department was limited. As most of the intussusception cases were directly admitted to or transferred to paediatric surgery department on diagnosis, it didn't affect the case screening and recruitment.

Financial management: In view of the foreign funding source and Foreign Contribution Regulation Act (FCRA) regulation, the funds couldn't be transferred to most institutes without FCRA approval. Thus, the fund was managed by the CCU for most of the institutes. Despite no fund transferred to the institutes, agreements were executed between the institutes with the investigators as the witnesses. We experienced challenges with some institutes in explaining the FCRA obligations prior to agreement execution.

For peer review only - http://bmjopen.bmj.com/site/about/guidelines.xhtml

Study tenure: Although planned for 30 months, the study was completed in 43 months. The delays in HMSC and ethics approval at sites were the key reasons for the delay.

Dissemination: Four peer-reviewed manuscript have been published and eight manuscripts are under review or in preparation. The manuscripts have all the site investigators as authors, either individually or as group. The findings have been shared with the Ministry of Health and Family Welfare, World Health Organisation and other key partners. Over 22 oral presentations had been made at national, regional and international meetings.

Capacity building and transition: After this study, several sites were included in other studies coordinated by the CCU, ten sites in one study and four sites in another study. Four site investigators collaborated in other research projects. Several of the research staffs at these sites continued with the new studies.

Discussion

This multicentre sentinel surveillance successfully generated clinical and sociodemographic epidemiology information on intussusception in children including the regional the variations. The network adopted a systematic site selection process. A common understanding among the investigators was ensured and transmitted to the research staffs for appropriate implementation. The effective coordination, data management and quality assurance measures ensured high quality data. Despite the efforts there were challenges related to the regulatory and administrative challenges, which affected the implementation timelines. The continuation of several sites and investigators in other studied demonstrated the strong collaboration and confidence coupled with capacity building.

Poor site selection can lead to delay in completion, rise in cost, protocol amendment, implementation variations or study failure (24). Studies have used different site selection processes: active hunting, peer referrals, inviting expression of interests, engaging research

For peer review only - http://bmjopen.bmj.com/site/about/guidelines.xhtml

organisations, etc. (25)(26)(27). Our organisation has been conducting multisite network studies (ranging from 5 to 84 partner institutions) over last 15 years. We have adopted variable combination of processes for the study site and investigators selection, which have evolved over time. For this study, we adopted a systematic site selection process, which appeared lengthy and required several rounds of interactions. The four-step process assisted in identifying the highly motivated investigators and study sites with desired capacity. The potential investigators who failed to return the questionnaire with desired information or participate in the interaction are unlikely to devote time for study conduct and supervision. The systematic selection process allowed reducing the potential sites to a manageable number of suitable sites that were further assessed through visit. While the process was lengthy, it allowed also to build common understanding and commitment for the study. These experiences were similar to some multicounty studies (25)(28)(29).

The procedural efforts and time taken for ethical approval from the site institutes are well known. A review observed that the study site ethics approval took from 5 to 798 days and the review process and contents varied widely (30). The ethics review process consumed sizable staff hours and budget, forcing timeline extension and budget shortfall (30)(31)(32). In our study also the documentation and formats used for submission varied widely, apart from the study protocol, CRFs and consent forms. Some of the committees asked the investigators to present their protocol, while others didn't.

Although the investigators were responsible for the conduct of the study, the agreements with institutions assisted in implementation of the study. The involvement of external experts TAG as in study site selection and monitoring facilitated standardisation and quality assurance. The additional data retrieval and verification effort at the sites documented the protocol adherence and robustness of data collection.

For peer review only - http://bmjopen.bmj.com/site/about/guidelines.xhtml

This is the first documentation of systematic site selection process for a multisite network in India. The positive experience from this study encouraged adoption of similar systematic site selection and data quality assurance mechanism for subsequent two vaccine network studies in India.

There are some limitations in the current study and documentation. While many of the lessons may be generic and have relevance for most of the multisite studies, some of them may be relevant for India and the developing countries. The documentation of contractual and financial management challenges may be limited. The study was not a clinical trial and didn't involve any laboratory procedures.

Conclusions

In conclusion, our experience with this systematic study site selection process was positive and satisfying. It used a four-step selection process using questionnaire, objective criteria for assessment, and interactions with the investigators and institution stakeholders with site visits to identify suitable sites with adequate capacity. The participation of motivated instigators, agreement with the institutions and contributory protocol and tool development facilitated successful completion. Difficulties and delays in site initiation were primarily due to the regulatory and administrative approvals. The multistep site selection adopted is feasible and practical even in Indian context. The quality assurance processes also assisted in high quality data collection. We hope that the site selection and quality assurance processes would be informative for multisite studies in India and developing countries.

For peer review only - http://bmjopen.bmj.com/site/about/guidelines.xhtml

Abbreviations

BCDC Brighton collaboration diagnostic criteria

CCU Central coordinating unit

CRF Case record form

EMR Electronic medical record

FCRA Foreign Contribution Regulation Act

HMSC Health Ministry Screening Committee

ICD International Classification of Diseases

RVV Rotavirus vaccines

TAG Technical Advisory Group

Usg Ultrasound

For peer review only

Acknowledgments

We acknowledge the support from Ministry of Health and Family Welfare, Government of India for undertaking the study. We are thankful to the hospital administrations and the clinicians at the study site institutes, who supported and facilitated undertaking of the study.

We highly value the technical guidance and inputs provided by the members of Technical Advisory Group: Satinder Aneja, Anju Seth and Archana Puri, Lady Hardinge Medical College, New Delhi; Ashok Patwari, Hamdard Institute of Medical Sciences & Research, New Delhi; Yogesh Kumar Sarin, Maulana Azad Medical College, New Delhi; Rakesh Aggarwal, Anshu Srivastava and Ujjal Poddar, Sanjay Gandhi Postgraduate Institute of Medical Sciences, Lucknow; Malathi Satyasekharan, Kanchi Kamakoti Chailds Trust Hospital, Chennai; Raju Sharma and Nirupam Madan, All India Institute of Medical Sciences, New Delhi; Jyoti Joshi and Deepak Polpakara, Immunization Technical Support Unit; Ministry of Health & Family Welfare, New Delhi; Umesh D. Parashar; Centers of Disease Control and Prevention, Atlanta, USA; Naveen Thacker, Child Health Foundation, Gandhigram; and Rashmi Arora, Indian Council of Medical Research, Ansari Nagar, New Delhi.

We acknowledge the contribution of the research staffs at The INCLEN Trust International: Harshpreet Kaur, Janvi Chaubey, Mrimmaya Das, Shweta Sharma and Vaibhav Jain.

We highly appreciate the efforts made by the research staffs at the study sites: Aarezo Bashir and Rafia; Sher-e-Kashmir Institute of Medical Sciences, Srinagar, Jammu & Kashmir; Prabha Shankar, Medanta-The Medicity Hospital, Gurgaon, Haryana; Anju Sharma; Maulana Azad Medical College, New Delhi; Anita Singh and Shubhranshu Srivastava, King George Medical University, Lucknow, Uttar Pradesh; Hemant Meena, Choithram Hospital, Indore, Madhya Pradesh; Pankaj Kumar and Shashi Kant; Indira Gandhi Institute of Medical Sciences, Patna, Bihar; Goutam Benia, IMS & SUM Medical College & Hospital, Bhubaneshwar, Odisha; Prasntajyoti Mohanty, SVP Post Graduate Institute of Paediatrics, Cuttack, Odisha;

For peer review only

533 Angshuman Chatterjee, Institute of Postgraduate Medical Education and Research & SSKM
534 Hospital, Kolkata, West Bengal; S. Yamuna, Andhra Medical College, Vishakhapatnam,
535 Andhra Pradesh; Srinidhi Sudan, Apollo Hospitals, Hyderabad, Telengana; Rajesh Francis,
536 Apollo Hospitals, Chennai, Tamil Nadu; T. Easter Chandru, PSG Institute of Medical Sciences,
537 Coimbatore, Tamil Nadu; Deepthy R, Julie and Anju Shivkumar, Government Medical College
538 & SAT Hospital, Thiruvananthapuram, Kerala; Archit Vaidya, Grant Medical College & JJ
539 Hospital, Mumbai, Maharashtra; Nimesh Chouksey, MP Shah Government Medical College,
540 Jamnagar, Gujarat; Nidhi Singh, Fortis Escorts Hospital, Jaipur, Rajasthan; Mrinmoy Gohain,
541 Gauhati Medical College, Guwahati, Assam; Arpita Bhattachrjee, Saugat Ghosh and
542 Tanusmita Debnath, Agartala Government Medical College, Agartala, Tripura.

For peer review only

For peer review only - http://bmjopen.bmj.com/site/about/guidelines.xhtml

Declarations

Ethics approval and consent to participate: The study protocol was reviewed and approved by all the participating institutes. The list of ethics committees of the participating institutes include: The INCLEN Independent Ethics Committee, The INCLEN Trust International, New Delhi, India (Ref No: IIEC 23; Dated June 30, 2015); Institutional Ethics Committee, King George's Medical University, Lucknow, Uttar Pradesh, India (Ref no: 7951/Ethics/R.Cell-15; Dated December 4, 2015); Institutional Ethics Committee, MP Shah Government Medical College, Jamnagar, Gujarat, India (ref no: 01/46/2016; Dated January 6, 2016); Institutional Ethics Committee, Sher-I-Kashmir Institute of Medical Sciences, Srinagar, Jammu & Kashmir, India (Ref no: 42/2015; Dated October 29, 2015); Institutional Ethics Committee, Gauhati Medical College, Guwahati, Assam, India (Ref no: MC/02/2015/274; Dated May 30, 2016); Institutional Human Ethics Committee, PSG Institute of Medical Sciences, Coimbatore, Tamil Nadu, India (Ref no 15/294; Dated November 16, 2015); Institutional Ethics Committee, King George Hospital, Andhra Medical College, Vishakhapatnam, Andhra Pradesh, India (Dated October 26, 2015); Institutional Ethics Committee, Fortis Escorts Hospital, Jaipur, Rajasthan, India (Ref no: FEHJ/IEC/15/0023; Dated September 14, 2015); Institutional Ethics Committee, Grant Medical College & JJ Hospital, Mumbai, Maharashtra, India (Ref no: IEC/Pharm/288/15; Dated November 19, 2015); Institutional Ethics Committee, Government Medical College & SAT Hospital, Thiruvananthapuram, Kerala, India (Ref no: 06/05/2015/MCT; Dated December 9, 2015); Medanta Institutional Ethics Committee, Medanta-The Medicity, Gurgaon, Haryana, India (MICR 559/2015; Dated January 21, 2016); Institutional Ethics Committee, SCB Medical College, Cuttack, Odisha, India; Institutional Ethics Committee, IMS & SUM Medical College & Hospital, Bhubaneswar, Odisha, India (Ref no 210/5/10/2015; Dated October 14, 2015); Institutional Ethics Committee; Choithram Hospital and Research Centre, Indore, Madhya Pradesh, India (Ref no: EC/Oct/15/20; Dated

For peer review only

For peer review only - http://bmjopen.bmj.com/site/about/guidelines.xhtml

October 27, 2015); Institutional Ethics Committee, Agartala Government Medical College, Agartala, Tripura, India; Institutional Ethics Committee- Clinical Studies, Apollo Hospitals, Chennai, Tamil Nadu (Dated October 14, 2015); Institutional Ethics Committee, Maulana Azad Medical College, Delhi, India (Ref no: F.1/IEC/MAMC/50/4/2015/308; Dated November 20, 2015); Institutional Ethics Committee, Institute of Post Graduate Medical Education and Research, Kolkata, West Bengal, India (Ref no: Inst/IEC/2016/197; Dated March 1, 2016); Institutional Ethics Committee, Indira Gandhi Institute of Medical Sciences, Patna, Bihar, India (Ref no: 1256/Acad; Dated November 11, 2016); and Ethics Committee, Apollo Hospital, Hyderabad, Telengana, India ((Dated October 13, 2015). The interviews with stakeholders were done after obtaining written informed consent.

Consent for publication: Not applicable

Availability of data and materials: This manuscript reports the processes and experience from the multisite network research. All relevant data has been reported. All data is available with the investigators and can be provided by the corresponding author upon reasonable request.

Competing interests: The authors declare that there is no competing interests and conflict of interest.

Disclosure statement: None. There is no financial interest or benefit for the authors arisen from this project or its direct application.

Funding: This project was supported by the Bill and Melinda Gates Foundation, USA to The INCLEN Trust International (grant number OPP1116433). The funder or its representative had no role in the design of the study and collection, analysis, and interpretation of data and writing the manuscript.

Author contributions:

For peer review only

592 Study conceptualisation, study design, protocol development, training, data analysis,
593 interpretation, coordination, monitoring and quality assurance: MKD and NKA.
594 Participant recruitment and data collection: AW, BRV, JIB, JKG, JM, KK, LB, LS, AMK, NM,
595 PKJ, RS-1, RS-2, SKD, SJ, SKR, SS, VK, APD, AG, BBT, CJS, RPG, GNM, HT, JS, KL,
596 ML, PP, RK, RS-3, SKA, SKS, SKG, SM, BAC, RPG, HKS, JK, NRS, PA, SSGM and SG.
597 Manuscript preparation: MKD, NKA and AR
598 All authors reviewed, provided critical input and approved the final version. The content
599 represents the views of the authors alone and do not necessarily represent the official positions
600 of their organizations.

For peer review only

For peer review only - http://bmjopen.bmj.com/site/about/guidelines.xhtml

References

1. Bines JE, Kohl KS, Forster J, Zanardi LR, Davis RL, Hansen J, et al. Acute intussusception in infants and children as an adverse event following immunization: case definition and guidelines of data collection, analysis, and presentation. Vaccine. 2004 Jan 26;22(5–6):569–74.
2. World Health Organisation. Post-marketing surveillance of rotavirus vaccine safety [Internet]. 2017 [cited 2017 Sep 18]. Available from: http://apps.who.int/iris/bitstream/10665/70017/1/WHO_IVB_09.01_eng.pdf
3. Tate JE, Mwenda JM, Armah G, Jani B, Omore R, Ademe A, et al. Evaluation of Intussusception after Monovalent Rotavirus Vaccination in Africa. N Engl J Med. 2018 Apr 19;378(16):1521–8.
4. Groome MJ, Tate JE, Arnold M, Chitnis M, Cox S, de Vos C, et al. Evaluation of Intussusception After Oral Monovalent Rotavirus Vaccination in South Africa. Clin Infect Dis Off Publ Infect Dis Soc Am. 2020 Apr 10;70(8):1606–12.
5. Patel MM, López-Collada VR, Bulhões MM, De Oliveira LH, Márquez AB, Flannery B, et al. Intussusception Risk and Health Benefits of Rotavirus Vaccination in Mexico and Brazil. N Engl J Med. 2011 Jun 16;364(24):2283–92.
6. Velázquez FR, Colindres RE, Grajales C, Hernández MT, Mercadillo MG, Torres FJ, et al. Postmarketing surveillance of intussusception following mass introduction of the attenuated human rotavirus vaccine in Mexico. Pediatr Infect Dis J. 2012 Jul;31(7):736–44.
7. Carlin JB, Macartney KK, Lee KJ, Quinn HE, Buttery J, Lopert R, et al. Intussusception risk and disease prevention associated with rotavirus vaccines in Australia's National Immunization Program. Clin Infect Dis Off Publ Infect Dis Soc Am. 2013 Nov;57(10):1427–34.

For peer review only - http://bmjopen.bmj.com/site/about/guidelines.xhtml

8. Yih WK, Lieu TA, Kulldorff M, Martin D, McMahill-Walraven CN, Platt R, et al. Intussusception risk after rotavirus vaccination in U.S. infants. N Engl J Med. 2014 Feb 6;370(6):503–12.

9. Weintraub ES, Baggs J, Duffy J, Vellozzi C, Belongia EA, Irving S, et al. Risk of intussusception after monovalent rotavirus vaccination. N Engl J Med. 2014 Feb 6;370(6):513–9.

10. Stowe J, Andrews N, Ladhani S, Miller E. The risk of intussusception following monovalent rotavirus vaccination in England: A self-controlled case-series evaluation. Vaccine. 2016 12;34(32):3684–9.

11. Pérez-Vilar S, Díez-Domingo J, Puig-Barberà J, Gil-Prieto R, Romio S. Intussusception following rotavirus vaccination in the Valencia Region, Spain. Hum Vaccines Immunother. 2015;11(7):1848–52.

12. Koch J, Harder T, von Kries R, Wichmann O. Risk of Intussusception After Rotavirus Vaccination. Dtsch Arzteblatt Int. 2017 Apr 14;114(15):255–62.

13. Jiang J, Jiang B, Parashar U, Nguyen T, Bines J, Patel MM. Childhood intussusception: a literature review. PloS One. 2013;8(7):e68482.

14. Bahl R, Saxena M, Bhandari N, Taneja S, Mathur M, Parashar UD, et al. Population-Based Incidence of Intussusception and a Case-Control Study to Examine the Association of Intussusception with Natural Rotavirus Infection among Indian Children. J Infect Dis. 2009 Nov;200(s1):S277–81.

15. Jehangir S, John J, Rajkumar S, Mani B, Srinivasan R, Kang G. Intussusception in southern India: Comparison of retrospective analysis and active surveillance. Vaccine. 2014 Aug;32:A99–103.

16. Ministry of Health and Family Welfare, Government of India. Meeting Minutes: Meeting of the National Technical Advisory Group on Immunization (India) [Internet]. Ministry of

For peer review only - http://bmjopen.bmj.com/site/about/guidelines.xhtml

Health & Family Welfare Government of India; 2014 [cited 2020 Nov 3]. Available from: https://www.nitag-resource.org/media-center/document/2220-minutes-of-the-meeting-of-ntagi-held-in-2014

17. Coates EC, Mann-Salinas EA, Caldwell NW, Chung KK. Challenges Associated with Managing a Multicenter Clinical Trial in Severe Burns. J Burn Care Res. 2020 May 2;41(3):681–9.

18. Hagen NA, Stiles CR, Biondo PD, Cummings GG, Fainsinger RL, Moulin DE, et al. Establishing a multicentre clinical research network: lessons learned. Curr Oncol Tor Ont. 2011 Oct;18(5):e243-249.

19. Dewa CS, Durbin J, Wasylenki D, Ochocka J, Eastabrook S, Boydell KM, et al. Considering a multisite study? How to take the leap and have a soft landing. J Community Psychol. 2002 Mar;30(2):173–87.

20. Forjuoh SN. Challenges Associated with Multi-institutional Multi-site Clinical Trial Collaborations: Lessons from a Diabetes Self-Management Interventions Study in Primary Care. J Clin Trials [Internet]. 2015 [cited 2020 Nov 4];05(03). Available from: http://www.omicsgroup.org/journals/challenges-associated-with-multiinstitutional-multisite-clinical-trial-collaborations-lessons-from-a-diabetes-selfmanagement-interventions-study-in-primary-care-2167-0870-1000219.php?aid=55032

21. Das M, Arora N, Bonhoeffer J, Zuber P, Maure C. Intussusception in Young Children: Protocol for Multisite Hospital Sentinel Surveillance in India. Methods Protoc. 2018 Mar 22;1(2):11.

22. Das MK, Arora NK, Gupta B, Sharan A, Kameswari K, Padmalatha P, et al. Intussusception in children aged under two years in India: Retrospective surveillance at nineteen tertiary care hospitals. Vaccine. 2020 Oct;38(43):6849–57.

For peer review only - http://bmjopen.bmj.com/site/about/guidelines.xhtml

23. The INCLEN Intussusception Surveillance Network Study Group. Prospective surveillance for intussusception in Indian children aged under two years at nineteen tertiary care hospitals. BMC Pediatr. 2020 Dec;20(1):413.

24. Fogel DB. Factors associated with clinical trials that fail and opportunities for improving the likelihood of success: A review. Contemp Clin Trials Commun. 2018 Sep;11:156–64.

25. Hurtado-Chong A, Joeris A, Hess D, Blauth M. Improving site selection in clinical studies: a standardised, objective, multistep method and first experience results. BMJ Open. 2017 Jul;7(7):e014796.

26. Gheorghiade M, Vaduganathan M, Greene SJ, Mentz RJ, Adams KF, Anker SD, et al. Site selection in global clinical trials in patients hospitalized for heart failure: perceived problems and potential solutions. Heart Fail Rev. 2014 Mar;19(2):135–52.

27. Potter JS, Donovan DM, Weiss RD, Gardin J, Lindblad R, Wakim P, et al. Site selection in community-based clinical trials for substance use disorders: strategies for effective site selection. Am J Drug Alcohol Abuse. 2011 Sep;37(5):400–7.

28. Potter JS, Donovan DM, Weiss RD, Gardin J, Lindblad R, Wakim P, et al. Site Selection in Community-Based Clinical Trials for Substance Use Disorders: Strategies for Effective Site Selection. Am J Drug Alcohol Abuse. 2011 Sep 1;37(5):400–7.

29. Farquharson K, Murphy KA. Ten Steps to Conducting a Large, Multi-Site, Longitudinal Investigation of Language and Reading in Young Children. Front Psychol [Internet]. 2016 Mar 30 [cited 2020 Nov 4];7. Available from: http://journal.frontiersin.org/Article/10.3389/fpsyg.2016.00419/abstract

30. Greene SM, Geiger AM. A review finds that multicenter studies face substantial challenges but strategies exist to achieve Institutional Review Board approval. J Clin Epidemiol. 2006 Aug;59(8):784–90.

For peer review only

For peer review only - http://bmjopen.bmj.com/site/about/guidelines.xhtml

31. Green LA, Lowery JC, Kowalski CP, Wyszewianski L. Impact of institutional review board practice variation on observational health services research. Health Serv Res. 2006 Feb;41(1):214–30.

32. Maskell NA, Jones EL, Davies RJO, BTS/MRC MIST steering committee. Variations in experience in obtaining local ethical approval for participation in a multi-centre study. QJM Mon J Assoc Physicians. 2003 Apr;96(4):305–7.

For peer review only

For peer review only - http://bmjopen.bmj.com/site/about/guidelines.xhtml

706 **Figures**

707 Figure 1: The steps of the site selection process

708 *Note: TAG: Technical Advisory Group*

709

710 Figure 2: The data collection and management flow for prospective surveillance

711 *Abbreviations: BCDC: Brighton collaboration diagnostic criteria; CCU: Central coordinating*

712 *unit; CRF: case record form; TAG: Technical Advisory Group; Usg: Ultrasound*

713

714 Figure 3: The data collection and management flow for retrospective surveillance

715 *Abbreviations: BCDC: Brighton collaboration diagnostic criteria; CCU: Central coordinating*

716 *unit; CRF: case record form; ICD: International classification of diseases; TAG: Technical*

717 *Advisory Group; Usg: Ultrasound*

718

719 Figure 4: The geographic distribution of the network study sites

720 *Note: The image was created for the research project by the investigators.*

For peer review only

For peer review only - http://bmjopen.bmj.com/site/about/guidelines.xhtml

Supplementary Documents

Supplementary document 1: Potential Study Site Institution Information

Supplementary document 2: Potential Study Site Institution Assessment Report (Site Visit)

Supplementary document 3: The ICD codes for retrieval of the cases from the medical records section

For peer review only

For peer review only - http://bmjopen.bmj.com/site/about/guidelines.xhtml

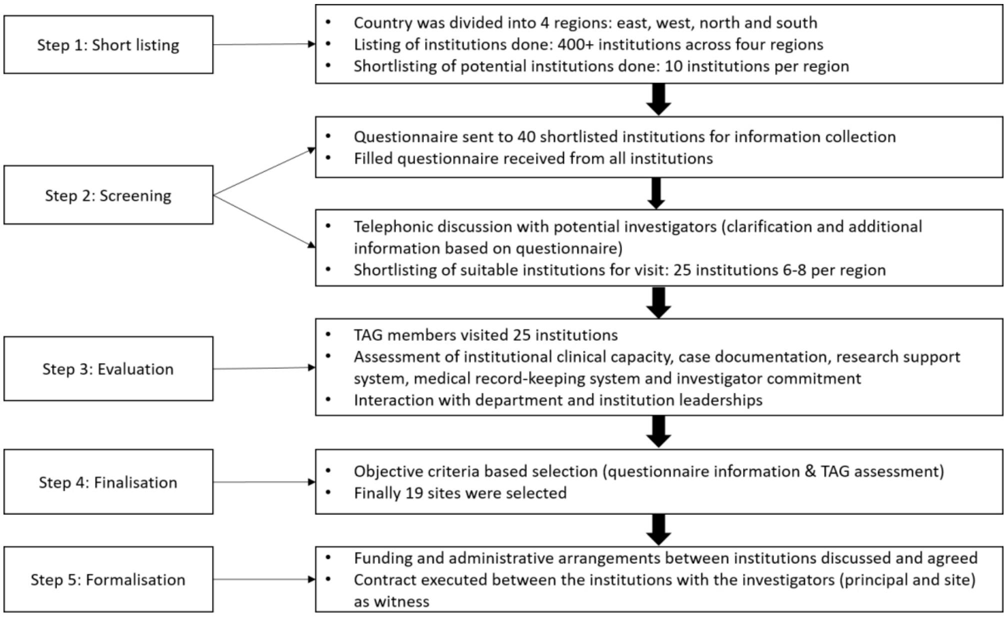

Figure 1: The steps of the site selection process

427x264mm (96 x 96 DPI)

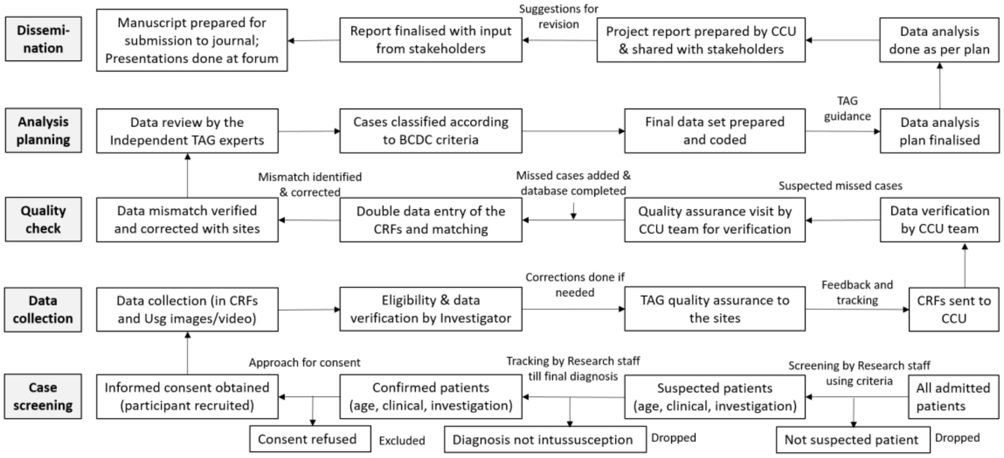

Figure 2: The data collection and management flow for prospective surveillance

516x238mm (96 x 96 DPI)

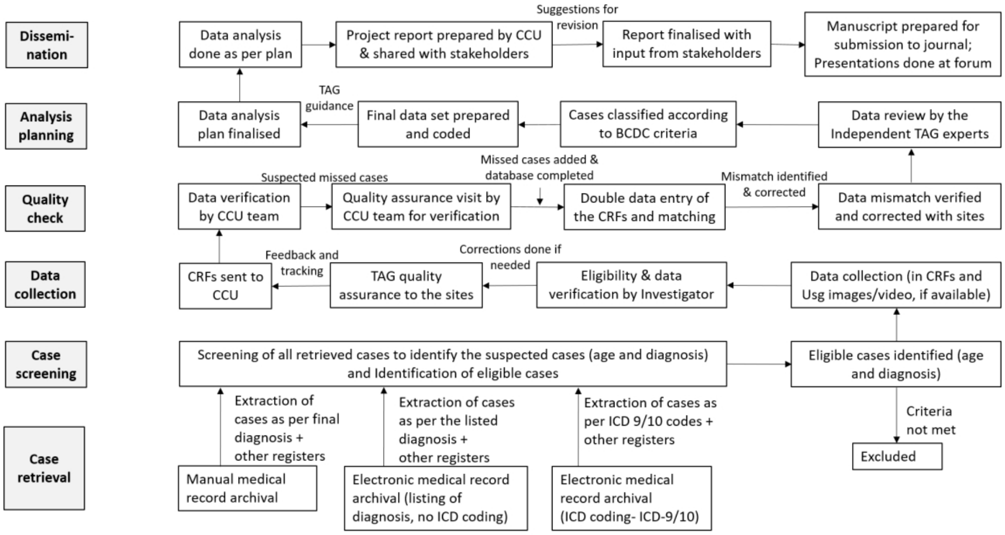

Figure 3: The data collection and management flow for retrospective surveillance

502x270mm (96 x 96 DPI)

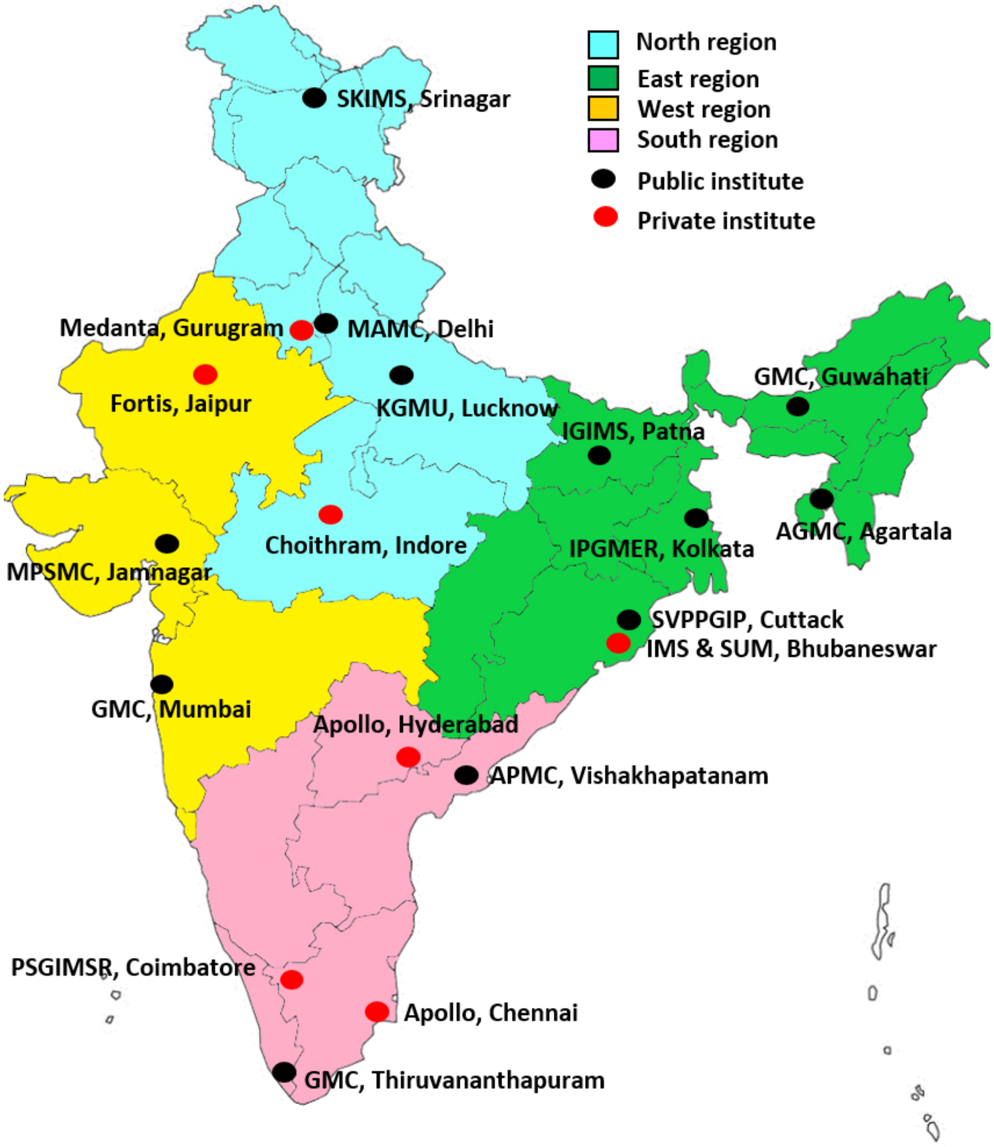

Figure 4: The geographic distribution of the network study sites

228x263mm (96 x 96 DPI)

Intussusception in Infants: Multisite Hospital Based Surveillance in India
Potential Study Site Institution Information

**Institution**	
**Place**	
**State**	

Dear Sir/ Madam,

We are undertaking a study for documentation of intussusception in children under-two years of age at multiple sites in India. This study aims collection of retrospective data over last five-years and prospective data over 18 months on intussusception in children.

As this study involves data retrieval for retrospective data, access to medical records is very critical. Additionally, the access to /availability of clinical case record and radiological images would be very essential for making confirmation of diagnosis.

We request you to provide the following information on facilities and infrastructure at your institute/hospital focusing on diagnosis and management of the intussusception in infants. We highly appreciate your collaboration in answering the following questions and sending them to manoj@inclentrust.org.

A. General information (Please fill the response)

(Please provide information about the concerned departments in the hospital)

Sl no	Item/ Question	Pediatrics	Pediatric surgery	Pediatric gastroenterology	Radiology
1	Number of beds				
2	Number of Specialists (Teaching faculty/ non-teaching specialists)				
3	Academic Residents (MD/DNB/MCH/Fellows)				
4	Non-academic Residents				
5	Number of children (< 5 years) with intussusception admitted in last 1 year				
6	Number of infants (< 1 year) with intussusception admitted in last 1 year				

For peer review only - http://bmjopen.bmj.com/site/about/guidelines.xhtml

B. Clinical services

Sl no	Questions/ Items *(Please mark √in appropriate box; mark as many apply)*	Response Yes	No
**1**	**Availability of round the clock services**		
1.1	Is the pediatric service available round the clock in the hospital?		
1.2	Is the pediatric surgery service available round the clock in the hospital?		
1.3	Is the surgery service available round the clock in the hospital? (If Pediatric Surgery is not available)		
1.4	Is the Emergency Service available round the clock in the hospital?		
1.5	Is the Emergency Registration Service separate from the Central Admission Registration?		
**2**	**What is the system of Central Admission Registration?**		
2.1	Computerized/ electronic admission registration		
2.2	Manual admission registration		
2.3	Any other method (Specify ………………………………………………………)		
**3**	**What is the system of Emergency Registration System?**		
3.1	Computerized/ electronic admission registration		
3.2	Manual admission registration		
3.3	Any other method (Specify ………………………………………………………)		
**4**	**Does the institution have a Centralized Medical Record Index/ Master Patient Index/ Centralized Admission Register?**		
**5**	**If yes, how is it maintained?**		
5.1	Maintained department wise		
5.2	Maintained all departments combined		
5.3	Any other method		
**6**	**Does your hospital give Rotavirus vaccination to the infants?**		
**6.1**	If yes, is the Rotavirus vaccine available in the hospital?		
**6.2**	If yes, is the Rotavirus vaccine purchased by parents from outside?		

For peer review only - http://bmjopen.bmj.com/site/about/guidelines.xhtml

C. Diagnostic services

Sl no	Questions/ Items	Response
**1**	**Availability of the services**	
1.1	How many ultrasound machines are available in the hospital?	
1.2	What is the timing of availability of the ultrasound services?	
2	**Is there a ultrasound facility/ service available in the emergency department/ casualty?**	
**3**	**Who does the ultrasound in emergency hours?** (outside the routine hours)	
3.1	Qualified Radiologist	
3.2	Resident Radiology	
3.3	Any other person (specify ………………………………………..)	
**4**	**How may of the ultrasound machines have facility for digital image storage/ archival?**	
4.1	If yes, for how long the digital images of the ultrasound tests are stored/ archived?	
4.2	Are these digital images retrievable?	
**5**	**What is the reporting system for ultrasound tests?**	
5.1	Only a written report is given	
5.2	Only the film is given	
5.3	Written report and film	
5.4	Images in form of CD/ any storage device	

D. Management of Intussusception cases

Sl no	Questions/ Items<br>*(Please mark √in appropriate box; mark as many apply)*	Response	
		Yes	No
**1**	**Surgery for intussusception**		
1.1	Does the pediatric surgery/ surgery department conduct surgery for intussusception in children?		
1.2	Does the pediatric surgery/ surgery department conduct surgery for intussusception in infants (aged < 1 years)?		
1.3	If No, where are the children/infants are usually referred? (Name of the hospital and place)		
2	**Conservative management of intussusception**		
2.1	Do the children with intussusception are managed by barium enema/ air contrast enema at this hospital?		
2.2	If yes, who does this?		
2.2.1	Radiologist		
2.2.2	Pediatric surgeon		
2.2.3	Pediatrician/ pediatric gastroenterologist		
2.2.4	Any other (specify……………………………………………..)		

For peer review only - http://bmjopen.bmj.com/site/about/guidelines.xhtml

E. Medical Record Archival System

The project requires screening/review of the hospitalized infants in last five years. Thus listing and retrieval of medical records are very essential. The following questions/items focus on the admission, medical record archival and retrieval process. This will allow planning the case record tracking and retrieval at the institution.

Sl no	Questions/ Items<br>*(Please mark √in appropriate box; mark as many apply)*	Response		
		**Yes**	**No**	
**1**	**Is the admission registration for hospital is centralized?**			***If NO, skip to Q 3***
**2**	**If yes, what is the system of admission registration?**			
2.1	Computerized/ electronic admission registration			
2.2	Manual admission registration			
2.3	Any other method (Specify …………………………………...)			
**3**	**Does the institution have a centralized medical record index/ Master Patient Index/ Centralized Admission Register?**			***If NO, skip to Q 5***
**4**	**If yes, what all information can be retrieved from the centralized medical record index/ Master Patient Index/ centralized admission register?**			
4.1	Admission number			
4.2	Date of admission			
4.3	Department of admission			
4.4	Name			
4.5	Age			
4.6	Sex			
4.6	Diagnosis at admission			
4.7	Final diagnosis			
4.8	Outcome (discharge/ death/LAMA)			
4.9	Date of outcome			
**5**	**How the case-sheets (hard copies) are stored/ archived in your hospital/institution for in-patients cases?**			
5.1	Admission number wise			
5.2	Department wise			
5.3	Date of admission wise			
5.4	Date of discharge wise			
5.5	Any other method (specify……………………………………)			
**6**	**How are the case-sheets (hard copies) of in-patient cases indexed/ listed?**			
6.1	Computerized/ electronic listing			
6.2	Manual listing			
6.3	Any other method (specify…………..……………………..)			
6.4	Not listed			

For peer review only - http://bmjopen.bmj.com/site/about/guidelines.xhtml

**7**	**If computerized/ electronic indexing/ listing is done, since when (year) it is being done?**			Year
**8**	**Are the death cases archived separately?**			
**8**	**Does the institute summarize the medical records of hospitalized patients periodically according to any classification system** (ICD 10 or any other)?			
**9**	**Does the institute/hospital have case-records of hospitalized children for last five years readily accessible?**			
**10**	**Please indicate about the listing/recording the information about hospitalized children under Pediatrics Department?**			
10.1	Availability of list/ index of hospitalized children (Like in the list of admitted children maintained by Nurses/ward in-charge)			
11.2	Discharge sheets/ summary of children (copy of discharge sheet maintained by Nurses/ Residents/ ward in-charge)			
**11**	**Please indicate about the listing/recording of the information about children hospitalized under Pediatrics Surgery Department?**			
11.1	Availability of list/index of hospitalized children			
11.2	Operation Theatre Register			
11.3	Discharge sheets/ summary of children			
**12**	**Does the radiology department maintain a register/ listing of patients undergoing investigation?**			
**13**	**What all information can be retrieved from the list/recording?**			
13.1	Admission number			
13.2	Date of investigation			
13.3	Name			
13.4	Age			
13.5	Sex			
13.6	Finding/ Diagnosis			

F. Institute authority approvals

The project requires access to the case-sheets of children with intussusception admitted to your hospital and collection of clinical details about them. Please inform about the approvals from the institute authority(ies) to be obtained for the same.

**Sl no**	**Questions/ Items**	
**1**	**What all permissions from institute/department authorities are needed and the time needed for the same?**	
	Authority	Time needed
1.1		
1.2		
1.3		
1.4		

For peer review only - http://bmjopen.bmj.com/site/about/guidelines.xhtml

**2**	**How frequently (in months) the institute ethics committee meets?**	Monthly
**3**	**What all challenges do you anticipate in this process?**	
**4**	**How all these challenges can be addressed?**	
**5**	**Any other specific comments/ suggestions?**	

G. Investigating Team Members

Please inform about the Investigating team members from the institute along with their affiliations and contact information.

	Name	Department	Designation	Address details (including email and phone)
1				
2				
3				
4				

We thank you for your support and time taken to complete the required information.

Signature Name

Date: ……/……../…………………..

For any further clarification or information, you may contact Dr Manoj Das at manoj@inclentrust.org or (M) 9810203768; (O) 011-47730000

For peer review only

For peer review only - http://bmjopen.bmj.com/site/about/guidelines.xhtml

Intussusception in Infants: Multisite Hospital Based Surveillance in India
Potential Study Site Institution Assessment Report (TAG Site Visit)

**Institution**	
**Place**	
**State**	
**Date of visit**	

Suggestion for the Assessor

Please meet the potential investigators from Pediatrics, Pediatric Surgery, Radiology and In-charge of Medical Record Section to obtain the desired information to be captured in this tool. This is critical to select the suitable study sites to enable quality data on intussusception cases in infants. We highly appreciate your collaboration in providing your responses to the following questions and sending them to manoj@inclentrust.org.

A. General information (Please fill the response)

(Please document the information about the hospital and concerned departments)

Sl no	Item/ Question	Response
1	Total number of beds	
2	Is the Emergency Service available round the clock? (Yes/No)	
3	Is the pediatric service available round the clock? (Yes/No)	
4	Is the pediatric surgery service available round the clock? (Yes/No)	
5	Is the Radiology service available round the clock? (Yes/No)	

B. Pediatrics Department

(Please document the information about the Pediatrics Department)

Sl no	Item/ Question	Response
1	Total number of units in the Department	
2	Total number of in-patient beds	
3	Total number of ICU/PICU beds	
4	Number of faculty/ specialists	
5	Number of senior residents	
6	Number of academic junior residents/ DNB students	
7	Number of non-academic residents	

For peer review only - http://bmjopen.bmj.com/site/about/guidelines.xhtml

8	Is there a pediatric gastroenterology unit/department in the hospital? (Yes/No)	
9	Does any (or >1) pediatrician specializes or focuses on pediatric gastroenterology in the hospital? (Yes/No)	
10	Is there a pediatric gastroenterology OPD/ clinic in the hospital? (Yes/No)	
11	Is there a diarrhea ward/ unit in the hospital for children? (Yes/No)	
12	How are the children with following conditions managed?	Write
12.1	Diarrhoea	
12.2	Blood in stool	
12.3	Infants/young children with pain abdomen or excessive crying	
12.4	Infants/young children with vomiting	
12.5	Infants/young children with surgical abdomen	
12.6	Infants/young children with diagnosis of intussusception	
13	What is usually done, if an infant/ child comes with an ultrasound suggesting intussusception?	Write
13.1	Ultrasound repeated	
13.2	Any additional tests done	
13.3	Admission and evaluation<br>a. Admitted to pediatrics and then transferred to pediatric Surgery/ Surgery department OR<br>b. Directly referred to pediatric Surgery/ Surgery department)	
14	How are the children with intussusception managed? (ask for barium/ air contrast enema)	
15	How are the infants with intussusception managed? (ask for barium/ air contrast enema)	

For peer review only

16	How many patients aged < 5 years with of intussusception were seen in last one year in this department? (approximate number)	
17	How many infants (< 1 years) with of intussusception were seen in last one year in this department? (approximate number)	
18	How many were managed by this department? (approximate number)	
19	Was any of these patients with intussusception were referred to any other hospital? (approximate number)	
20	If yes, what were the most likely reasons?	
21	For the patients getting discharged, is there a copy of the discharge sheet kept in the ward/ in the department? (Yes/No)	
22	If Yes, where is the file/ archival is made?	
23	Please review some case records of admitted infants to document the following.	
23.1	Does the hospital use any structured case record/ follow specific format for clinical history recording? (Yes/No)	
23.2	Is the immunization history recorded in the clinical history? (Yes/No)	
23.3	Is the immunization history recorded with age at vaccination? (Yes/No)	
24	Is the Rotavirus vaccine given at the hospital?	
25	Any other specific observations/ comments	

For peer review only

C. Pediatric Surgery/ Surgery Department

(Please document the information about the Pediatric Surgery Department.)

(Please ask for the Surgery Department, if there is no Pediatric Surgery Department.)

Sl no	Item/ Question	Response
1	Is there a Pediatric Surgery Department in the hospital? (Yes/ No)	
2	Total number of units in the Department	
3	Total number of in-patient beds	
4	Total number of ICU/PS-ICU beds	
5	Number of faculty/ specialists	
6	Number of senior residents	
7	Number of academic junior residents/ DNB students	
8	Number of non-academic residents	
9	If there is no Pediatric Surgeon, how many Surgeons are conducting surgery in children?	
10	If there is no Pediatric Surgeon, how many Surgeons are conducting surgery in infants?	
11	What is usually done, if an infant/ child comes with an ultrasound suggesting intussusception?	Write
11.1	Ultrasound repeated	
11.2	Any additional tests done	
11.3	Admission and evaluation<br>a. Admitted to pediatrics and then transferred to pediatric Surgery/ Surgery department OR<br>b. Directly referred to pediatric Surgery/ Surgery department)	
12	How are the children with intussusception managed? (ask for barium/ air contrast enema)	
13	How are the infants with intussusception managed? (ask for barium/ air contrast enema)	
14	How many patients aged < 5 years with of intussusception were seen in last one year in this department? (approximate number)	
15	How many infants (< 1 years) with of intussusception were seen in last one year in this department? (approximate number)	

For peer review only - http://bmjopen.bmj.com/site/about/guidelines.xhtml

16	How many were managed by this department? (approximate number)	
17	Was any of these patients with intussusception were referred to any other hospital? (approximate number)	
18	If yes, what were the most likely reasons?	
19	Is there a register for listing operated cases? (Yes, No)	
20	If Yes, please check mark the components mentioned in the register.<br>Name: Yes No Sex: Yes No<br>Patient ID: Yes No Age: Yes No<br>Diagnosis: Yes No Date of surgery: Yes No<br>Time taken: Yes No Procedure done: Yes No<br>Outcome: Yes No Surgeon: Yes No<br>Any other:	
21	Is there a register for listing children undergone non-operative intervention (like air-contrast/barium enema) (Yes, No)	
22	If Yes, please check mark the components mentioned in the register.<br>Name: Yes No Sex: Yes No<br>Patient ID: Yes No Age: Yes No<br>Diagnosis: Yes No Date of surgery: Yes No<br>Time taken: Yes No Procedure done: Yes No<br>Outcome: Yes No Surgeon: Yes No<br>Any other:	
23	For the patients getting discharged, is there a copy of the discharge sheet kept in the ward/ in the department? (Yes, No)	
24	If Yes, where is the file/ archival is made?	
25	Please review some case records of admitted infants to document the following.	
25.1	Does the hospital use any structured case record/ follow specific format for clinical history recording? (Yes/No)	
25.2	Is the immunization history recorded in the clinical history? (Yes/No)	
25.3	Is the immunization history recorded with age at vaccination? (Yes/No)	
26	Any other specific observations/ comments	

For peer review only

D. Anesthesia Department and Operation Theatre

(Please document the information about the Anesthesia Department.)

Sl no	Item/ Question	Response
1	Does the anesthetist(s) in the hospital give anesthesia to children and infants? (Yes/ No)	
2	Is the OT well equipped to handle the surgery and anesthesia of infants? (Yes/ No)	
3	Is there a register for listing operated cases? (Yes, No)	
4	If Yes, please check mark the components mentioned in the register.<br>Name: Yes No — Sex: Yes No<br>Patient ID: Yes No — Age: Yes No<br>Diagnosis: Yes No — Date of surgery: Yes No<br>Time taken: Yes No — Procedure done: Yes No<br>Outcome: Yes No — Surgeon: Yes No<br>Any other:	
5	Any other specific observations/ comments	

E. Radiology Department

(Please document the information about the Radiology Department.)

Slno	Item/ Question	Response
1	Total number of Ultrasound machines	
1.1	How may Ultrasound machines have option for digital image saving?	
1.2	Is the digital image saving a routine practice? (Yes/ No)	
1.3	If Yes, for how many days usually the digital images are saved?	
2	Total number of X-ray machines (for barium enema/air contrast enema)	
3	Number of fluoroscopy machine (for barium enema/air contrast enema)	
4	Number of faculty/ specialists	
5	Number of senior residents	
6	Number of academic junior residents/ DNB students	
7	What is the usual timing for ultrasound in the hospital?	From<br>To

For peer review only - http://bmjopen.bmj.com/site/about/guidelines.xhtml

8	What is the arrangement for doing ultrasound beyond routine hours/ nights?	
9	Does the machine(s) used for ultrasound in emergency have facility for digital image saving?	
10	How the reports of ultrasound are given to patients?	
10.1	Only paper report (no film/ image)	
10.2	Paper report with printed paper image (no films)	
10.3	Paper report with films	
10.4	Paper report with CD with digital images	
10.5	Paper report and others available on request/ payment	
10.6	How the reports of barium enema are given to patients?	
10.7	Only paper report (no film/ image)	
10.8	Paper report with films	
10.9	Paper report with CD with digital images	
10.10	Paper report and others (film/CD) on request/ payment	
11	How many patients aged < 5 years with of intussusception were seen in last one year in this department? (approximate number)	
12	How many infants (< 1 years) with of intussusception were seen in last one year in this department? (approximate number)	
13	Do you manage the infants/ children with intussusception by barium/ air contrast enema? (Yes/ No)	
14	If Yes, when was the last such case managed?	
15	Does the department keep a copy of the ultrasound report for record? (Yes, No)	
16	If Yes, where is the file/ archival is made?	
17	Does the radiology department maintain a register/ listing of patients undergoing investigation (ultrasound/ barium)?	
18	What all information can be retrieved from the list/recording?	
18.1	Admission number	
18.2	Date of investigation	
18.3	Name	
18.4	Age	
18.5	Sex	
18.6	Findings/ Diagnosis	
19	Any other specific observation/ comment	

For peer review only - http://bmjopen.bmj.com/site/about/guidelines.xhtml

F. Case Registration System

(Please document about the case registration system of the hospital for in-patient cases)

Sl no	Questions/ Items	Response (Yes/No)
1	What is the system of Central In-patient Admission Registration?	
1.1	Computerized/ electronic admission registration	
1.2	Manual admission registration	
1.3	Any other method (Specify ………………………………………………………….)	
2	If the Registration system is manual, how is the Registration Index maintained?	
2.1	Maintained department wise	
2.2	Maintained all departments combined	
2.3	Any other method	
3	Is the Central In-patient Admission Registration system available round the clock?	
4	Is the Emergency Case Registration System separate from the Central in-patient registration?	
5	If Yes, What is the system of Emergency Registration System?	
5.1	Computerized/ electronic admission registration	
5.2	Manual admission registration	
5.3	Any other method (Specify ………………………………………………………….)	
6	What all information can be retrieved from the Central In-patient Admission Registration Information? (Yes/No)	
6.1	Admission number	
6.2	Date of admission	
6.3	Department of admission	
6.4	Name	
6.5	Age	
6.6	Sex	
6.7	Diagnosis at admission	
6.8	Final diagnosis	
6.9	Outcome (discharge/ death/LAMA)	
6.10	Date of outcome	
7	Any specific observation/ comments	

For peer review only - http://bmjopen.bmj.com/site/about/guidelines.xhtml

G. Medical Record Archival System

The project requires screening/review of the hospitalized infants in last five years. Thus listing and retrieval of medical records are very essential. The following questions/items focus on the admission, medical record archival and retrieval process. This will allow planning the case record tracking and retrieval at the institution.

Sl no	Questions/ Items	Response (Yes/No)
1	Does your hospital have a Medical records Department/ Division?	
2	How frequently the in-patient case-sheets (hard copies) are sent to Medical Records Department/ Division from the departments?	
3	How the in-patient case-sheets (hard copies) are stored/ archived in your hospital/institution?	
3.1	Admission number wise	
3.2	Department wise	
3.3	Date of admission wise	
3.4	Date of discharge wise	
3.5	Any other method of organization (specify…………………………………………………)	
4	How are the in-patient case-sheets (hard copies) indexed/ listed?	
4.1	Computerized/ electronic listing	
4.2	Manual listing	
4.3	Any other method (specify……………………………………………………..)	
4.4	Not listed	
5	If computerized/ electronic indexing/ listing is done, since when (year) it is being done?	Year
6	Does the institute summarize the medical records of hospitalized patients periodically according to any classification system (ICD 10 or any other)?	
7	If yes, who does the classification labeling of the cases?	
8	How frequently the classification labeling of the cases is done?	
9	Is there any policy by the hospital regarding duration of archival/ storage of the in-patient case-sheets (hard copies)?	
10	If Yes, for how many years the hard copies are stored?	

For peer review only

11	Is there any effort for digital storage of the in-patient case-sheets?	
12	If yes, since when? (year)	
13	What is the process of retrieval of the case records from the Medical Records Department? (please describe focusing on the following)<br>a. The process of application:<br>b. Time needed for approval<br>c. Time needed for retrieval and receipt of the case records<br>d. Any limitation on the number of case records issued in one go	
14	How can we retrieve the list of children admitted with diarrhea to the hospital in month of June 2010? (please ask the concerned official for demonstrating the same)	
15	If retrieved, please document the number of children admitted with diarrhea in the month of June 2010.	
16	Any other challenge in retrieval of the in-patient case-sheets (hard copies) for last five years for the pediatrics and pediatric surgery departments?	
17	Any specific observation/ comments	

For peer review only - http://bmjopen.bmj.com/site/about/guidelines.xhtml

H. Institute authority and regulatory approvals

The project requires retrieval of the case-sheets of children with intussusception admitted to your hospital and collection of clinical details about them. Please inform about the approvals from the institute authority(ies) to be obtained for the same.

**Sl no**	**Questions/ Items**	
**1**	**What all permissions from institute/department authorities are needed and the time needed for the same?**	
	Authority	Time needed
1.1		
1.2		
1.3		
1.4		
1.5		
**2**	**How frequently (in months) the institute ethics committee meets?**	
**3**	**Meeting with Department Head and Faculty Colleagues**<br>**What is your assessment about the reputation of the potential investigator and support anticipated from the department?**	
**4**	**Meeting with Institute Head or Research Head**<br>**What is your assessment about the research environment in the institute and facilitations anticipated from the department?**	
**5**	**What all challenges do you anticipate in this process?**	

For peer review only - http://bmjopen.bmj.com/site/about/guidelines.xhtml

6	**How all these challenges can be addressed/mitigated?**
**7**	**Any other specific comments/ suggestions?**

I. Investigating Team Members

Please inform about the Investigating team members from the institute along with their affiliations and contact information.

	Department	Name	Designation	Address details (including email and phone)
1	Pediatrics			
2	Pediatric Surgery			
3	Radiology			
4	Medical Records			
5	Any other			

We thank you for your support and time taken to complete the required information.

Any other observations/ comments

Signature   Name

Date: ……/……../…………………..

For peer review only - http://bmjopen.bmj.com/site/about/guidelines.xhtml

Supplementary document 3:

The International Classification of Diseases (ICD) codes for retrieval of the cases from the medical records section

Clinical conditions considered as suspected cases	Codes	
	ICD 10	ICD 9
Intussusception	K56.1	560.0
Volvulus	K56.2	560.2
Gallstone ileus	K56.3	560.31
Other impaction of intestine	K56.4	560.30
Intestinal adhesions with obstruction	K56.5	560.81
Other and unspecified intestinal obstruction	K56.6	560.9
Ileus, unspecified	K56.7	560.1
Paralytic ileus	K56.0	

ICD: International Classification of Diseases

For peer review only

BMJ Open

Experience of establishing and coordinating a nationwide network for bi-directional intussusception surveillance in India: Lessons for multisite research studies

Journal:	*BMJ Open*
Manuscript ID	bmjopen-2020-046827.R2
Article Type:	Original research
Date Submitted by the Author:	22-Apr-2021
Complete List of Authors:	Study Group, The INCLEN Intussusception Surveillance; The INCLEN Trust International<br>Das, Manoja; The INCLEN Trust International
**Primary Subject Heading**:	Public health
Secondary Subject Heading:	Global health, Paediatrics
Keywords:	PUBLIC HEALTH, Community child health < PAEDIATRICS, EPIDEMIOLOGY, Paediatric gastroenterology < PAEDIATRICS

SCHOLARONE™
Manuscripts

I, the Submitting Author has the right to grant and does grant on behalf of all authors of the Work (as defined in the below author licence), an exclusive licence and/or a non-exclusive licence for contributions from authors who are: i) UK Crown employees; ii) where BMJ has agreed a CC-BY licence shall apply, and/or iii) in accordance with the terms applicable for US Federal Government officers or employees acting as part of their official duties; on a worldwide, perpetual, irrevocable, royalty-free basis to BMJ Publishing Group Ltd ("BMJ") its licensees and where the relevant Journal is co-owned by BMJ to the co-owners of the Journal, to publish the Work in this journal and any other BMJ products and to exploit all rights, as set out in our licence.

The Submitting Author accepts and understands that any supply made under these terms is made by BMJ to the Submitting Author unless you are acting as an employee on behalf of your employer or a postgraduate student of an affiliated institution which is paying any applicable article publishing charge ("APC") for Open Access articles. Where the Submitting Author wishes to make the Work available on an Open Access basis (and intends to pay the relevant APC), the terms of reuse of such Open Access shall be governed by a Creative Commons licence – details of these licences and which Creative Commons licence will apply to this Work are set out in our licence referred to above.

Other than as permitted in any relevant BMJ Author's Self Archiving Policies, I confirm this Work has not been accepted for publication elsewhere, is not being considered for publication elsewhere and does not duplicate material already published. I confirm all authors consent to publication of this Work and authorise the granting of this licence.

For peer review only

Original article

Title: Experience of establishing and coordinating a nationwide network for bi-directional intussusception surveillance in India: Lessons for multisite research studies

Short title: Experience from multisite intussusception surveillance network in India

Author Information

The INCLEN Intussusception Surveillance Network Study Group

The INCLEN Trust International, Okhla Industrial Area, Phase 1, New Delhi -110020, India

E-mail: drmanojkdas@gmail.com

Corresponding Author

Manoja Kumar Das

Director Projects, The INCLEN Trust International, F1/5, Okhla Industrial Area, Phase 1, New Delhi -110020, INDIA

Phone: Off: +91-11-47730000; Mob: +91-9810203768; Fax: +91-11-47730001

E-mail: manoj@inclentrust.org

Word count: Abstract- 292 words; Main document- 3143 words

For peer review only

For peer review only - http://bmjopen.bmj.com/site/about/guidelines.xhtml

Author information

'The INCLEN Intussusception Surveillance Network Study Group' list of authors

1. Manoja Kumar Das

Director Projects, The INCLEN Trust International, New Delhi, India

Email: manoj@inclentrust.org

2. Narendra Kumar Arora

Executive Director, The INCLEN Trust International, New Delhi, India

Email: nkarora@inclentrust.org

3. Arindam Ray

Senior Program Officer, Bill and Melinda Gates Foundation, India Country Office, New Delhi, India

Email: arindam.ray@gatesfoundation.org

4. Ashish Wakhlu

Professor, Department of Paediatric Surgery, King George's Medical University, Lucknow, Uttar Pradesh, India

Email: ashish_wakhlu@hotmail.com

5. Bhadresh R Vyas

Professor, Department of Paediatrics, MP Shah Government Medical College, Jamnagar, Gujarat, India

Email: bhadreshrvyas@yahoo.co.uk

6. Javeed Iqbal Bhat

Assistant Professor, Department of Paediatrics, Sher-I-Kashmir Institute of Medical Sciences, Srinagar, Jammu & Kashmir, India

Email: drjaveediqbal@gmail.com

7. Jayanta K. Goswami

For peer review only

For peer review only - http://bmjopen.bmj.com/site/about/guidelines.xhtml

Professor, Department of Paediatric Surgery, Gauhati Medical College, Guwahati, Assam, India

Email: jayantagoswami@hotmail.com

8. John Mathai

Professor, Department of Paediatrics, PSG Institute of Medical Sciences, Coimbatore, Tamil Nadu, India

Email: psg_peds@yahoo.com

9. Kameswari K.

Professor, Department of Paediatric Surgery, Andhra Medical College, Vishakhapatnam, Andhra Pradesh, India

Email: kameswari1956@gmail.com

10. Lalit Bharadia

Consultant Paediatric Gastroenterologist, Fortis Escorts Hospital, Jaipur, Rajasthan, India

Email: lalitbharadia@gmail.com

11. Lalit Sankhe

Assistant Professor, Department of Community Medicine, Grant Medical College & JJ Hospital, Mumbai, Maharashtra, India

Email: sankhelalit@yahoo.com

12. Ajaya Kumar M.K.

Professor, Department of Paediatric Surgery, Government Medical College & SAT Hospital, Thiruvananthapuram, Kerala, India

Email: drajaykumarmk@gmail.com

13. Neelam Mohan

Consultant Paediatrics Gastroenterology, Medanta—The Medicity, Gurgaon, Haryana, India

For peer review only

Email: drneelam@yahoo.com

14. Pradeep K. Jena

Professor, Department of Paediatric Surgery, SCB Medical College, Cuttack, Odisha, India

Email: drpkjena@gmail.com

15. Rachita Sarangi

Professor, Department of Paediatrics, IMS & SUM Medical College & Hospital, Bhubaneswar, Odisha, India

Email: rachitapaedia@gmail.com

16. Rashmi Shad

Consultant Paediatrics, Choithram Hospital and Research Centre, Indore, Madhya Pradesh, India

Email: drrashmishad@yahoo.com

17. Sanjib K. Debbarma

Associate Professor, Department of Paediatrics, Agartala Government Medical College, Agartala, Tripura, India

Email: dr_sanjibdb@rediffmail.com

18. Shyamala J.

Consultant Paediatrics, Apollo Hospitals, Chennai, Tamil Nadu

Email: jaymoorthy@hotmail.com

19. Simmi K. Ratan

Professor, Department of Paediatric Surgery, Maulana Azad Medical College, Delhi, India

Email: drjohnsimmi@yahoo.com

20. Suman Sarkar

Assistant Professor, Department of Paediatrics, Institute of Post Graduate Medical Education and Research, Kolkata, West Bengal, India

For peer review only

Email: dr.sumansarkar@gmail.com

21. Vijayendra Kumar

Professor, Department of Paediatric Surgery, Indira Gandhi Institute of Medical Sciences, Patna, Bihar, India

Email: drvijayendrakr@rediffmail.com

22. Anand P. Dubey

Professor, Department of Paediatrics, Maulana Azad Medical College, Delhi, India

Email: Anand_dubey52@hotmail.com

23. Atul Gupta

Consultant Paediatric Surgery, Fortis Escorts Hospital, Jaipur, Rajasthan, India

Email: atulgupta26878@gmail.com

24. Bikasha Bihary Tripathy

Associate Professor, Department of Paediatric Surgery, IMS & SUM Medical College & Hospital, Bhubaneswar, Odisha, India

Email: bbtripathy.dr@gmail.com

25. Cenita J. Sam

Professor, Department of Paediatric Surgery, PSG Institute of Medical Sciences, Coimbatore, Tamil Nadu, India

Email: cenitasivamani@yahoo.com

26. Gowhar Nazir Mufti

Assistant Professor, Department of Paediatric Surgery, Sher-I-Kashmir Institute of Medical Sciences, Srinagar, Jammu & Kashmir, India

Email: gowharmufti@yahoo.co.in

27. Harsh Trivedi

For peer review only

Professor, Department of Paediatric Surgery, MP Shah Government Medical College, Jamnagar, Gujarat, India

Email: trivediharsh@indiatimes.com

28. Jimmy Shad

Consultant Paediatric Surgery, Apollo Hospitals, Chennai, Tamil Nadu, India

Email: shadjimmy@yahoo.co.in

29. Kaushik Lahiri

Consultant, Department of Paediatric Surgery, Gauhati Medical College, Guwahati, Assam, India

Email: Kaushik_1671979@rediffmail.com

30. Meera Luthra

Consultant Paediatric Surgery, Medanta- The Medicity, Gurgaon, Haryana, India

Email: meera.luthra@medanta.org

31. Padmalatha P

Professor, Department of Paediatrics, Andhra Medical College, Vishakhapatnam, Andhra Pradesh, India

Email: padmap_2000@yahoo.com

32. Rakesh Kumar

Associate Professor, Department of Paediatrics, Indira Gandhi Institute of Medical Sciences, Patna, Bihar, India

Email: drjaiswalrakesh@gmail.com

33. Ruchirendu Sarkar

Professor, Department of Paediatric Surgery, Institute of Post Graduate Medical Education and Research, Kolkata, West Bengal, India

Email: ruchirendu@gmail.com

For peer review only

For peer review only - http://bmjopen.bmj.com/site/about/guidelines.xhtml

34. Santosh Kumar A.

Professor, Department of Paediatrics, Government Medical College & SAT Hospital, Thiruvananthapuram, Kerala, India

Email: hod.pediatrics.sath@gmail.com

35. Subrat Kumar Sahoo

Associate Professor, Department of Paediatric Surgery, IMS & SUM Medical College & Hospital, Bhubaneswar, Odisha, India

Email: dr_subratsahoo@yahoo.com

36. Sunil K. Ghosh

Associate Professor, Department of Pediatric Surgery, Agartala Government Medical College, Agartala, Tripura, India

Email: sunilkrghosh752@gmail.com

37. Sushant Mane

Assistant Professor, Department of Paediatrics, Grant Medical College & JJ Hospital, Mumbai, Maharashtra, India

Email: drsush2006@gmail.com

38. Bashir Ahmad Charoo

Professor, Department of Paediatrics, Sher-I-Kashmir Institute of Medical Sciences, Srinagar, Jammu & Kashmir, India

Email: charoobash@gmail.com

39. Rajendra Prasad G.

Professor, Department of Paediatric Surgery, Andhra Medical College, Vishakhapatnam, Andhra Pradesh, India

Email: dr.grajendraprasad@gmail.com

40. Harish Kumar S

For peer review only

Paediatrics Radiologist, Apollo Hospitals, Chennai, Tamil Nadu, India

Email: drharishkumar.s@gmail.com

41. Jothilakshmi K.

Professor, Department of Paediatrics, PSG Institute of Medical Sciences, Coimbatore, Tamil Nadu, India

Email: drjothi.cbe@gmail.com

42. Nihar Ranjan Sarkar

Associate Professor, Department of Radiology, Institute of Post Graduate Medical Education and Research, Kolkata, West Bengal, India

Email: dr.niharsarkar@gmail.com

43. Pavai Arunachalam

Professor, Department of Paediatric Surgery, PSG Institute of Medical Sciences, Coimbatore, Tamil Nadu, India

Email: pavai4321@yahoo.com

44. Satya S.G. Mohapatra

Professor, Department of Radiology, IMS & SUM Medical College & Hospital, Bhubaneswar, Odisha, India

Email: drartifact@gmail.com

45. Saurabh Garge

Consultant Paediatric Surgery, Choithram Hospital and Research Centre, Indore, Madhya Pradesh, India

Email: saurabhgarge8@gmail.com

For peer review only

Abstract

Objectives: To document and share the process of establishing the nationally representative multisite surveillance network for intussusception in India, coordination, data management and lessons learned from the implementation.

Design: This study combined both retrospective and prospective surveillance approaches.

Setting: 19 tertiary care institutions were selected in India considering the geographic representation and public and private mix

Participants: All children under-two years of age with intussusception

Primary and secondary outcome measures: The experience of site selection, regulatory approvals, data collection, quality assurance and network coordination were documented.

Results: The site selection process involved systematic and objective four steps including shortlisting of potential institutions, information seeking and telephonic interaction, site visits and site selection using objective criteria. Out of over 400 hospitals screened across India, 40 potential institutions were shortlisted and information was sought by questionnaire and interaction with investigators. Out of these, 25 institutes were visited and 19 sites were finally selected to participate in the study. The multi-step selection process allowed filtering and identification of sites with adequate capacity and motivated investigators. The retrospective surveillance documented 1588 cases (range: 14-652 cases/site) and prospective surveillance recruited 621 cases (range: 5-191 cases/site). The multilayer quality assurance measures monitored and ensured protocol adherence, complete record retrieval and data completeness. The key challenges experienced included time taken for obtaining regulatory and ethical

approvals, which delayed completion of the study. Ten sites continued with another multisite vaccine safety surveillance study.

Conclusion: The experience and results of this systematic and objective site selection method in India are promising. The systematic multi-step site selection and data quality assurance methods presented here are feasible and practical. The lessons from the establishment and coordination of this surveillance network can be useful in planning, selecting the sites and conducting multisite and surveillance studies in India and developing countries.

Keywords: Surveillance, Multisite study, Site selection, Quality assurance, Coordination, Challenges, Intussusception, India

peer review only

For peer review only - http://bmjopen.bmj.com/site/about/guidelines.xhtml

Article summary

Strengths and limitations of this study

- Provides a systematic study site selection process using objective methodology
- Documents the experience with prospective and retrospective surveillance data collection with multi-layered data quality assurance process
- The study site selection and quality assurance measures may serve as reference for suitable adaptation in different research topics and contexts
- The lessons are applicable only to the multisite studies.
- The documentation of contractual and financial management challenges may be limited.

For peer review only

For peer review only - http://bmjopen.bmj.com/site/about/guidelines.xhtml

Introduction

Intussusception is an acute emergency in children and most commonly occurs in infants aged 4-10 months (1)(2). Although some are transient and resolve spontaneously, if not intervened timely, it may lead to bowel ischemia and perforation and may even be fatal (1). Intussusception has been reported as an adverse reaction with rotavirus vaccines (RVV) with variable risks ranging from no increased risk to low risk (1-2 additional cases per 100,000 vaccinated infants) across different countries with different RVVs (3)(4)(5)(6)(7)(8)(9)(10)(11)(12). The intussusception background rate varies widely across different countries, 9 (Bangladesh) to 328 (South Korea) per 100,000 infants (13). Limited information from India reported its incidence between 17.7 to 254 cases per 100,000 child-years (14)(15). Several other studies were single or few centre studies and varied in case definitions, age groups, reference periods, and methodology (12)(13)(14).

In view of the vaccine safety concern and limited information on intussusception in India, the National Technical Advisory Group on Immunization recommended vaccine safety surveillance along with RVV introduction (16). This surveillance network aimed to generate background information on intussusception epidemiology Indian children to inform the policy and program related to RVV introduction and serve as baseline for future surveillance to identify any change after vaccine introduction and address the vaccine safety concerns. The objectives were to: (1) establish a surveillance network of public and private hospitals ensuring regional representation and data capturing system; (2) undertake retrospective surveillance to document the intussusception epidemiology over past five years; (3) undertake prospective surveillance to document the intussusception epidemiology over 18 months and potential linkage with RVVs; and (4) build capacity of the investigators and institutions.

Multicentre studies face several challenges related to design, site selection, regulatory approvals, study conduct, coordination, data management and dissemination (17)(18)(19)(20).

For peer review only - http://bmjopen.bmj.com/site/about/guidelines.xhtml

The lessons from multisite studies in India are limited. The intussusception surveillance study has been successfully completed and the network has evolved over time to undertake more vaccine safety studies. The purpose of this paper is to share the process of network establishment, coordination, data management and lessons learned from such nationwide network research.

Methods

Study design: This study combined both retrospective and prospective surveillance. The experiences and lessons presented here are based on the concurrent documentation of processes and retrospective review of the study documents.

Study governance: The Central Coordinating Unit (CCU) constituted investigators, research staffs and administrative and finance team. For technical integrity and implementation monitoring, a Technical Advisory Group (TAG) was constituted with 17 Indian and international experts in vaccine safety, surveillance, immunization program, public health, child health, paediatric surgery, radiology and medical record system, representation from Ministry of Health and Indian Council of Medical Research.

Site selection: We followed a four-step systematic study site selection process (Figure 1). *Step 1 (Shortlisting):* We categorised the states into four regions (north, south, east and west) and screened the tertiary care hospitals (medical colleges and private hospitals) in these regions from the websites. *Step 2 (Screening):* We solicited information about the case load, clinical and diagnostic capacities (paediatrics, paediatric surgery, and radiology), medical record system, ethical and administrative approvals from the shortlisted institutions using questionnaire and telephonic interactions (Supplementary document 1). *Step 3 (Evaluation):* A TAG member visited the institutions to assess the institution capacity (clinical facility, case documentation, research support system, medical record-keeping system) and interacted with

For peer review only - http://bmjopen.bmj.com/site/about/guidelines.xhtml

the potential investigator(s), department and institution leaderships to assess the commitment, institution support and research environment (Supplementary document 2). *Step 4 (Site finalisation):* Based on the questionnaire, interaction with investigators and TAG member feedback, the study sites were finalised.

Protocol finalisation and study tool development: In multisite research projects, consensus building among the investigators on the study protocol, case record forms (CRFs), data management and monitoring are critical. The study protocol and CRFs were shared and piloted at the study sites. The protocol, study tools, implementation, data management and monitoring were discussed in detail during protocol finalisation workshop involving lead investigators from all sites and TAG members.

Regulatory procedures: As this study involved international funding, the approval from Health Ministry Screening Committee (HMSC) was obtained. The study protocol documents were submitted to the participating institutes for ethical approvals. Following the ethical approval, the study was implemented at the sites.

Study implementation process: Before implementation of the study at the sites, the following four items had to be completed: (1) institute administrative approval; (2) ethical approval; (3) agreement executed with the institution; and (4) research staff selected and trained.

Data collection, management and monitoring: Separate CRFs and log sheets were prepared for the retrospective and prospective data collection. The retrospective data collection involved multiple sources; case records, registers from different departments and operation theatres and medical records section. For case record retrieval, the sites following International Classification of Diseases (ICD, ICD-9 or ICD-10) system, the ICD codes for intussusception or acute abdomen conditions were used (Supplementary document 3) (published in methodology paper) (21). At the other sites, cases were identified as per the diagnoses. For the prospective data collection, all admitted children were screened to identify the suspected cases,

For peer review only - http://bmjopen.bmj.com/site/about/guidelines.xhtml

who were followed till final diagnosis. The children with intussusception were recruited and data collected. A weekly reporting from the sites was solicited for tracking progress. Initially a weekly call with the study teams and later fortnightly to monthly calls were done to monitor the progress and address challenges. Based on the data collection progress reports, a monthly bulletin was prepared indicating the progresses and data quality. The completed study tools were sent periodically to CCU. The data received at CCU was reviewed for completeness, correctness and queries were resolved with reference to the source documents. Double data entry was done using customised data entry platform with inbuilt data matching program. The data was archived in the server with authorised access and regular backup. Data analysis was done by under guidance of TAG. The data collection, flow and management followed for prospective and retrospective surveillance are shown in figure 2 and figure 3.

Quality assurance measures: Multiple quality assurance measures focused on protocol adherence and data quality. After initiation of data collection, TAG members visited each study site during the retrospective surveillance to verify: (a) protocol adherence; (b) identification of suitable cases; and (c) data abstraction. The corrections/clarifications and repeat training was imparted as per TAG observations. After six months, during the prospective surveillance, TAG member again visited to review the: (a) patient screening; (b) tracking and eligible patients identification; (c) obtaining consent; and (d) data abstraction.

Patient and Public Involvement: No patients or the public were involved in the design, conduct, reporting, and dissemination of research.

For peer review only - http://bmjopen.bmj.com/site/about/guidelines.xhtml

Results

Study timelines: The study was sanctioned in November 2014. The HMSC application was submitted in December 2014 and approved in May 2015. The formal study site selection initiated after the HMSC approval and was completed by August 2015. The protocol finalisation workshop was held in October 2015. Following the ethical approvals, the retrospective surveillance was initiated at majority of the sites in February 2016. The prospective surveillance was initiated in April 2016 and continued through September 2017. The data entry, cleaning and analysis and report drafting were completed by June 2018.

Study site selection: Out of over 400 hospitals screened across regions, 40 potential hospitals/institutions (10 per region) were shortlisted. While preparing the database, many institutions didn't have adequate information on their websites about the facilities and faculty members or specialists. The potential investigators from these 40 shortlisted institutions were invited to submit desired information using a questionnaire via email and all responded. Telephonic discussions was held with the potential investigator(s) from these institutions (45-60 minutes) for additional information or clarifications. Based on the criteria and consultation with TAG, 25 institutes were visited. Based on the questionnaire, interactions and TAG member assessment, 19 institutions (north region- 5 sites, 3 public and 2 private; south region- 5 sites, 2 public and 3 private; east region- 6 sites, 5 public and 1 private; west region- 3 sites, 2 public and 1 private) were selected (figure 4). The study originally planned for 17 sites. Two more sites from the states where RVV was scheduled for introduction were added later. Several potential investigators requested the CCU team to discuss with their institute leadership for permission and allow him/her to lead the project. The CCU team succeeded in all expect two institutions, which couldn't be included. The site selection process was delayed by seven months due to the delay in HMSC approval.

For peer review only - http://bmjopen.bmj.com/site/about/guidelines.xhtml

Regulatory approvals: The ethics approvals from the sites needed average 4 months (range: 1 to 8 months). No protocol amendment was needed.

Data collection: During July 2010 and March 2016 (retrospective surveillance period), out of 42,866 admitted under-two years children, 2092 suspected cases were identified and 1588 confirmed intussusception cases were recruited (22). The cases recruited at study sites ranged from 14 to 652. While five sites documented <20 cases each, two sites contributed >100 cases each. During April 2016 and September 2017 (prospective surveillance period), out of 6300 hospitalised under-two years children, 1203 suspected cases were identified and 621 confirmed intussusception cases were recruited (23). The cases recruited at the study sites ranged from 5 to 191 cases. While seven sites recruited <10 cases each, three sites contributed >50 cases each. At one study site, very few cases were retrieved from the retrospective and prospective surveillance, contrary to the anticipation. The descriptive data analysis was done at pooled and regional levels to document the epidemiology of intussusception. The variables and data parameters between the regions were compared to identify the similarities and differences.

Medical record retrieval: Twelve (seven private and five public) institutions had electronic medical record (EMR) system. The ICD system was used at all sites except the two private hospitals, where the diagnosis was used for listing and archival. The medical records were accessible at all study sites except one site, where the research staff faced challenge in timely and adequate access to the medical records. The medical records required organisation at this site and thus the retrospective data collection took longer. The record retrieval was quicker at institutions with EMR system and retrospective data collection was completed in 2-4 months. At two sites the retrospective data collection took longer 6 and 10 months, due to higher case load and challenge in record retrieval, respectively.

Radiology documentation: The ultrasound digital images and report were available for all prospectively recruited cases. There were variations in the ultrasound finding documentations

For peer review only - http://bmjopen.bmj.com/site/about/guidelines.xhtml

and details in the reports, which required clarification from the radiologist for data capturing. Ultrasound reports were available at all but one hospital, where the findings were recorded in the case sheet. Digital records of ultrasounds were stored for minimum three months to five years. These ultrasound reports, digital films and clinical records were reviewed by independent TAG members for confirmation and classification according to Brighton Collaboration Criteria (1).

Vaccination information: Majority of the parents were not carrying the vaccination card at the time of hospitalisation and about half of them came from outside the city. The research staffs pursued to obtain the vaccination card during hospitalisation and after discharge. The vaccination card after discharge was obtained through email, mobile message and also self-addressed and stamped envelopes handed over to the parents. The vaccination information was available for 78.4% of the prospective cases.

Clinical case management: At five sites only surgery was done. At two sites reduction was available during daytime only and surgery during other period and at twelve sites both methods were available always.

Data quality assurance: TAG members made two quality assurance visits, first during the retrospective surveillance period, second during the prospective surveillance period and provided written feedback. The retrospective surveillance was at risk of missing cases. As it was difficult to compare the absolute case numbers across the centres and periods, we derived intussusception case rate per 1000 paediatric surgery admissions. After completion of one year of prospective surveillance, third quality assurance visit was made by the data management team to 11 sites with >30% differences in the case rates between retrospective and prospective periods. The team targeted verifying the confirmed cases, suspected cases and total admissions during 2015, 2016, and 2017 (until visit). The team identified three missed cases (one each at three sites).

For peer review only - http://bmjopen.bmj.com/site/about/guidelines.xhtml

Investigators commitment and transition: Despite the systematic selection process, the involvement of the investigators in the day-to-day operations and data verification was lesser than expected at three sites. At three sites the lead investigators transitioned without any impact on the study activities.

Research staff issues: Although one research staff per site was planned, at seven sites two research staffs were engaged considering the efforts needed for data retrieval and case load. These research staffs were trained through regional training workshops followed by hands-on training. At eight sites the research staffs changed during the study period, once at six sites and twice at two sites. All the new staffs were trained by the CCU team member through site visit followed by virtual support. The investigators swiftly ensured replacements and no loss of cases. At CCU, there were three rounds of research staff transition.

Intra-and inter-department coordination: At all institutions the nurses, residents and faculty members supported the surveillance. The nurse and residents at most places informed the research staff about any confirmed case, even on holidays. If the patient was discharged on any holiday, the research staffs attended and collected the data. Intra- and inter-departmental coordination for data collection were smooth at all sites except two sites, where the participation from paediatric department was limited. As most of the intussusception cases were directly admitted to or transferred to paediatric surgery department on diagnosis, it didn't affect the case screening and recruitment.

Financial management: In view of the foreign funding source and Foreign Contribution Regulation Act (FCRA) regulation, the funds couldn't be transferred to most institutes without FCRA approval. Thus, the fund was managed by the CCU for most of the institutes. Despite no fund transferred to the institutes, agreements were executed between the institutes with the investigators as the witnesses. We experienced challenges with some institutes in explaining the FCRA obligations prior to agreement execution.

For peer review only - http://bmjopen.bmj.com/site/about/guidelines.xhtml

Study tenure: Although planned for 30 months, the study was completed in 43 months. The delays in HMSC and ethics approval at sites were the key reasons for the delay.

Dissemination: Four peer-reviewed manuscript have been published and eight manuscripts are under review or in preparation. The manuscripts have all the site investigators as authors, either individually or as group. The findings have been shared with the Ministry of Health and Family Welfare, World Health Organisation and other key partners. Over 22 oral presentations had been made at national, regional and international meetings.

Capacity building and transition: After this study, several sites were included in other studies coordinated by the CCU, ten sites in one study and four sites in another study. Four site investigators collaborated in other research projects. Several of the research staffs at these sites continued with the new studies.

Discussion

This multicentre sentinel surveillance successfully generated clinical and sociodemographic epidemiology information on intussusception in children including the regional the variations. The network adopted a systematic site selection process. A common understanding among the investigators was ensured and transmitted to the research staffs for appropriate implementation. The effective coordination, data management and quality assurance measures ensured high quality data. Despite the efforts there were challenges related to the regulatory and administrative challenges, which affected the implementation timelines. The continuation of several sites and investigators in other studied demonstrated the strong collaboration and confidence coupled with capacity building.

Poor site selection can lead to delay in completion, rise in cost, protocol amendment, implementation variations or study failure (24). Studies have used different site selection processes: active hunting, peer referrals, inviting expression of interests, engaging research

For peer review only - http://bmjopen.bmj.com/site/about/guidelines.xhtml

organisations, etc. (25)(26)(27). Our organisation has been conducting multisite network studies (ranging from 5 to 84 partner institutions) over last 15 years. We have adopted variable combination of processes for the study site and investigators selection, which have evolved over time. For this study, we adopted a systematic site selection process, which appeared lengthy and required several rounds of interactions. The four-step process assisted in identifying the highly motivated investigators and study sites with desired capacity. The potential investigators who failed to return the questionnaire with desired information or participate in the interaction are unlikely to devote time for study conduct and supervision. The systematic selection process allowed reducing the potential sites to a manageable number of suitable sites that were further assessed through visit. While the process was lengthy, it allowed also to build common understanding and commitment for the study. These experiences were similar to some multicounty studies (25)(27)(28). The experiences from longer tenure clinical trial network (CTN) suggest that adoption of objective, standardised and systematic approach of site selection has better performance than an informal process. A CTN experimenting interventions for substance abuse moved from informal site selection process initially to five step process including identification of potential sites, site selection surveys, pilot simulation data abstraction, blinded review, site selection interviews or site visits (27). A CTN experimenting surgical interventions adopted five step site selection process including open call, site capacity survey and pilot simulation data abstraction, criteria based evaluation, telephonic interview and final assessment for selection (25). The steps adopted were comparable to the steps adopted in our study.

The procedural efforts and time taken for ethical approval from the site institutes are well known. A review observed that the study site ethics approval took from 5 to 798 days and the review process and contents varied widely (29). The ethics review process consumed sizable staff hours and budget, forcing timeline extension and budget shortfall (29)(30)(31). In our

For peer review only - http://bmjopen.bmj.com/site/about/guidelines.xhtml

study also the documentation and formats used for submission varied widely, apart from the study protocol, CRFs and consent forms. Some of the committees asked the investigators to present their protocol, while others didn't.

Although the investigators were responsible for the conduct of the study, the agreements with institutions assisted in implementation of the study. The involvement of external experts TAG as in study site selection and monitoring facilitated standardisation and quality assurance. The additional data retrieval and verification effort at the sites documented the protocol adherence and robustness of data collection.

This is the first documentation of systematic site selection process for a multisite network in India. The positive experience from this study encouraged adoption of similar systematic site selection and data quality assurance mechanism for subsequent two vaccine network studies in India.

There are some limitations in the current study and documentation. While many of the lessons may be generic and have relevance for most of the multisite studies, some of them may be relevant for India and the developing countries. The documentation of contractual and financial management challenges may be limited. The study was not a clinical trial and didn't involve any laboratory procedures. This was a descriptive study and had no comparison or control arm to compare the experience.

Conclusions

In conclusion, our experience with this systematic study site selection process was positive and satisfying. It used a four-step selection process using questionnaire, objective criteria for assessment, and interactions with the investigators and institution stakeholders with site visits to identify suitable sites with adequate capacity. The participation of motivated instigators, agreement with the institutions and contributory protocol and tool development facilitated

For peer review only - http://bmjopen.bmj.com/site/about/guidelines.xhtml

successful completion. Difficulties and delays in site initiation were primarily due to the regulatory and administrative approvals. The multistep site selection adopted is feasible and practical even in Indian context. The quality assurance processes also assisted in high quality data collection. We hope that the site selection and quality assurance processes would be informative for multisite studies in India and developing countries and appropriate adaptation or modifications may be needed as per the objectives and implementation protocol of the network studies.

For peer review only

For peer review only - http://bmjopen.bmj.com/site/about/guidelines.xhtml

Abbreviations

BCDC Brighton collaboration diagnostic criteria

CCU Central coordinating unit

CRF Case record form

EMR Electronic medical record

FCRA Foreign Contribution Regulation Act

HMSC Health Ministry Screening Committee

ICD International Classification of Diseases

RVV Rotavirus vaccines

TAG Technical Advisory Group

Usg Ultrasound

For peer review only

For peer review only - http://bmjopen.bmj.com/site/about/guidelines.xhtml

Acknowledgments

We acknowledge the support from Ministry of Health and Family Welfare, Government of India for undertaking the study. We are thankful to the hospital administrations and the clinicians at the study site institutes, who supported and facilitated undertaking of the study.

We highly value the technical guidance and inputs provided by the members of Technical Advisory Group: Satinder Aneja, Anju Seth and Archana Puri, Lady Hardinge Medical College, New Delhi; Ashok Patwari, Hamdard Institute of Medical Sciences & Research, New Delhi; Yogesh Kumar Sarin, Maulana Azad Medical College, New Delhi; Rakesh Aggarwal, Anshu Srivastava and Ujjal Poddar, Sanjay Gandhi Postgraduate Institute of Medical Sciences, Lucknow; Malathi Satyasekharan, Kanchi Kamakoti Chailds Trust Hospital, Chennai; Raju Sharma and Nirupam Madan, All India Institute of Medical Sciences, New Delhi; Jyoti Joshi and Deepak Polpakara, Immunization Technical Support Unit; Ministry of Health & Family Welfare, New Delhi; Umesh D. Parashar; Centers of Disease Control and Prevention, Atlanta, USA; Naveen Thacker, Child Health Foundation, Gandhigram; and Rashmi Arora, Indian Council of Medical Research, Ansari Nagar, New Delhi.

We acknowledge the contribution of the research staffs at The INCLEN Trust International: Harshpreet Kaur, Janvi Chaubey, Mrimmaya Das, Shweta Sharma and Vaibhav Jain.

We highly appreciate the efforts made by the research staffs at the study sites: Aarezo Bashir and Rafia; Sher-e-Kashmir Institute of Medical Sciences, Srinagar, Jammu & Kashmir; Prabha Shankar, Medanta-The Medicity Hospital, Gurgaon, Haryana; Anju Sharma; Maulana Azad Medical College, New Delhi; Anita Singh and Shubhranshu Srivastava, King George Medical University, Lucknow, Uttar Pradesh; Hemant Meena, Choithram Hospital, Indore, Madhya Pradesh; Pankaj Kumar and Shashi Kant; Indira Gandhi Institute of Medical Sciences, Patna, Bihar; Goutam Benia, IMS & SUM Medical College & Hospital, Bhubaneshwar, Odisha; Prasntajyoti Mohanty, SVP Post Graduate Institute of Paediatrics, Cuttack, Odisha;

For peer review only - http://bmjopen.bmj.com/site/about/guidelines.xhtml

Angshuman Chatterjee, Institute of Postgraduate Medical Education and Research & SSKM Hospital, Kolkata, West Bengal; S. Yamuna, Andhra Medical College, Vishakhapatnam, Andhra Pradesh; Srinidhi Sudan, Apollo Hospitals, Hyderabad, Telengana; Rajesh Francis, Apollo Hospitals, Chennai, Tamil Nadu; T. Easter Chandru, PSG Institute of Medical Sciences, Coimbatore, Tamil Nadu; Deepthy R, Julie and Anju Shivkumar, Government Medical College & SAT Hospital, Thiruvananthapuram, Kerala; Archit Vaidya, Grant Medical College & JJ Hospital, Mumbai, Maharashtra; Nimesh Chouksey, MP Shah Government Medical College, Jamnagar, Gujarat; Nidhi Singh, Fortis Escorts Hospital, Jaipur, Rajasthan; Mrinmoy Gohain, Gauhati Medical College, Guwahati, Assam; Arpita Bhattachrjee, Saugat Ghosh and Tanusmita Debnath, Agartala Government Medical College, Agartala, Tripura.

For peer review only

For peer review only - http://bmjopen.bmj.com/site/about/guidelines.xhtml

Declarations

Ethics approval and consent to participate: The study protocol was reviewed and approved by all the participating institutes. The list of ethics committees of the participating institutes include: The INCLEN Independent Ethics Committee, The INCLEN Trust International, New Delhi, India (Ref No: IIEC 23; Dated June 30, 2015); Institutional Ethics Committee, King George's Medical University, Lucknow, Uttar Pradesh, India (Ref no: 7951/Ethics/R.Cell-15; Dated December 4, 2015); Institutional Ethics Committee, MP Shah Government Medical College, Jamnagar, Gujarat, India (ref no: 01/46/2016; Dated January 6, 2016); Institutional Ethics Committee, Sher-I-Kashmir Institute of Medical Sciences, Srinagar, Jammu & Kashmir, India (Ref no: 42/2015; Dated October 29, 2015); Institutional Ethics Committee, Gauhati Medical College, Guwahati, Assam, India (Ref no: MC/02/2015/274; Dated May 30, 2016); Institutional Human Ethics Committee, PSG Institute of Medical Sciences, Coimbatore, Tamil Nadu, India (Ref no 15/294; Dated November 16, 2015); Institutional Ethics Committee, King George Hospital, Andhra Medical College, Vishakhapatnam, Andhra Pradesh, India (Dated October 26, 2015); Institutional Ethics Committee, Fortis Escorts Hospital, Jaipur, Rajasthan, India (Ref no: FEHJ/IEC/15/0023; Dated September 14, 2015); Institutional Ethics Committee, Grant Medical College & JJ Hospital, Mumbai, Maharashtra, India (Ref no: IEC/Pharm/288/15; Dated November 19, 2015); Institutional Ethics Committee, Government Medical College & SAT Hospital, Thiruvananthapuram, Kerala, India (Ref no: 06/05/2015/MCT; Dated December 9, 2015); Medanta Institutional Ethics Committee, Medanta-The Medicity, Gurgaon, Haryana, India (MICR 559/2015; Dated January 21, 2016); Institutional Ethics Committee, SCB Medical College, Cuttack, Odisha, India; Institutional Ethics Committee, IMS & SUM Medical College & Hospital, Bhubaneswar, Odisha, India (Ref no 210/5/10/2015; Dated October 14, 2015); Institutional Ethics Committee; Choithram Hospital and Research Centre, Indore, Madhya Pradesh, India (Ref no: EC/Oct/15/20; Dated

For peer review only

October 27, 2015); Institutional Ethics Committee, Agartala Government Medical College, Agartala, Tripura, India; Institutional Ethics Committee- Clinical Studies, Apollo Hospitals, Chennai, Tamil Nadu (Dated October 14, 2015); Institutional Ethics Committee, Maulana Azad Medical College, Delhi, India (Ref no: F.1/IEC/MAMC/50/4/2015/308; Dated November 20, 2015); Institutional Ethics Committee, Institute of Post Graduate Medical Education and Research, Kolkata, West Bengal, India (Ref no: Inst/IEC/2016/197; Dated March 1, 2016); Institutional Ethics Committee, Indira Gandhi Institute of Medical Sciences, Patna, Bihar, India (Ref no: 1256/Acad; Dated November 11, 2016); and Ethics Committee, Apollo Hospital, Hyderabad, Telengana, India ((Dated October 13, 2015). The interviews with stakeholders were done after obtaining written informed consent.

Consent for publication: Not applicable

Availability of data and materials: This manuscript reports the processes and experience from the multisite network research. All relevant data has been reported. All data is available with the investigators and can be provided by the corresponding author upon reasonable request.

Competing interests: The authors declare that there is no competing interests and conflict of interest.

Disclosure statement: None. There is no financial interest or benefit for the authors arisen from this project or its direct application.

Funding: This project was supported by the Bill and Melinda Gates Foundation, USA to The INCLEN Trust International (grant number OPP1116433). The funder or its representative had no role in the design of the study and collection, analysis, and interpretation of data and writing the manuscript.

Author contributions:

For peer review only - http://bmjopen.bmj.com/site/about/guidelines.xhtml

Study conceptualisation, study design, protocol development, training, data analysis, interpretation, coordination, monitoring and quality assurance: MKD and NKA.

Participant recruitment and data collection: AW, BRV, JIB, JKG, JM, KK, LB, LS, AMK, NM, PKJ, RS-1, RS-2, SKD, SJ, SKR, SS, VK, APD, AG, BBT, CJS, RPG, GNM, HT, JS, KL, ML, PP, RK, RS-3, SKA, SKS, SKG, SM, BAC, RPG, HKS, JK, NRS, PA, SSGM and SG.

Manuscript preparation: MKD, NKA and AR

All authors reviewed, provided critical input and approved the final version. The content represents the views of the authors alone and do not necessarily represent the official positions of their organizations.

For peer review only

For peer review only - http://bmjopen.bmj.com/site/about/guidelines.xhtml

References

1. Bines JE, Kohl KS, Forster J, Zanardi LR, Davis RL, Hansen J, et al. Acute intussusception in infants and children as an adverse event following immunization: case definition and guidelines of data collection, analysis, and presentation. Vaccine. 2004;22(5–6):569–74.
2. World Health Organisation. Post-marketing surveillance of rotavirus vaccine safety [Internet]. 2017 [cited 2017 Sep 18]. Available from: http://apps.who.int/iris/bitstream/10665/70017/1/WHO_IVB_09.01_eng.pdf
3. Tate JE, Mwenda JM, Armah G, Jani B, Omore R, Ademe A, et al. Evaluation of Intussusception after Monovalent Rotavirus Vaccination in Africa. N Engl J Med. 2018;378(16):1521–8.
4. Groome MJ, Tate JE, Arnold M, Chitnis M, Cox S, de Vos C, et al. Evaluation of Intussusception After Oral Monovalent Rotavirus Vaccination in South Africa. Clin Infect Dis Off Publ Infect Dis Soc Am. 2020;70(8):1606–12.
5. Patel MM, López-Collada VR, Bulhões MM, De Oliveira LH, Márquez AB, Flannery B, et al. Intussusception Risk and Health Benefits of Rotavirus Vaccination in Mexico and Brazil. N Engl J Med. 2011;364(24):2283–92.
6. Velázquez FR, Colindres RE, Grajales C, Hernández MT, Mercadillo MG, Torres FJ, et al. Postmarketing surveillance of intussusception following mass introduction of the attenuated human rotavirus vaccine in Mexico. Pediatr Infect Dis J. 2012;31(7):736–44.
7. Carlin JB, Macartney KK, Lee KJ, Quinn HE, Buttery J, Lopert R, et al. Intussusception risk and disease prevention associated with rotavirus vaccines in Australia's National Immunization Program. Clin Infect Dis Off Publ Infect Dis Soc Am. 2013;57(10):1427–34.

For peer review only

For peer review only - http://bmjopen.bmj.com/site/about/guidelines.xhtml

8. Yih WK, Lieu TA, Kulldorff M, Martin D, McMahill-Walraven CN, Platt R, et al. Intussusception risk after rotavirus vaccination in U.S. infants. N Engl J Med. 2014;370(6):503–12.

9. Weintraub ES, Baggs J, Duffy J, Vellozzi C, Belongia EA, Irving S, et al. Risk of intussusception after monovalent rotavirus vaccination. N Engl J Med. 2014;370(6):513–9.

10. Stowe J, Andrews N, Ladhani S, Miller E. The risk of intussusception following monovalent rotavirus vaccination in England: A self-controlled case-series evaluation. Vaccine. 2016 12;34(32):3684–9.

11. Pérez-Vilar S, Díez-Domingo J, Puig-Barberà J, Gil-Prieto R, Romio S. Intussusception following rotavirus vaccination in the Valencia Region, Spain. Hum Vaccines Immunother. 2015;11(7):1848–52.

12. Koch J, Harder T, von Kries R, Wichmann O. Risk of Intussusception After Rotavirus Vaccination. Dtsch Arzteblatt Int. 2017;114(15):255–62.

13. Jiang J, Jiang B, Parashar U, Nguyen T, Bines J, Patel MM. Childhood intussusception: a literature review. PloS One. 2013;8(7):e68482.

14. Bahl R, Saxena M, Bhandari N, Taneja S, Mathur M, Parashar UD, et al. Population-Based Incidence of Intussusception and a Case-Control Study to Examine the Association of Intussusception with Natural Rotavirus Infection among Indian Children. J Infect Dis. 2009;200(s1):S277–81.

15. Jehangir S, John J, Rajkumar S, Mani B, Srinivasan R, Kang G. Intussusception in southern India: Comparison of retrospective analysis and active surveillance. Vaccine. 2014;32:A99–103.

16. Ministry of Health and Family Welfare, Government of India. Meeting Minutes: Meeting of the National Technical Advisory Group on Immunization (India) [Internet]. Ministry of

For peer review only - http://bmjopen.bmj.com/site/about/guidelines.xhtml

Health & Family Welfare Government of India; 2014 [cited 2020 Nov 3]. Available from: https://www.nitag-resource.org/media-center/document/2220-minutes-of-the-meeting-of-ntagi-held-in-2014

17. Coates EC, Mann-Salinas EA, Caldwell NW, Chung KK. Challenges Associated with Managing a Multicenter Clinical Trial in Severe Burns. J Burn Care Res. 2020;41(3):681–9.

18. Hagen NA, Stiles CR, Biondo PD, Cummings GG, Fainsinger RL, Moulin DE, et al. Establishing a multicentre clinical research network: lessons learned. Curr Oncol Tor Ont. 2011;18(5):e243-249.

19. Dewa CS, Durbin J, Wasylenki D, Ochocka J, Eastabrook S, Boydell KM, et al. Considering a multisite study? How to take the leap and have a soft landing. J Community Psychol. 2002;30(2):173–87.

20. Forjuoh SN. Challenges Associated with Multi-institutional Multi-site Clinical Trial Collaborations: Lessons from a Diabetes Self-Management Interventions Study in Primary Care. J Clin Trials [Internet]. 2015 [cited 2020 Nov 4];05(03). Available from: http://www.omicsgroup.org/journals/challenges-associated-with-multiinstitutional-multisite-clinical-trial-collaborations-lessons-from-a-diabetes-selfmanagement-interventions-study-in-primary-care-2167-0870-1000219.php?aid=55032

21. Das M, Arora N, Bonhoeffer J, Zuber P, Maure C. Intussusception in Young Children: Protocol for Multisite Hospital Sentinel Surveillance in India. Methods Protoc. 201822;1(2):11.

22. Das MK, Arora NK, Gupta B, Sharan A, Kameswari K, Padmalatha P, et al. Intussusception in children aged under two years in India: Retrospective surveillance at nineteen tertiary care hospitals. Vaccine. 2020;38(43):6849–57.

For peer review only

23. The INCLEN Intussusception Surveillance Network Study Group. Prospective surveillance for intussusception in Indian children aged under two years at nineteen tertiary care hospitals. BMC Pediatr. 2020;20(1):413.

24. Fogel DB. Factors associated with clinical trials that fail and opportunities for improving the likelihood of success: A review. Contemp Clin Trials Commun. 2018 Sep;11:156–64.

25. Hurtado-Chong A, Joeris A, Hess D, Blauth M. Improving site selection in clinical studies: a standardised, objective, multistep method and first experience results. BMJ Open. 2017;7(7):e014796.

26. Gheorghiade M, Vaduganathan M, Greene SJ, Mentz RJ, Adams KF, Anker SD, et al. Site selection in global clinical trials in patients hospitalized for heart failure: perceived problems and potential solutions. Heart Fail Rev. 2014;19(2):135–52.

27. Potter JS, Donovan DM, Weiss RD, Gardin J, Lindblad R, Wakim P, et al. Site Selection in Community-Based Clinical Trials for Substance Use Disorders: Strategies for Effective Site Selection. Am J Drug Alcohol Abuse. 2011;37(5):400–7.

28. Farquharson K, Murphy KA. Ten Steps to Conducting a Large, Multi-Site, Longitudinal Investigation of Language and Reading in Young Children. Front Psychol [Internet]. 2016 Mar 30 [cited 2020 Nov 4];7. Available from: http://journal.frontiersin.org/Article/10.3389/fpsyg.2016.00419/abstract

29. Greene SM, Geiger AM. A review finds that multicenter studies face substantial challenges but strategies exist to achieve Institutional Review Board approval. J Clin Epidemiol. 2006;59(8):784–90.

30. Green LA, Lowery JC, Kowalski CP, Wyszewianski L. Impact of institutional review board practice variation on observational health services research. Health Serv Res. 2006;41(1):214–30.

For peer review only - http://bmjopen.bmj.com/site/about/guidelines.xhtml

31. Maskell NA, Jones EL, Davies RJO, BTS/MRC MIST steering committee. Variations in experience in obtaining local ethical approval for participation in a multi-centre study. QJM Mon J Assoc Physicians. 2003;96(4):305–7.

For peer review only

For peer review only - http://bmjopen.bmj.com/site/about/guidelines.xhtml

Figures

Figure 1: The steps of the site selection process

Note: TAG: Technical Advisory Group

Figure 2: The data collection and management flow for prospective surveillance

Abbreviations: BCDC: Brighton collaboration diagnostic criteria; CCU: Central coordinating unit; CRF: case record form; TAG: Technical Advisory Group; Usg: Ultrasound

Figure 3: The data collection and management flow for retrospective surveillance

Abbreviations: BCDC: Brighton collaboration diagnostic criteria; CCU: Central coordinating unit; CRF: case record form; ICD: International classification of diseases; TAG: Technical Advisory Group; Usg: Ultrasound

Figure 4: The geographic distribution of the network study sites

Note: The image was created for the research project by the investigators.

For peer review only

Supplementary Documents

Supplementary document 1: Potential Study Site Institution Information

Supplementary document 2: Potential Study Site Institution Assessment Report (Site Visit)

Supplementary document 3: The ICD codes for retrieval of the cases from the medical records section

For peer review only

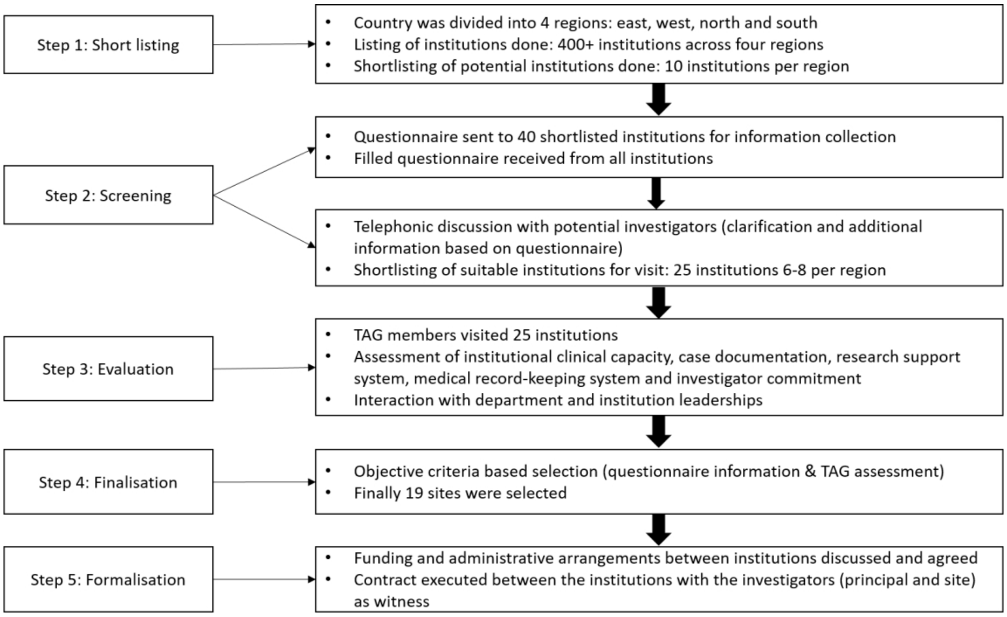

Figure 1: The steps of the site selection process

427x264mm (96 x 96 DPI)

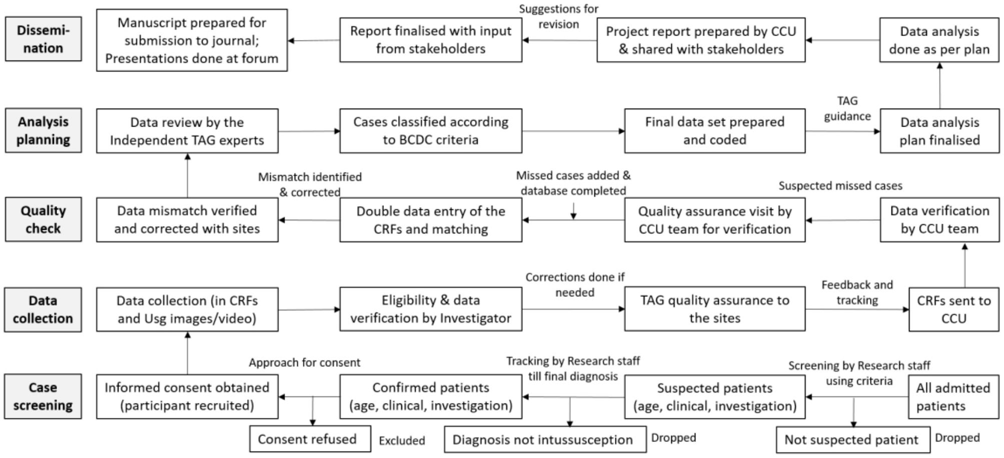

Figure 2: The data collection and management flow for prospective surveillance

516x238mm (96 x 96 DPI)

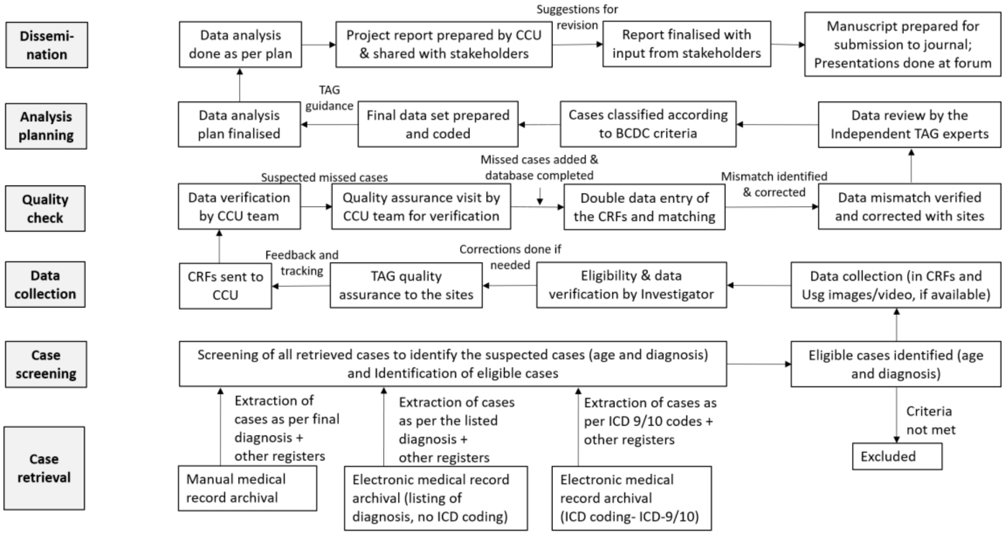

Figure 3: The data collection and management flow for retrospective surveillance

502x270mm (96 x 96 DPI)

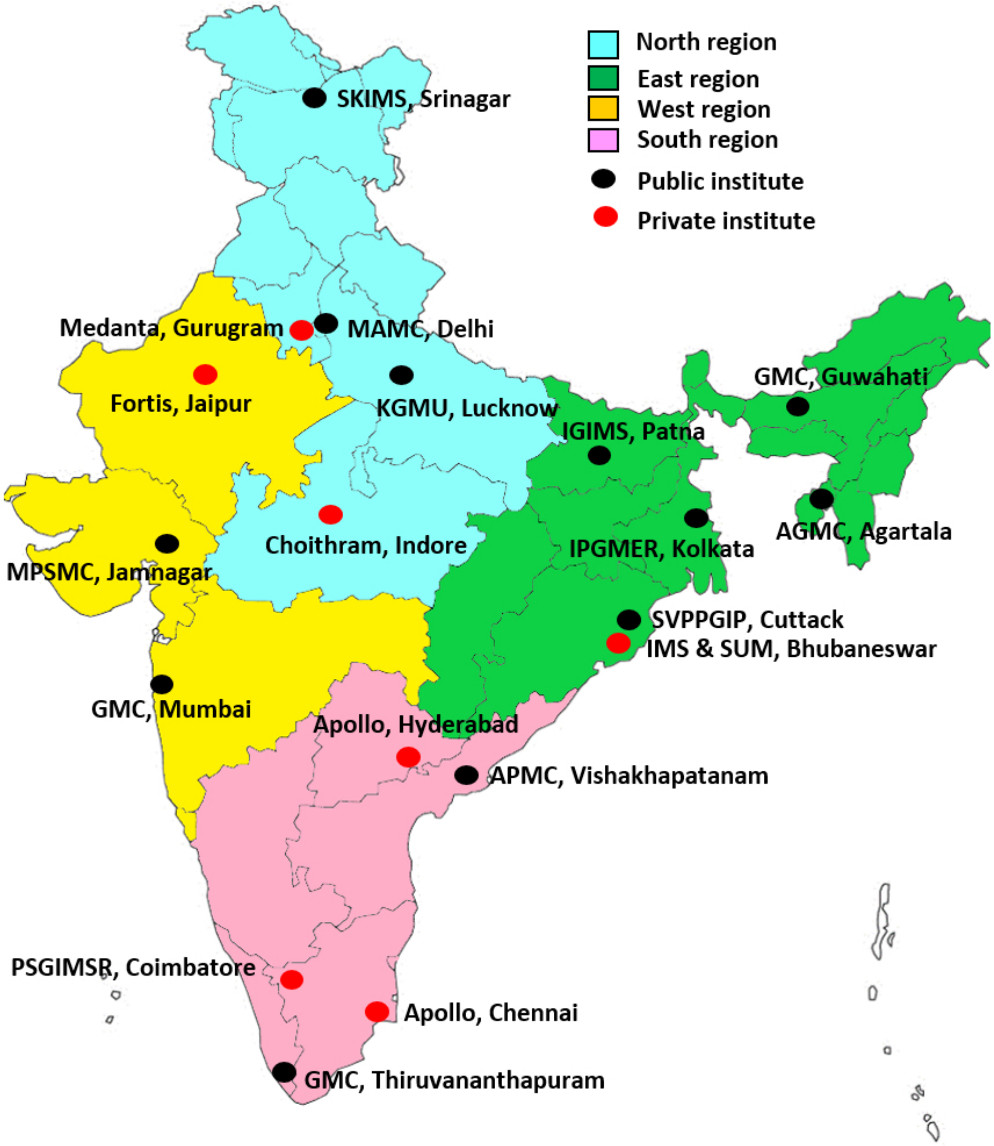

Figure 4: The geographic distribution of the network study sites

228x263mm (96 x 96 DPI)

Intussusception in Infants: Multisite Hospital Based Surveillance in India
Potential Study Site Institution Information

**Institution**	
**Place**	
**State**	

Dear Sir/ Madam,
We are undertaking a study for documentation of intussusception in children under-two years of age at multiple sites in India. This study aims collection of retrospective data over last five-years and prospective data over 18 months on intussusception in children.
As this study involves data retrieval for retrospective data, access to medical records is very critical. Additionally, the access to /availability of clinical case record and radiological images would be very essential for making confirmation of diagnosis.
We request you to provide the following information on facilities and infrastructure at your institute/hospital focusing on diagnosis and management of the intussusception in infants. We highly appreciate your collaboration in answering the following questions and sending them to manoj@inclentrust.org.

A. General information (Please fill the response)
(Please provide information about the concerned departments in the hospital)

Sl no	Item/ Question	Pediatrics	Pediatric surgery	Pediatric gastroenterology	Radiology
1	Number of beds				
2	Number of Specialists (Teaching faculty/ non-teaching specialists)				
3	Academic Residents (MD/DNB/MCH/Fellows)				
4	Non-academic Residents				
5	Number of children (< 5 years) with intussusception admitted in last 1 year				
6	Number of infants (< 1 year) with intussusception admitted in last 1 year				

For peer review only - http://bmjopen.bmj.com/site/about/guidelines.xhtml

B. Clinical services

Sl no	Questions/ Items<br>*(Please mark √in appropriate box; mark as many apply)*	Response Yes	Response No
**1**	**Availability of round the clock services**		
1.1	Is the pediatric service available round the clock in the hospital?		
1.2	Is the pediatric surgery service available round the clock in the hospital?		
1.3	Is the surgery service available round the clock in the hospital? (If Pediatric Surgery is not available)		
1.4	Is the Emergency Service available round the clock in the hospital?		
1.5	Is the Emergency Registration Service separate from the Central Admission Registration?		
**2**	**What is the system of Central Admission Registration?**		
2.1	Computerized/ electronic admission registration		
2.2	Manual admission registration		
2.3	Any other method<br>(Specify ………………………………………………………….)		
**3**	**What is the system of Emergency Registration System?**		
3.1	Computerized/ electronic admission registration		
3.2	Manual admission registration		
3.3	Any other method<br>(Specify ………………………………………………………….)		
**4**	**Does the institution have a Centralized Medical Record Index/ Master Patient Index/ Centralized Admission Register?**		
**5**	**If yes, how is it maintained?**		
5.1	Maintained department wise		
5.2	Maintained all departments combined		
5.3	Any other method		
**6**	**Does your hospital give Rotavirus vaccination to the infants?**		
**6.1**	If yes, is the Rotavirus vaccine available in the hospital?		
**6.2**	If yes, is the Rotavirus vaccine purchased by parents from outside?		

For peer review only

C. Diagnostic services

Sl no	Questions/ Items	Response
**1**	**Availability of the services**	
1.1	How many ultrasound machines are available in the hospital?	
1.2	What is the timing of availability of the ultrasound services?	
2	**Is there a ultrasound facility/ service available in the emergency department/ casualty?**	
**3**	**Who does the ultrasound in emergency hours?** (outside the routine hours)	
3.1	Qualified Radiologist	
3.2	Resident Radiology	
3.3	Any other person (specify ………………………………………..)	
**4**	**How may of the ultrasound machines have facility for digital image storage/ archival?**	
4.1	If yes, for how long the digital images of the ultrasound tests are stored/ archived?	
4.2	Are these digital images retrievable?	
**5**	**What is the reporting system for ultrasound tests?**	
5.1	Only a written report is given	
5.2	Only the film is given	
5.3	Written report and film	
5.4	Images in form of CD/ any storage device	

D. Management of Intussusception cases

Sl no	Questions/ Items<br>*(Please mark √in appropriate box; mark as many apply)*	Response	
		Yes	No
**1**	**Surgery for intussusception**		
1.1	Does the pediatric surgery/ surgery department conduct surgery for intussusception in children?		
1.2	Does the pediatric surgery/ surgery department conduct surgery for intussusception in infants (aged < 1 years)?		
1.3	If No, where are the children/infants are usually referred? (Name of the hospital and place)		
2	**Conservative management of intussusception**		
2.1	Do the children with intussusception are managed by barium enema/ air contrast enema at this hospital?		
2.2	If yes, who does this?		
2.2.1	Radiologist		
2.2.2	Pediatric surgeon		
2.2.3	Pediatrician/ pediatric gastroenterologist		
2.2.4	Any other (specify……………………………………………..)		

For peer review only - http://bmjopen.bmj.com/site/about/guidelines.xhtml

E. Medical Record Archival System

The project requires screening/review of the hospitalized infants in last five years. Thus listing and retrieval of medical records are very essential. The following questions/items focus on the admission, medical record archival and retrieval process. This will allow planning the case record tracking and retrieval at the institution.

Sl no	Questions/ Items (Please mark √in appropriate box; mark as many apply)	Response Yes	No	
**1**	**Is the admission registration for hospital is centralized?**			***If NO, skip to Q 3***
**2**	**If yes, what is the system of admission registration?**			
2.1	Computerized/ electronic admission registration			
2.2	Manual admission registration			
2.3	Any other method (Specify …………………………………...)			
**3**	**Does the institution have a centralized medical record index/ Master Patient Index/ Centralized Admission Register?**			***If NO, skip to Q 5***
**4**	**If yes, what all information can be retrieved from the centralized medical record index/ Master Patient Index/ centralized admission register?**			
4.1	Admission number			
4.2	Date of admission			
4.3	Department of admission			
4.4	Name			
4.5	Age			
4.6	Sex			
4.6	Diagnosis at admission			
4.7	Final diagnosis			
4.8	Outcome (discharge/ death/LAMA)			
4.9	Date of outcome			
**5**	**How the case-sheets (hard copies) are stored/ archived in your hospital/institution for in-patients cases?**			
5.1	Admission number wise			
5.2	Department wise			
5.3	Date of admission wise			
5.4	Date of discharge wise			
5.5	Any other method (specify……………………………………)			
**6**	**How are the case-sheets (hard copies) of in-patient cases indexed/ listed?**			
6.1	Computerized/ electronic listing			
6.2	Manual listing			
6.3	Any other method (specify……………..……………………..)			
6.4	Not listed			

For peer review only - http://bmjopen.bmj.com/site/about/guidelines.xhtml

7	**If computerized/ electronic indexing/ listing is done, since when (year) it is being done?**			Year
8	**Are the death cases archived separately?**			
8	**Does the institute summarize the medical records of hospitalized patients periodically according to any classification system** (ICD 10 or any other)?			
9	**Does the institute/hospital have case-records of hospitalized children for last five years readily accessible?**			
10	**Please indicate about the listing/recording the information about hospitalized children under Pediatrics Department?**			
10.1	Availability of list/ index of hospitalized children (Like in the list of admitted children maintained by Nurses/ward in-charge)			
11.2	Discharge sheets/ summary of children (copy of discharge sheet maintained by Nurses/ Residents/ ward in-charge)			
11	**Please indicate about the listing/recording of the information about children hospitalized under Pediatrics Surgery Department?**			
11.1	Availability of list/index of hospitalized children			
11.2	Operation Theatre Register			
11.3	Discharge sheets/ summary of children			
12	**Does the radiology department maintain a register/ listing of patients undergoing investigation?**			
13	**What all information can be retrieved from the list/recording?**			
13.1	Admission number			
13.2	Date of investigation			
13.3	Name			
13.4	Age			
13.5	Sex			
13.6	Finding/ Diagnosis			

F. Institute authority approvals

The project requires access to the case-sheets of children with intussusception admitted to your hospital and collection of clinical details about them. Please inform about the approvals from the institute authority(ies) to be obtained for the same.

Sl no	Questions/ Items	
1	**What all permissions from institute/department authorities are needed and the time needed for the same?**	
	Authority	Time needed
1.1		
1.2		
1.3		
1.4		

For peer review only - http://bmjopen.bmj.com/site/about/guidelines.xhtml

**2**	**How frequently (in months) the institute ethics committee meets?**	Monthly
**3**	**What all challenges do you anticipate in this process?**	
**4**	**How all these challenges can be addressed?**	
**5**	**Any other specific comments/ suggestions?**	

G. Investigating Team Members

Please inform about the Investigating team members from the institute along with their affiliations and contact information.

	Name	Department	Designation	Address details (including email and phone)
1				
2				
3				
4				

We thank you for your support and time taken to complete the required information.

Signature Name

Date: ……/……../…………………..

For any further clarification or information, you may contact Dr Manoj Das at manoj@inclentrust.org or (M) 9810203768; (O) 011-47730000

For peer review only - http://bmjopen.bmj.com/site/about/guidelines.xhtml

Intussusception in Infants: Multisite Hospital Based Surveillance in India
Potential Study Site Institution Assessment Report (TAG Site Visit)

**Institution**	
**Place**	
**State**	
**Date of visit**	

Suggestion for the Assessor

Please meet the potential investigators from Pediatrics, Pediatric Surgery, Radiology and In-charge of Medical Record Section to obtain the desired information to be captured in this tool. This is critical to select the suitable study sites to enable quality data on intussusception cases in infants. We highly appreciate your collaboration in providing your responses to the following questions and sending them to manoj@inclentrust.org.

A. General information

(Please fill the response)

(Please document the information about the hospital and concerned departments)

Sl no	Item/ Question	Response
1	Total number of beds	
2	Is the Emergency Service available round the clock? (Yes/No)	
3	Is the pediatric service available round the clock? (Yes/No)	
4	Is the pediatric surgery service available round the clock? (Yes/No)	
5	Is the Radiology service available round the clock? (Yes/No)	

B. Pediatrics Department

(Please document the information about the Pediatrics Department)

Sl no	Item/ Question	Response
1	Total number of units in the Department	
2	Total number of in-patient beds	
3	Total number of ICU/PICU beds	
4	Number of faculty/ specialists	
5	Number of senior residents	
6	Number of academic junior residents/ DNB students	
7	Number of non-academic residents	

For peer review only - http://bmjopen.bmj.com/site/about/guidelines.xhtml

8	Is there a pediatric gastroenterology unit/department in the hospital? (Yes/No)	
9	Does any (or >1) pediatrician specializes or focuses on pediatric gastroenterology in the hospital? (Yes/No)	
10	Is there a pediatric gastroenterology OPD/ clinic in the hospital? (Yes/No)	
11	Is there a diarrhea ward/ unit in the hospital for children? (Yes/No)	
12	How are the children with following conditions managed?	Write
12.1	Diarrhoea	
12.2	Blood in stool	
12.3	Infants/young children with pain abdomen or excessive crying	
12.4	Infants/young children with vomiting	
12.5	Infants/young children with surgical abdomen	
12.6	Infants/young children with diagnosis of intussusception	
13	What is usually done, if an infant/ child comes with an ultrasound suggesting intussusception?	Write
13.1	Ultrasound repeated	
13.2	Any additional tests done	
13.3	Admission and evaluation<br>a. Admitted to pediatrics and then transferred to pediatric Surgery/ Surgery department OR<br>b. Directly referred to pediatric Surgery/ Surgery department)	
14	How are the children with intussusception managed? (ask for barium/ air contrast enema)	
15	How are the infants with intussusception managed? (ask for barium/ air contrast enema)	

For peer review only

16	How many patients aged < 5 years with of intussusception were seen in last one year in this department? (approximate number)	
17	How many infants (< 1 years) with of intussusception were seen in last one year in this department? (approximate number)	
18	How many were managed by this department? (approximate number)	
19	Was any of these patients with intussusception were referred to any other hospital? (approximate number)	
20	If yes, what were the most likely reasons?	
21	For the patients getting discharged, is there a copy of the discharge sheet kept in the ward/ in the department? (Yes/No)	
22	If Yes, where is the file/ archival is made?	
23	Please review some case records of admitted infants to document the following.	
23.1	Does the hospital use any structured case record/ follow specific format for clinical history recording? (Yes/No)	
23.2	Is the immunization history recorded in the clinical history? (Yes/No)	
23.3	Is the immunization history recorded with age at vaccination? (Yes/No)	
24	Is the Rotavirus vaccine given at the hospital?	
25	Any other specific observations/ comments	

For peer review only - http://bmjopen.bmj.com/site/about/guidelines.xhtml

C. Pediatric Surgery/ Surgery Department

(Please document the information about the Pediatric Surgery Department.)
(Please ask for the Surgery Department, if there is no Pediatric Surgery Department.)

Sl no	Item/ Question	Response
1	Is there a Pediatric Surgery Department in the hospital? (Yes/ No)	
2	Total number of units in the Department	
3	Total number of in-patient beds	
4	Total number of ICU/PS-ICU beds	
5	Number of faculty/ specialists	
6	Number of senior residents	
7	Number of academic junior residents/ DNB students	
8	Number of non-academic residents	
9	If there is no Pediatric Surgeon, how many Surgeons are conducting surgery in children?	
10	If there is no Pediatric Surgeon, how many Surgeons are conducting surgery in infants?	
11	What is usually done, if an infant/ child comes with an ultrasound suggesting intussusception?	Write
11.1	Ultrasound repeated	
11.2	Any additional tests done	
11.3	Admission and evaluation<br>a. Admitted to pediatrics and then transferred to pediatric Surgery/ Surgery department OR<br>b. Directly referred to pediatric Surgery/ Surgery department)	
12	How are the children with intussusception managed? (ask for barium/ air contrast enema)	
13	How are the infants with intussusception managed? (ask for barium/ air contrast enema)	
14	How many patients aged < 5 years with of intussusception were seen in last one year in this department? (approximate number)	
15	How many infants (< 1 years) with of intussusception were seen in last one year in this department? (approximate number)	

For peer review only - http://bmjopen.bmj.com/site/about/guidelines.xhtml

16	How many were managed by this department? (approximate number)	
17	Was any of these patients with intussusception were referred to any other hospital? (approximate number)	
18	If yes, what were the most likely reasons?	
19	Is there a register for listing operated cases? (Yes, No)	
20	If Yes, please check mark the components mentioned in the register.<br>Name: Yes No Sex: Yes No<br>Patient ID: Yes No Age: Yes No<br>Diagnosis: Yes No Date of surgery: Yes No<br>Time taken: Yes No Procedure done: Yes No<br>Outcome: Yes No Surgeon: Yes No<br>Any other:	
21	Is there a register for listing children undergone non-operative intervention (like air-contrast/barium enema) (Yes, No)	
22	If Yes, please check mark the components mentioned in the register.<br>Name: Yes No Sex: Yes No<br>Patient ID: Yes No Age: Yes No<br>Diagnosis: Yes No Date of surgery: Yes No<br>Time taken: Yes No Procedure done: Yes No<br>Outcome: Yes No Surgeon: Yes No<br>Any other:	
23	For the patients getting discharged, is there a copy of the discharge sheet kept in the ward/ in the department? (Yes, No)	
24	If Yes, where is the file/ archival is made?	
25	Please review some case records of admitted infants to document the following.	
25.1	Does the hospital use any structured case record/ follow specific format for clinical history recording? (Yes/No)	
25.2	Is the immunization history recorded in the clinical history? (Yes/No)	
25.3	Is the immunization history recorded with age at vaccination? (Yes/No)	
26	Any other specific observations/ comments	

For peer review only - http://bmjopen.bmj.com/site/about/guidelines.xhtml

D. Anesthesia Department and Operation Theatre

(Please document the information about the Anesthesia Department.)

Sl no	Item/ Question	Response
1	Does the anesthetist(s) in the hospital give anesthesia to children and infants? (Yes/ No)	
2	Is the OT well equipped to handle the surgery and anesthesia of infants? (Yes/ No)	
3	Is there a register for listing operated cases? (Yes, No)	
4	If Yes, please check mark the components mentioned in the register.<br>Name: Yes No Sex: Yes No<br>Patient ID: Yes No Age: Yes No<br>Diagnosis: Yes No Date of surgery: Yes No<br>Time taken: Yes No Procedure done: Yes No<br>Outcome: Yes No Surgeon: Yes No<br>Any other:	
5	Any other specific observations/ comments	

E. Radiology Department

(Please document the information about the Radiology Department.)

Slno	Item/ Question	Response
1	Total number of Ultrasound machines	
1.1	How may Ultrasound machines have option for digital image saving?	
1.2	Is the digital image saving a routine practice? (Yes/ No)	
1.3	If Yes, for how many days usually the digital images are saved?	
2	Total number of X-ray machines (for barium enema/air contrast enema)	
3	Number of fluoroscopy machine (for barium enema/air contrast enema)	
4	Number of faculty/ specialists	
5	Number of senior residents	
6	Number of academic junior residents/ DNB students	
7	What is the usual timing for ultrasound in the hospital?	From<br>To

For peer review only - http://bmjopen.bmj.com/site/about/guidelines.xhtml

8	What is the arrangement for doing ultrasound beyond routine hours/ nights?	
9	Does the machine(s) used for ultrasound in emergency have facility for digital image saving?	
10	How the reports of ultrasound are given to patients?	
10.1	Only paper report (no film/ image)	
10.2	Paper report with printed paper image (no films)	
10.3	Paper report with films	
10.4	Paper report with CD with digital images	
10.5	Paper report and others available on request/ payment	
10.6	How the reports of barium enema are given to patients?	
10.7	Only paper report (no film/ image)	
10.8	Paper report with films	
10.9	Paper report with CD with digital images	
10.10	Paper report and others (film/CD) on request/ payment	
11	How many patients aged < 5 years with of intussusception were seen in last one year in this department? (approximate number)	
12	How many infants (< 1 years) with of intussusception were seen in last one year in this department? (approximate number)	
13	Do you manage the infants/ children with intussusception by barium/ air contrast enema? (Yes/ No)	
14	If Yes, when was the last such case managed?	
15	Does the department keep a copy of the ultrasound report for record? (Yes, No)	
16	If Yes, where is the file/ archival is made?	
17	Does the radiology department maintain a register/ listing of patients undergoing investigation (ultrasound/ barium)?	
18	What all information can be retrieved from the list/recording?	
18.1	Admission number	
18.2	Date of investigation	
18.3	Name	
18.4	Age	
18.5	Sex	
18.6	Findings/ Diagnosis	
19	Any other specific observation/ comment	

For peer review only - http://bmjopen.bmj.com/site/about/guidelines.xhtml

F. Case Registration System
(Please document about the case registration system of the hospital for in-patient cases)

Sl no	Questions/ Items	Response (Yes/No)
1	What is the system of Central In-patient Admission Registration?	
1.1	Computerized/ electronic admission registration	
1.2	Manual admission registration	
1.3	Any other method<br>(Specify ………………………………………………………….)	
2	If the Registration system is manual, how is the Registration Index maintained?	
2.1	Maintained department wise	
2.2	Maintained all departments combined	
2.3	Any other method	
3	Is the Central In-patient Admission Registration system available round the clock?	
4	Is the Emergency Case Registration System separate from the Central in-patient registration?	
5	If Yes, What is the system of Emergency Registration System?	
5.1	Computerized/ electronic admission registration	
5.2	Manual admission registration	
5.3	Any other method<br>(Specify ………………………………………………………….)	
6	What all information can be retrieved from the Central In-patient Admission Registration Information? (Yes/No)	
6.1	Admission number	
6.2	Date of admission	
6.3	Department of admission	
6.4	Name	
6.5	Age	
6.6	Sex	
6.7	Diagnosis at admission	
6.8	Final diagnosis	
6.9	Outcome (discharge/ death/LAMA)	
6.10	Date of outcome	
7	Any specific observation/ comments	

For peer review only

G. Medical Record Archival System

The project requires screening/review of the hospitalized infants in last five years. Thus listing and retrieval of medical records are very essential. The following questions/items focus on the admission, medical record archival and retrieval process. This will allow planning the case record tracking and retrieval at the institution.

Sl no	Questions/ Items	Response (Yes/No)
1	Does your hospital have a Medical records Department/ Division?	
2	How frequently the in-patient case-sheets (hard copies) are sent to Medical Records Department/ Division from the departments?	
3	How the in-patient case-sheets (hard copies) are stored/ archived in your hospital/institution?	
3.1	Admission number wise	
3.2	Department wise	
3.3	Date of admission wise	
3.4	Date of discharge wise	
3.5	Any other method of organization (specify…………………………………………………)	
4	How are the in-patient case-sheets (hard copies) indexed/ listed?	
4.1	Computerized/ electronic listing	
4.2	Manual listing	
4.3	Any other method (specify……………………………………………………..)	
4.4	Not listed	
5	If computerized/ electronic indexing/ listing is done, since when (year) it is being done?	Year
6	Does the institute summarize the medical records of hospitalized patients periodically according to any classification system (ICD 10 or any other)?	
7	If yes, who does the classification labeling of the cases?	
8	How frequently the classification labeling of the cases is done?	
9	Is there any policy by the hospital regarding duration of archival/ storage of the in-patient case-sheets (hard copies)?	
10	If Yes, for how many years the hard copies are stored?	

For peer review only - http://bmjopen.bmj.com/site/about/guidelines.xhtml

11	Is there any effort for digital storage of the in-patient case-sheets?	
12	If yes, since when? (year)	
13	What is the process of retrieval of the case records from the Medical Records Department? (please describe focusing on the following)<br>a. The process of application:<br>b. Time needed for approval<br>c. Time needed for retrieval and receipt of the case records<br>d. Any limitation on the number of case records issued in one go	
14	How can we retrieve the list of children admitted with diarrhea to the hospital in month of June 2010? (please ask the concerned official for demonstrating the same)	
15	If retrieved, please document the number of children admitted with diarrhea in the month of June 2010.	
16	Any other challenge in retrieval of the in-patient case-sheets (hard copies) for last five years for the pediatrics and pediatric surgery departments?	
17	Any specific observation/ comments	

For peer review only - http://bmjopen.bmj.com/site/about/guidelines.xhtml

H. Institute authority and regulatory approvals

The project requires retrieval of the case-sheets of children with intussusception admitted to your hospital and collection of clinical details about them. Please inform about the approvals from the institute authority(ies) to be obtained for the same.

Sl no	Questions/ Items	
**1**	**What all permissions from institute/department authorities are needed and the time needed for the same?**	
	Authority	Time needed
1.1		
1.2		
1.3		
1.4		
1.5		
**2**	**How frequently (in months) the institute ethics committee meets?**	
**3**	**Meeting with Department Head and Faculty Colleagues**<br>**What is your assessment about the reputation of the potential investigator and support anticipated from the department?**	
**4**	**Meeting with Institute Head or Research Head**<br>**What is your assessment about the research environment in the institute and facilitations anticipated from the department?**	
**5**	**What all challenges do you anticipate in this process?**	

For peer review only

6	**How all these challenges can be addressed/mitigated?**
**7**	**Any other specific comments/ suggestions?**

I. Investigating Team Members

Please inform about the Investigating team members from the institute along with their affiliations and contact information.

	Department	Name	Designation	Address details (including email and phone)
1	Pediatrics			
2	Pediatric Surgery			
3	Radiology			
4	Medical Records			
5	Any other			

We thank you for your support and time taken to complete the required information.

Any other observations/ comments

Signature Name

Date: ……/……../…………………..

For peer review only - http://bmjopen.bmj.com/site/about/guidelines.xhtml

Supplementary document 3:

The International Classification of Diseases (ICD) codes for retrieval of the cases from the medical records section

Clinical conditions considered as suspected cases	Codes	
	ICD 10	ICD 9
Intussusception	K56.1	560.0
Volvulus	K56.2	560.2
Gallstone ileus	K56.3	560.31
Other impaction of intestine	K56.4	560.30
Intestinal adhesions with obstruction	K56.5	560.81
Other and unspecified intestinal obstruction	K56.6	560.9
Ileus, unspecified	K56.7	560.1
Paralytic ileus	K56.0	

ICD: International Classification of Diseases

For peer review only

Title: Experience of establishing and coordinating a nationwide network for bi-directional intussusception surveillance in India: Lessons for multisite research studies

STROBE Statement

**Items**	**Item No**	**Recommendation**	**Page No**
**Title and abstract**	1	(*a*) Indicate the study's design with a commonly used term in the title or the abstract	1
		(*b*) Provide in the abstract an informative and balanced summary of what was done and what was found	9-10
**Introduction**			
Background/rationale	2	Explain the scientific background and rationale for the investigation being reported	12
Objectives	3	State specific objectives, including any pre-specified hypotheses	12-13
**Methods**			
Study design	4	Present key elements of study design early in the paper	13
Setting	5	Describe the setting, locations, and relevant dates, including periods of recruitment, exposure, follow-up, and data collection	13
Participants	6	(*a*) *Cohort study*—Give the eligibility criteria, and the sources and methods of selection of participants. Describe methods of follow-up<br>*Case-control study*—Give the eligibility criteria, and the sources and methods of case ascertainment and control selection. Give the rationale for the choice of cases and controls<br>*Cross-sectional study*—Give the eligibility criteria, and the sources and methods of selection of participants	13-14
		(*b*) *Cohort study*—For matched studies, give matching criteria and number of exposed and unexposed<br>*Case-control study*—For matched studies, give matching criteria and the number of controls per case	
Variables	7	Clearly define all outcomes, exposures, predictors, potential confounders, and effect modifiers. Give diagnostic criteria, if applicable	14-15
Data sources/ measurement	8*	For each variable of interest, give sources of data and details of methods of assessment (measurement). Describe comparability of assessment methods if there is more than one group	14-15
Bias	9	Describe any efforts to address potential sources of bias	15
Study size	10	Explain how the study size was arrived at	NA
Quantitative variables	11	Explain how quantitative variables were handled in the analyses. If applicable, describe which groupings were chosen and why	14-15
Statistical methods	12	(*a*) Describe all statistical methods, including those used to control for confounding	14-15
		(*b*) Describe any methods used to examine subgroups and interactions	NA
		(*c*) Explain how missing data were addressed	NA
		(*d*) *Cohort study*—If applicable, explain how loss to follow-up was addressed<br>*Case-control study*—If applicable, explain how matching of cases and controls was addressed<br>*Cross-sectional study*—If applicable, describe analytical methods taking account of sampling strategy	14-15
		(*e*) Describe any sensitivity analyses	

Continued on next page

For peer review only - http://bmjopen.bmj.com/site/about/guidelines.xhtml

Items	Item No	Recommendation	Page No
**Results**			
Participants	13*	(a) Report numbers of individuals at each stage of study—eg numbers potentially eligible, examined for eligibility, confirmed eligible, included in the study, completing follow-up, and analysed	16
		(b) Give reasons for non-participation at each stage	16
		(c) Consider use of a flow diagram	Fig 1
Descriptive data	14*	(a) Give characteristics of study participants (eg demographic, clinical, social) and information on exposures and potential confounders	16-17
		(b) Indicate number of participants with missing data for each variable of interest	16-17
		(c) *Cohort study*—Summarise follow-up time (eg. average and total amount)	
Outcome data	15*	*Cohort study*—Report numbers of outcome events or summary measures over time	
		Case-control study—Report numbers in each exposure category, or summary measures of exposure	
		Cross-sectional study—Report numbers of outcome events or summary measures	16-17
Main results	16	(*a*) Give unadjusted estimates and, if applicable, confounder-adjusted estimates and their precision (eg, 95% confidence interval). Make clear which confounders were adjusted for and why they were included	17-20
		(*b*) Report category boundaries when continuous variables were categorized	17-20
		(*c*) If relevant, consider translating estimates of relative risk into absolute risk for a meaningful time period	NA
Other analyses	17	Report other analyses done—eg analyses of subgroups and interactions, and sensitivity analyses	17-20
**Discussion**			
Key results	18	Summarise key results with reference to study objectives	20-22
Limitations	19	Discuss limitations of the study, taking into account sources of potential bias or imprecision. Discuss both direction and magnitude of any potential bias	22
Interpretation	20	Give a cautious overall interpretation of results considering objectives, limitations, multiplicity of analyses, results from similar studies, and other relevant evidence	22-23
Generalisability	21	Discuss the generalisability (external validity) of the study results	22-23
**Other information**			
Funding	22	Give the source of funding and the role of the funders for the present study and, if applicable, for the original study on which the present article is based	28

**Give information separately for cases and controls in case-control studies and, if applicable, for exposed and unexposed groups in cohort and cross-sectional studies.*

For peer review only
